# Excited‐State Basicity Diverts the Site‐Selectivity of Aromatic Deuteration: Application to the Late‐Stage Labeling of Pharmaceuticals

**DOI:** 10.1002/anie.202500627

**Published:** 2025-05-08

**Authors:** Eva Rivera‐Chao, Javier Corpas, Giovanni Lonardi, Volker Derdau, Alessandro Ruffoni, Daniele Leonori

**Affiliations:** ^1^ Institute of Organic Chemistry RWTH Aachen University Landoltweg 1 Aachen 52056 Germany; ^2^ Integrated Drug Discovery, R&D, Sanofi Germany Industriepark Hoechst Frankfurth am Main 65926 Germany

**Keywords:** Anti‐aromaticity, Deuteration, Isotope labeling, Transient absorption spectroscopy

## Abstract

Isotope labeling, particularly with deuterium (^2^H), plays a critical role in drug discovery due to its ease of incorporation and its potential to switch unwanted metabolic transformations. Deuterium incorporation can enhance drug stability, affect pharmacokinetics, and alter metabolism pathways. Current deuterium labeling methods focus on hydrogen isotope exchange (HIE), and typically rely on the use of transition metal catalysis. Herein, we present a metal‐free approach for aromatic HIE, utilizing photoexcitation in deuterated hexafluoroisopropanol (HFIP‐*d*_1_). By exploiting the enhanced basicity of excited‐state aromatics, this method achieves selective deuteration at positions often inaccessible by traditional methods. The approach is efficient and was demonstrated across a broad number of complex drug molecules. Transient absorption spectroscopy confirms the formation of deuterated arenium ions.

## Graphical abstract

We present a metal‐free method for aromatic deuterium labeling via photoexcitation in deuterated HFIP. This approach leverages the enhanced basicity of excited‐state aromatics to achieve selective hydrogen isotope exchange at challenging positions. Demonstrated on complex drug molecules, this efficient strategy provides a valuable tool for isotope labeling, with mechanistic insights confirmed by transient absorption spectroscopy.

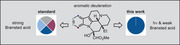

Isotope labeling has profound implications for understanding chemical reactivity, such as via kinetic isotope effect (KIE) studies,^[^ ^1^ ^]^ and has also driven significant breakthroughs in medicinal chemistry and drug discovery.^[^ ^2^, ^3^, ^4^ ^]^ In this context, deuterium (^2^H) is the preferred labeling isotope in isotopically modified drugs as in most of the metabolic processes a C–H bond must be broken.^[^ ^2^, ^3^, ^4^ ^]^ Due to the higher stability of the C–D compared to the C–H bond (1.4 kcal mol^−1^ difference) enzymatic processes can be modified if metabolic hot spots are deuterated.^[^ ^5^ ^]^ For example, recent studies have demonstrated that deuteration at specific aromatic positions in sorafenib^[^ ^6^ ^]^ and vismodegib^[^ ^7^ ^]^ results in improved pharmacokinetic and physicochemical properties, such as logP and metabolic stability, compared to the non‐deuterated drugs (Scheme 1a). Furthermore, the use of deuterium increases molecular weight, allowing for high‐sensitivity detection via mass spectrometry. This makes deuterium‐labeled compounds highly valuable as MS standards in bioanalytical evaluations, aiding in the identification, monitoring, and understanding of biochemical processes.^[^ ^8^ ^]^

**Scheme 1:**
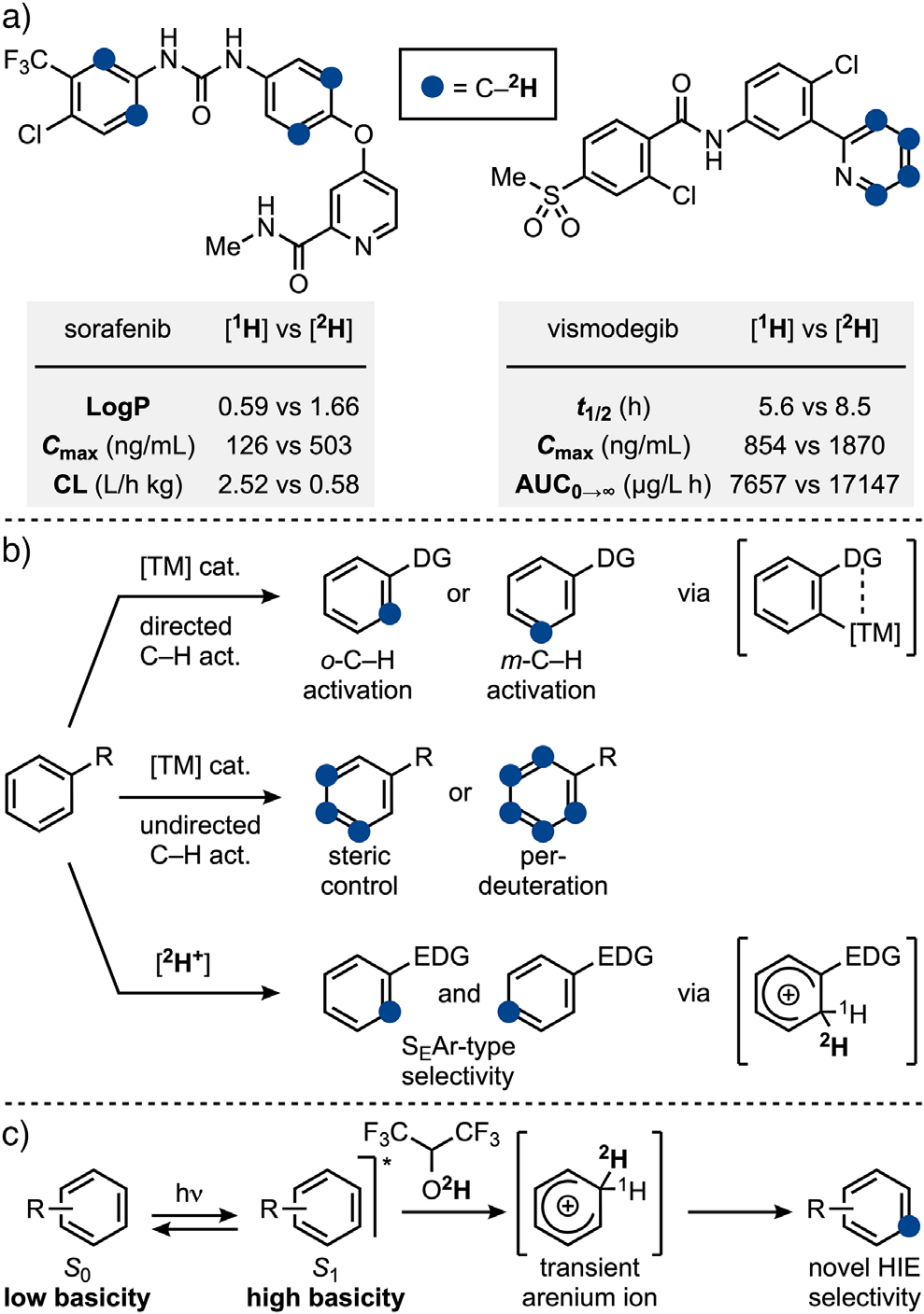
a) Aromatic deuteration can improve pharmacokinetic and physicochemical properties of drugs. b) Overview of methods for HIE on aromatics. c) This work uses aromatic photoexcitation to enable HIE using HFIP‐*d*_1_.

Among various approaches for incorporating deuterium isotopes into bioactive molecules,^[^ ^9^, ^10^, ^11^, ^12^ ^]^ the late‐stage hydrogen isotope exchange (HIE) reaction stands out as an ideal method due to its high atom economy.^[^ ^13^, ^14^ ^]^ HIE eliminates the need for deuterated synthetic precursors or the introduction of reactive functional groups for subsequent isotope incorporation (e.g., deuteration of C─X bonds). Given the ubiquity of C─H bonds in drug molecules, achieving chemoselective HIE is necessary, although it remains a significant challenge.

Homogeneous transition metal catalysis is a prominent approach for HIE of aromatics, offering high efficiency and selectivity (Scheme 1b). This strategy typically requires the presence of directing groups (R ═ DG) to facilitate C(*sp* ^*2*^)─H activation, thereby controlling the selectivity of the process.^[^ ^15^, ^16^, ^17^, ^18^, ^19^ ^]^ Nondirected HIE methods have gained significant attention as they can increase substrate generality. These processes often target sterically more accessible C(*sp* ^*2*^)─H bonds^[^ ^20^, ^21^, ^22^ ^]^ or lead to aromatic perdeuteration.^[^ ^23^, ^24^, ^25^, ^26^ ^]^

While the transition metal‐catalyzed HIEs are undoubtedly useful, the development of metal‐free variants would offer significant advantages, particularly in pharmaceutical labeling, where trace metal contamination can require extensive post‐reaction purification.^[^ ^27^, ^28^, ^29^ ^]^ However, achieving aromatic HIE without a metal catalyst presents a significant challenge. Deuterated Brønsted acids have been investigated, but the low basicity of aromatic moieties necessitates the use of super acids (p*K*a < –10, such as TfOD) to generate a transient deuterated arenium ion.^[^ ^30^, ^31^, ^32^, ^33^, ^34^ ^]^ Moreover, these methods exhibit S_E_Ar‐type *ortho*‐ and *para*‐selectivity and cannot accommodate acid‐labile functionalities.

In this manuscript, we demonstrate that transition metal‐free and Brønsted‐superacid free HIE of aromatics can be achieved by direct irradiation in deuterated hexafluoroisopropanol (HFIP‐*d*_1_) (Scheme 1c). In this regard, photoinduced HIE is typically achieved at C(sp^3^)─H sites due to the lower bond dissociation energy compared to C(sp^2^)─H bonds. Indeed, the deuteration of aromatics under photochemical conditions has been only achieved using “super reductive” photoredox catalysts via an interrupted Birch pathway.^[^ ^35^ ^]^ In contrast, our approach leverages photoexcitation to transiently enhance the basicity of the aromatic^[^ ^35^, ^36^, ^37^, ^38^, ^39^, ^40^, ^41^, ^42^, ^43^ ^]^ independently from the redox potential of the substrate. This feature allows for electrophilic deuteration from a weakly acidic medium like HFIP‐*d*_1_. Since the properties of excited‐state aromatics differ significantly from their ground state counterparts, this method enables the selective labeling of specific positions that are often elusive to other strategies. This effectively by‐passes the need for DGs or strong steric bias.

At the outset of our work, we were inspired by McClelland's and Shizuka pioneering work on the differing behaviors of ground‐state versus excited‐state aromatics in the presence of proton sources (Scheme 2a).^[^ ^44^, ^45^, ^46^, ^47^, ^48^, ^49^ ^]^ Specifically, these studies confirmed that upon photoexcitation, *S*_1_‐arenes (π,π*), such as 1,3‐dimethoxybenzene **A**, underwent protonation by weak Brønsted acids like HFIP (p*K*a = 9.3). This process led to the formation of arenium ions (e.g., **A**─H^+^), which were observed and characterized through laser‐flash photolysis. According to this work, since photoexcited aromatics experience a dramatic increase in basicity, the utilization of weakly acidic deuterium sources should overcome many of the limitations of ground‐state chemistry with super Brønsted acids. However, despite the potential of this photobasicity concept for aromatic functionalization, is far from being established.

**Scheme 2:**
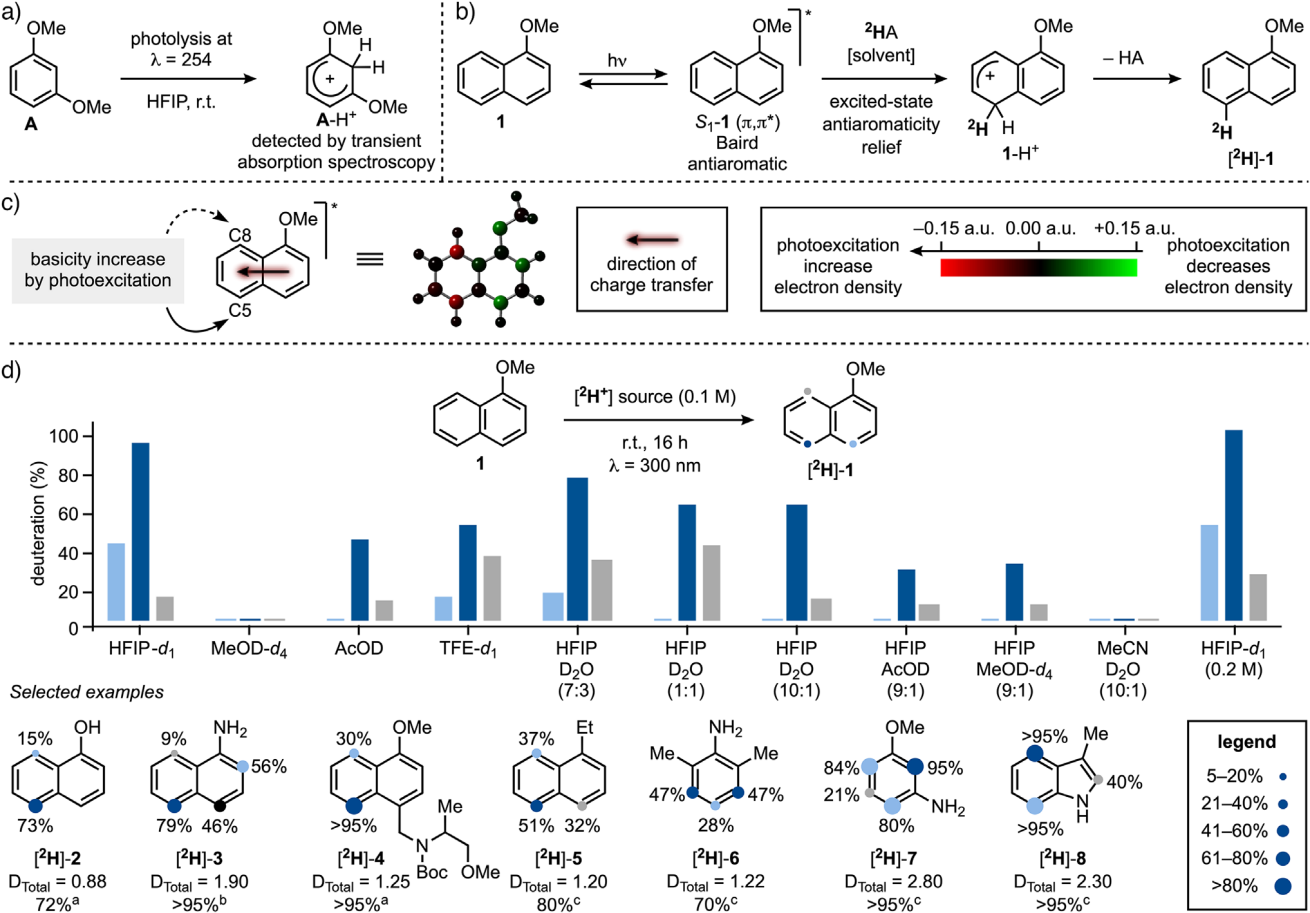
a) McClelland experiments by laser‐flash photolysis. b) Proposed mechanism for photochemical HIE. c) Change in aromatic basicity upon charge‐transfer. d) Optimization of the photochemical deuteration of **1** and other selected examples. ^a^ λ = 300 nm. ^b^ λ = 370 nm. ^c^ λ = 254 nm.

Our approach for transition metal‐free photochemical HIE of aromatics is described in Scheme 2b using 1‐*OMe*‐naphthalene **1** as the model substrate. Direct photoexcitation would populate its *S*_1_ excited‐state that has π,π* configuration.^[^ ^50^ ^]^ According to Baird's rules,^[^ ^51^, ^52^, ^53^, ^54^ ^]^ this electronic state exhibits anti‐aromatic character, which increases its associated basicity.^[^ ^30^, ^31^, ^32^, ^33^, ^34^ ^]^ Hence, the reaction with an electrophilic deuterium source would relieve excited‐state anti‐aromaticity, leading to the formation of a ground‐state deuterated arenium ion **1**‐H^+^.^[^ ^55^, ^56^ ^]^ This intermediate, upon deprotonation and rearomatization, would yield either the deuterated product [^2^H]‐**1** or regenerate the starting aromatic **1**. Running the reaction in a deuterated medium should effectively drive the formation of [^2^H]‐substituted products.

A potentially valuable aspect of this approach lies in its selectivity. Indeed, upon photoexcitation, the redistribution of electronic density around the aromatic ring should alter the preferred sites of electrophilic deuteration, compared to traditional thermal S_E_Ar reactivity (Scheme 2c).^[^ ^57^, ^58^, ^59^ ^]^ In the case of **1**, photoexcitation leads to charge transfer (CT) from the OMe group to the distal aromatic unit increasing electron density at the remote C5 and C8. This opens the possibility of targeting selectively distal aromatic positions that are inaccessible through thermal or metal‐catalyzed processes.

We began studying the feasibility of this approach using **1** and HFIP‐*d*_1_ (CAS: 38701–73–4) under 300 nm irradiation at room temperature (Scheme 2d). Gratifyingly, we observed high deuterium incorporation, predominantly at the distal C5 position, along with moderate incorporation at C4 and C8, in quantitative yield. Crucially, under these conditions no C2 deuteration took place, despite its intrinsically activated nature (ground state). The reaction performed equally well under air, supporting the hypothesis that deuteration occurs from the *S*_1_ excited‐state. Interestingly, to the best of our knowledge, the selectivity displayed by this process is orthogonal to all other reported HIE strategies.

Other deuterated solvents, such as AcOD or TFE‐*d*_1_, provided a lower degree of incorporation, while the significantly less acidic MeOD‐*d*_4_ resulted in no D‐incorporation. Interestingly, mixtures of non‐deuterated HFIP with D_2_O, MeOD‐*d*_4_, or AcOD still facilitated deuteration, though with reduced efficiency. Notably, when D_2_O was combined with a nonacidic solvent, such as CH_3_CN, no HIE occurred, further confirming the crucial role of the medium in achieving high reactivity. Additionally, the amount of HFIP‐*d*_1_ could be reduced to 47 equivalents (0.2 M) without compromising the resulting HIE.

We then briefly evaluated the generality of the process using other aromatics. Unprotected 1‐naphthol (**2**) and 1‐naphthylamine (**3**) exhibited similar reactivity, favoring C5 HIE. This approach also allows for the tolerance of acid‐labile functionalities, such as *N*‐Boc group (**4**).

An intrinsic limitation of this methodology is its reliance on electron‐rich aromatics. This is due to the requirement of the CT to increase the electron density at the π system. Contrarily, the presence of electron‐withdrawing groups prevents deuteration due to CT from the aromatic moiety to these functionalities. Nevertheless, we successfully applied it to 1‐ethyl‐naphthalene (**5**), although naphthalene itself remained unreactive. We then screened benzene derivatives, successfully extending the chemistry to aniline derivatives **6** and **7** as well as 3‐Me‐indole (**8**). In this latter case, HIE took place preferentially at the C4 and C7 positions, which would be difficult to target by methods based on alternative activation pathways. Crucially, no background reactivity was overserved upon treatment of these derivatives (**1**–**8**), as well as the ones reported in Scheme 4, in HFIP‐*d_1_* in the dark.^[^ ^60^ ^]^ This means aromatic deuteration is the exclusively result of basicity increase upon photoexcitation. In the case of **3** and **7**, thermal HIE occurred but with different site selectivity (see the Supporting Information for more details).

To provide direct evidence of the generation of deuterated arenium intermediates, we performed transient absorption spectroscopy (TAS) studies (Scheme 3a–f). Upon irradiation at 266 nm of **1** in HFIP, we detected the formation of **1**‐H^+^, which showed two absorption bands with identical decay (*τ* ∼ 1600 ns) centered at *λ* = 360 and 550 nm. The use of HFIP‐*d*_1_ resulted in an almost identical transient (**1**‐H^+^), which however showed a slightly shorter lifetime (*τ* ∼ 1300 ns) (Scheme 3a and b).^[^ ^44^, ^45^ ^]^

**Scheme 3:**
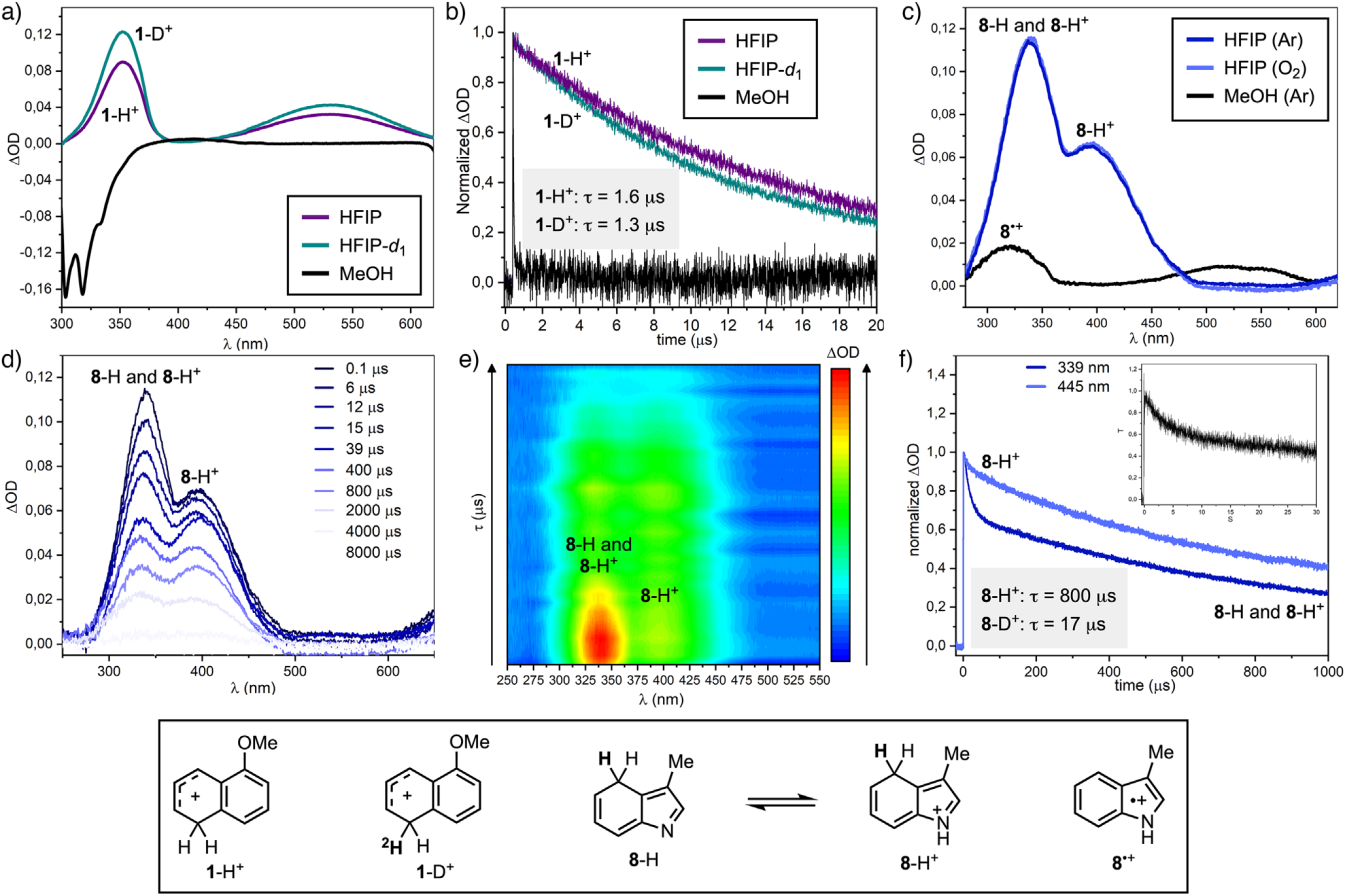
a) Transient absorption spectra for **1** (150 µM) in HFIP, HFIP‐*d*_1_, or MeOH as solvents under Ar. b) Corresponding decay traces monitored at 550 nm. c) Transient absorption spectra of **8** (150 µM) in HFIP or MeOH as solvents under Ar, and in HFIP under O_2_. d) Transient absorption spectra of **8** (150 µM) in HFIP at different time intervals under Ar. e) Contour plot of the full 2D transient absorption data set of **8** (150 µM) in HFIP under Ar. f) Corresponding decay traces monitored at 339 and 440 nm (inset: decay of **8** in MeOH at 500 nm).

To further demonstrate the unique effectiveness of HFIP to lead to aromatic protonation/deuteration (see Scheme 2d), we performed identical TAS experiments using MeOH as the solvent. In accordance with the synthetic experiments, arenium ion formation (**1**‐H^+^) did not take place and photobleaching was observed below 400 nm (absorption of **1** in the ground state) (Scheme 3a and b).

Similar TAS experiments were also run for **8** in both HFIP (Ar and O_2_) and MeOH. The TAS in MeOH shows two bands centered at 320 and 520 nm which belong to the formation of the radical cation **8** ^·+^ (*τ* ∼ 17 µs) (Scheme 3c).^[^ ^46^, ^48^ ^]^ In contrast, experiments run in HFIP revealed the formation of a new transient showing two main absorption bands centered at *λ* = 339 nm and 400 nm. Following the decay traces at 339 and 440 nm it was possible to observe overlap of two species (Scheme 3d and e) corresponding to the areniums **8**‐H^+^ and **8**‐H, which are in equilibrium^[^ ^47^ ^]^ (note that the same species protonated at C7 should be also present in solution, leading to the same transient signal, not shown). These measurements were run under both Ar and O_2_ to give the same absorption profile, thus ruling out the intermediacy of triplet or radical intermediates. These two species had rather different decay profile with *τ*(**8**‐H) ∼ 17 µs and *τ*(**8**‐H^+^) ∼ 800 µs (Scheme 3f).

Our optimized conditions for HIE involve the irradiation of aromatics in HFIP‐*d_1_* at room temperature, under air, and without the need for stirring. Additionally, chromatographic purification is generally unnecessary, making this a practical and straightforward protocol for the selective deuteration of aromatic compounds. With these conditions in hand, we decided to benchmark the reactivity across a series of pharmaceutically relevant and structurally complex molecules. As we will discuss, our method typically offers deuteration selectivity that is orthogonal to other strategies.

We began by evaluating several naphthalene‐containing drugs (**9**–**11**) (Scheme 4). In line with our optimization results, propranolol (**9**), dapoxetine (**10**), and naftopidil (**11**) demonstrated efficient HIE with strong selectivity for the distal C5 position and tolerance of polar groups such as free alcohol and amines (primary, tertiary, and aniline). Naftopidil (**11**), in particular, exhibited selective HIE of the naphthalene core over the *ortho*‐disubstituted benzene substituent, likely due to its higher absorbance at the irradiation wavelength.

**Scheme 4:**
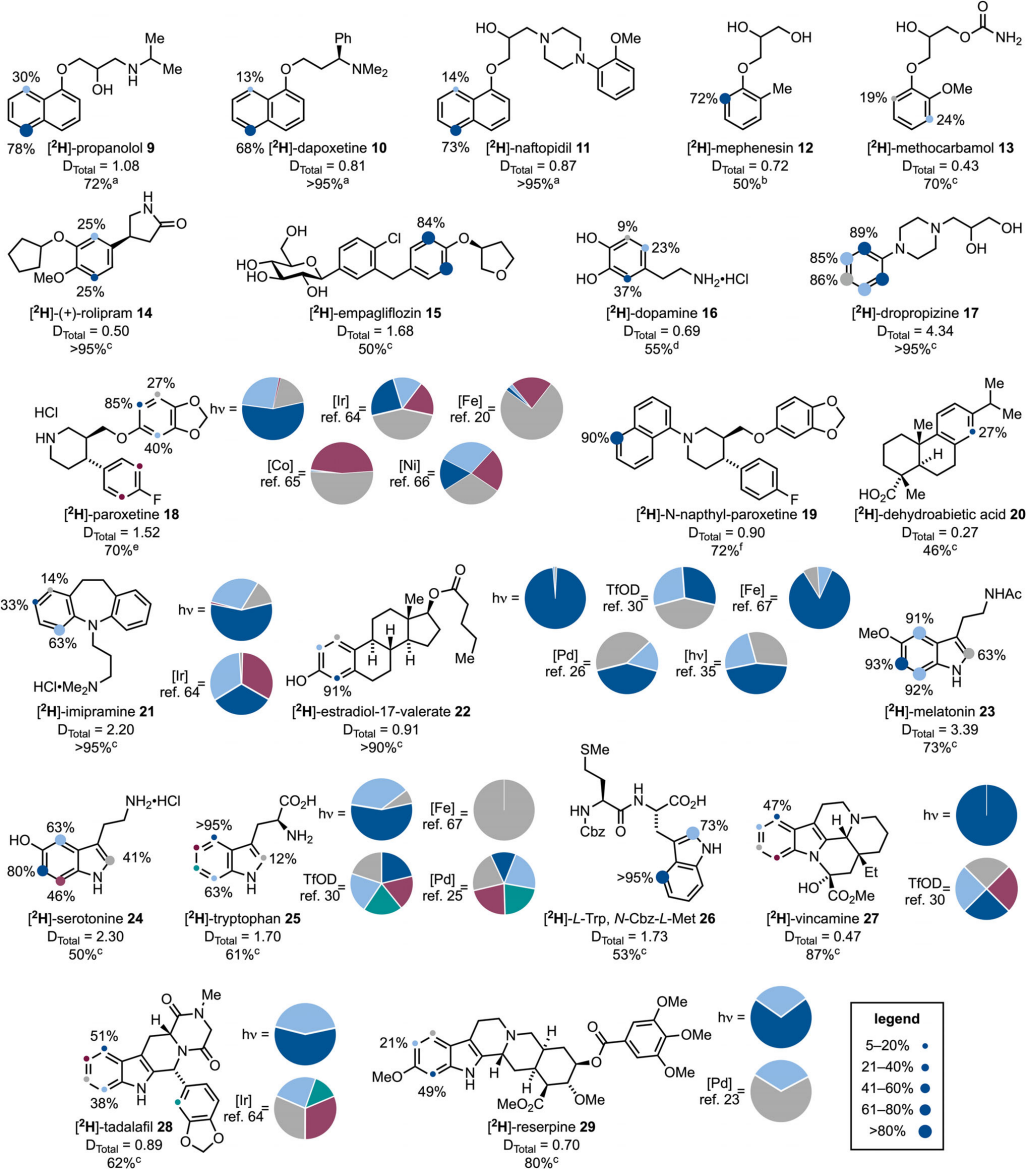
Reaction scope for the photochemical deuteration of aromatics. ^a^ 300 nm. ^b^ 254 nm, 94 equiv. HFIP‐*d_1_*. ^c^ 254 nm. ^d^ 254 nm, HFIP/D_2_O (7/3, 0.1 M). ^e^ 310 nm. ^f^ 370 nm. ^2^H incorporation was determined by ^1^H NMR spectroscopy and further confirmed by HRMS.

Next, we explored the HIE of electron‐rich benzene derivatives, given the ubiquity of these motifs in pharmaceutical and agrochemical products. Notably, approximately 60% of small‐molecule drugs approved by the FDA in 2020 contained a phenolic‐type group.^[^ ^61^ ^]^ Mephenesin (**12**) exhibited high and selective C6 deuterium incorporation, demonstrating tolerance of a free 1,2‐diol functionality, which would typically undergo rearrangement or other side reactions under acidic conditions. Methocarbamol (**13**) and rolipram (**14**) also showed compatibility with carbamate and lactam functionalities, as well as 1,2‐dialkoxy aromatic substitution patterns, which are generally unstable under acidic photolysis due to competitive hydrolysis.^[^ ^62^, ^63^ ^]^ Empagliflozin (**15**), which contains two aromatic units and a glucose residue, underwent selective HIE of the most electron‐rich arene, specifically at the position *ortho* to the ether substituent. Dopamine•HCl (**16**) underwent moderate deuteration, preferentially at C5. It is worth noting that amines do not need to be used as hydrochloride salts for HIE, as demonstrated by dropropizine (**17**), which underwent efficient perdeuteration. Control experiments confirmed that all HIE processes were photochemically driven (see the Supporting Information for more details).

Paroxetine•HCl (**18**), which contains two aromatic groups, provided an interesting selectivity outcome compared to literature reports. Previous methods based on [Ir],^[^ ^64^ ^]^ [Fe],^[^ ^20^ ^]^ [Co],^[^ ^65^ ^]^ and [Ni]^[^ ^66^ ^]^ catalysis generally result in unselective labeling of both aromatic rings or target positions activated by the fluorine atom and [1,3]dioxole group. In contrast, our method led to selective functionalization at C6 of the more electron‐rich ring. Moreover, protecting the piperidine with a 1‐naphthyl group (**19**) shifted the selectivity, directing deuteration to the C5 position of the naphthalene unit. Dehydroabietic acid (**20**), a complex terpene‐like structure with a free carboxylic acid and three aromatic positions, underwent selective C8 deuteration, albeit with moderate efficiency.

We also revisited the deuteration of imipramine (**21**) and estradiol‐17‐valerate (**22**), which have been previously studied.^[^ ^26^, ^30^, ^64^, ^67^ ^]^ In the case of imipramine, our method enabled preferential deuteration at C4 and C6 positions. Estradiol‐17‐valerate displayed striking orthogonal selectivity, with complete C4 deuteration—a notable divergence from the selectivity observed with superacids,^[^ ^30^ ^]^ [Fe]^[^ ^67^ ^]^ and [Pd]^[^ ^26^ ^]^ catalysis, and recent photoredox methods.^[^ ^35^ ^]^

We then evaluated indole‐containing pharmaceuticals. Melatonin (**23**) and serotonin (**24**) underwent effective aromatic perdeuteration with high and moderate efficiency, respectively. *L*‐Tryptophan (**25**), which has been extensively studied for deuteration, showed a promising outcome: while [Fe] catalysis^[^ ^67^ ^]^ leads to selective C2 deuteration and super Brønsted superacids^[^ ^30^ ^]^ and [Pd] catalysis^[^ ^25^ ^]^ are unselective, our method favored C4 (95%) and C7 labeling. Interestingly, embedding *L*‐tryptophan into a dipeptide system (**26**) maintained high C4 selectivity, with C2 labeling instead of C7. This example also showed tolerance of a thioether motif.

Furthermore, vincamine (**27**), tadalafil (**28**), and reserpine (**29**) showcased the orthogonal selectivity of our method compared to mainstream approaches. For instance, treatment of vincamine with Brønsted superacids^[^ ^30^ ^]^ results in unselective deuteration, whereas our method exhibited exclusive C4 selectivity. [Ir] catalysis^[^ ^64^ ^]^ targets C5, C6, and C7 of the indole moiety and C3 of the benzodioxole group in tadalafil, but our method shifted selectivity to C4 and C7. Lastly, treatment of reserpine with [Pd] catalysis^[^ ^23^ ^]^ generally targets C4 and C5, but our approach diverted selectivity to C7 and C5.

This study demonstrates a novel, metal‐free method for the selective deuteration of aromatic compounds using photoexcitation in deuterated HFIP‐*d*_1_. By harnessing the unique properties of singlet excited‐state aromatics, this approach enables HIE avoiding the use of transition metal catalysts or superacids. The method achieves high selectivity, particularly at positions that are challenging for other strategies, and exhibits broad substrate compatibility, including complex pharmaceutical molecules. This technique offers significant advantages for drug development and isotope labeling, providing a practical and efficient alternative for the selective incorporation of deuterium into complex molecules. Future efforts will be needed to enhance the overall labeling efficiency, ensuring this process becomes viable for the preparation of of MS standards.

## Supporting Information

Characterization for all new labeled compounds; additional experimental details; photophysical measurements; materials and methods, including photographs of experimental setups are included in the Supporting Information file.

## Conflict of Interests

V.D. is an employee of Sanofi and may hold shares and options of the company. All other authors declare no competing financial interest.

## Supporting information



Supporting Information

## Data Availability

The data that support the findings of this study are available from the corresponding author upon reasonable request.
