# Supplementary material for: Excited‐State Basicity Diverts the Site‐Selectivity of Aromatic Deuteration: Application to the Late‐Stage Labeling of Pharmaceuticals
Source: Angew Chem Int Ed Engl. 2025 May 8;64(25):e202500627. doi: 10.1002/anie.202500627 (PMC12171340; doi:10.1002/anie.202500627)
Supplement: Supplementary file 1 — Supporting Information [file ANIE-64-e202500627-s001.pdf]

Excited-State Basicity Diverts the Site-Selectivity of Aromatic Deuteration: Application to the Late-Stage Labelling of Pharmaceuticals.

Eva Rivera-Chao,[‡a] Javier Corpas,[‡a] Giovanni Lonardi,[a] Volker Derdau,[b] Alessandro Ruffoni,[a] and Daniele Leonori*[a]

[a]*Institute of Organic Chemistry, RWTH Aachen University, Aachen 52056, Germany*

[b]*Integrated Drug Discovery, R&D, Sanofi Germany, Industriepark Hoechst, 65926, Frankfurth am Main, Germany.*

[‡]These authors contributed equally

daniele.leonori@rwth-aachen.de

Table of Contents

**1.**	**General experimental details**	**3**
**2.**	**Reaction optimization**	**5**
**3.**	**General procedure for photochemical HIE**	**6**
**4.**	**Pictures of reaction set-up**	**7**
**5.**	**Spectra of lamps**	**9**
**6.**	**Substrate scope**	**10**
**7.**	**Gram-Scale Experiment**	**72**
**8.**	**Control experiments**	**73**
**9.**	**Photophysical studies**	**74**

1. General experimental details

All chemicals were used directly without purification. All air and moisture sensitive reactions were carried out under nitrogen atmosphere using standard Schlenk manifold technique. All solvents were bought from Acros as 99.8% purity. ^{1}H and ^{13}C Nuclear Magnetic Resonance (NMR) spectra were acquired at various field strengths as indicated and were referenced to $CHCl_3$ (7.26 and 77.16 ppm for ^{1}H and ^{13}C respectively). ^{1}H NMR coupling constants are reported in Hertz and refer to apparent multiplicities and not true coupling constants. Data are reported as follows: chemical shift, integration, multiplicity (s = singlet, bs = broad singlet, d = doublet, bd = broad doublet, t = triplet, q = quartet, p = pentet, m = multiplet, dd = doublet of doublets, etc.). ^{19}F-NMR spectra were recorded and reported unreferenced. High-resolution mass spectra were obtained using a JEOL JMS-700 spectrometer or a Fissions VG Trio 2000 quadrupole mass spectrometer. Spectra were obtained using electron impact ionization (EI) and chemical ionization (CI) techniques, or positive electrospray (ES). Analytical TLC: aluminum backed plates pre-coated (0.25 mm) with Merck Silica Gel 60 F254. Compounds were visualized by exposure to UV-light or by dipping the plates in permanganate ($KMnO_4$) or cerium molybdate stain followed by heating. Flash column chromatography was performed using Merck Silica Gel 60 (40–63 μm). Absorption and emission spectra were obtained using an Horiba Duetta spectrometer and 1 mm High Precision Cell made of quartz from Hellma Analytics. All mixed solvent eluents are reported as v/v solutions. The LEDs used are Kessil PR 160 370 nm. Reactions irradiated at 254 nm and 300 nm were carried out in a Rayonet photoreactor equipped with the corresponding lamps (8 W). All the reactions were conducted in CEM 10 mL glass microwave tubes or quartz vials.

Absorption spectra were obtained using a Shimadzu (UV-2600) UV-vis spectrophotometer and a quartz cuvette (10 mm x 10 mm).

Laser Flash Photolysis (LFP) measurements were carried out with a LP980 equipment (Edinburgh Instruments) using a Q-switched Nd:YAG laser (Quantel). The selected excitation wavelength was 266 nm with single low energy pulses of 10 mJ/pulse of ca. 5 ns duration while a pulsed xenon flash lamp (150 W) was employed as probe source. The signal was recorded with a PMT detector to obtain the temporal profile and with a ICCD camera to measure the transient spectra (20 laser pulse average). The samples were measured employing quartz

cuvettes with four clear sides (10 mm x 10 mm) and degasses for 15 min with Ar (or O_2) before acquisition. All transient signals were recorded at room temperature.

2. Reaction optimization

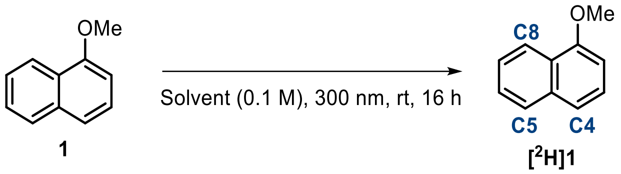

In an oven-dried 8 mL microwave vial, 1-methoxynaphthalene **1** (15 µL, 0.1 mmol, 1.0 equiv.) was dissolved in *solvent* (1.0 mL). The vial was capped with a Supelco aluminium crimp seal with septum (PTFE/butyl). The vial was placed in the photoreactor (300 nm) and irradiated at room temperature with a fan for the indicated period of time. The tube was opened, and the solvent evaporated. A solution of trichloroethylene (0.1 mL, 1.0 M in $CDCl_3$) was added, the crude was solubilised in $CDCl_3$ (0.6 mL) and the mixture analysed by ^{1}H NMR spectroscopy to obtain the ^{2}H incorporation and the NMR yield.

Table S1. Evaluation of solvent

Entry	Solvent	^{2}H% C4	^{2}H % C5	^{2}H % C8
**1**	HFIP-d_1	38	87	12
**2**	MeOD-d_4	-	-	-
**3**	AcOD	-	40	10
**4**	CF_3CH_2OD	12	47	32
**5**	HFIP/D_2O (7:3)	14	70	30
**6**	HFIP/ D_2O (1:1)	-	57	37
**7**	HFIP/ D_2O (10:1)	-	57	11
**8**	HFIP/AcOD	-	25	8
**9**	HFIP/ MeOD-d_4	-	28	8
**10**	MeCN/D_2O	-	-	-
**11**[b]	HFIP-d_1	36	90	20
**12**[c]	HFIP-d_1	47	93	23

[a] Determined in the reaction crude by ^{1}H NMR spectroscopy employing trichloroethylene as an internal standard. [b] Under argon atmosphere. [c] 0.2 M.

3. General procedure for photochemical HIE

General procedure 1 – GP1

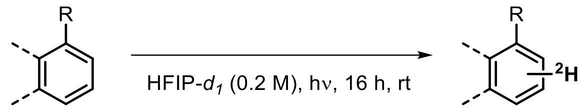

An oven-dried 8 mL quartz vial was charged with the aromatic substrate (0.1 mmol, 1.0 equiv.) and HFIP-d_1 (0.5 mL, 47 equiv.). The vial was placed in the photoreactor (254 or 300 nm) and irradiated for 16 h at room temperature with a fan. The tube was opened, and the solvent evaporated. A solution of trichloroethylene (0.1 mL, 1.0 M in $CDCl_3$) was added, the crude was solubilised in $CDCl_3$ (0.6 mL) and the mixture analysed by ^{1}H NMR spectroscopy and HRMS to obtain the ^{2}H incorporation and the NMR yield.

General procedure 2 – GP2

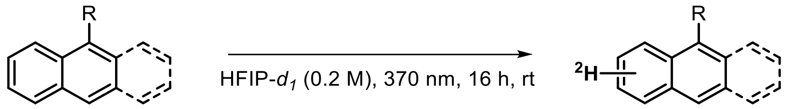

An oven-dried 8 mL microwave was charged with the aromatic substrate (0.1 mmol, 1.0 equiv.) and HFIP-d_1 (0.5 mL, 47 equiv.). The vial was placed under the lamp (PR160-370nm Gen 2 Kessil lamp) and the distance from the lamp to the bottom of the vial was set to 3.5 cm. The mixture was stirred at room temperature for 16 h with a fan. The tube was opened, and the solvent evaporated. A solution of trichloroethylene (0.1 mL, 1.0 M in $CDCl_3$) was added, the crude was solubilised in $CDCl_3$ (0.6 mL) and the mixture analysed by ^{1}H NMR spectroscopy and HRMS to obtain the ^{2}H incorporation and the NMR yield.

General procedure 3 – GP3

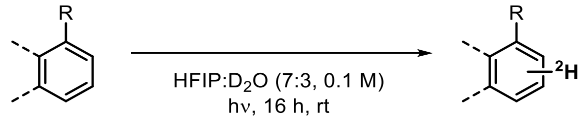

An oven-dried 8 mL quartz vial was charged with the aromatic substrate (0.1 mmol, 1.0 equiv.), HFIP (0.7 mL) and D_2O (0.3 mL). The vial was placed in a Rayonet photoreactor (254 or 300 nm) or under the lamp (PR160-370nm Gen 2 Kessil lamp) and irradiated for 16 h at room temperature with a fan. The tube was opened, and the solvent evaporated. A solution of trichloroethylene (0.1 mL, 1.0 M in $CDCl_3$) was added, the crude was solubilised in $CDCl_3$ (0.6 mL) and the mixture analysed by ^{1}H NMR spectroscopy and HRMS to obtain the ^{2}H incorporation and the NMR yield.

4. Pictures of reaction set-up

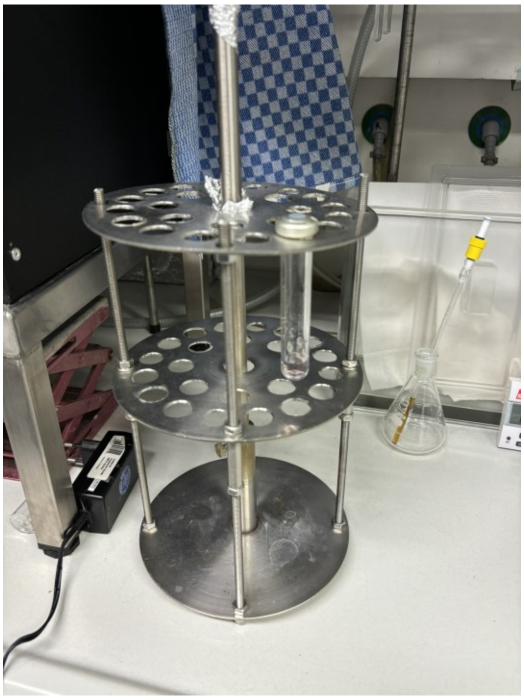

Figure S1. Microwave vial holder for photochemical reactions under 300 nm.

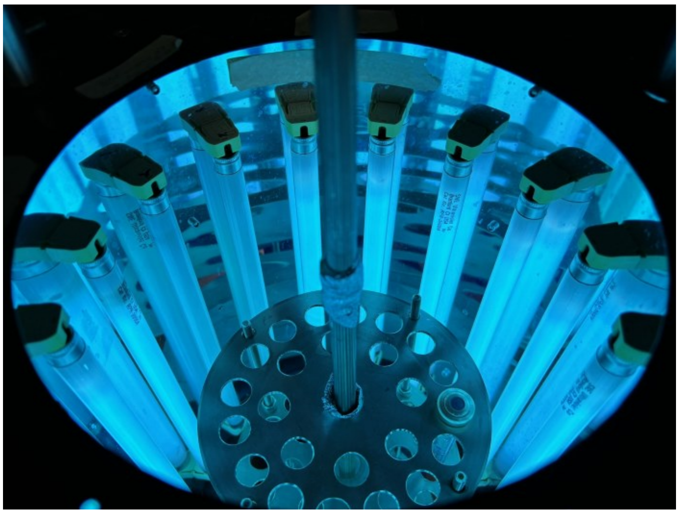

Figure S2. Photochemical set up for reactions run under 300 nm employing a Rayonet photoreactor.

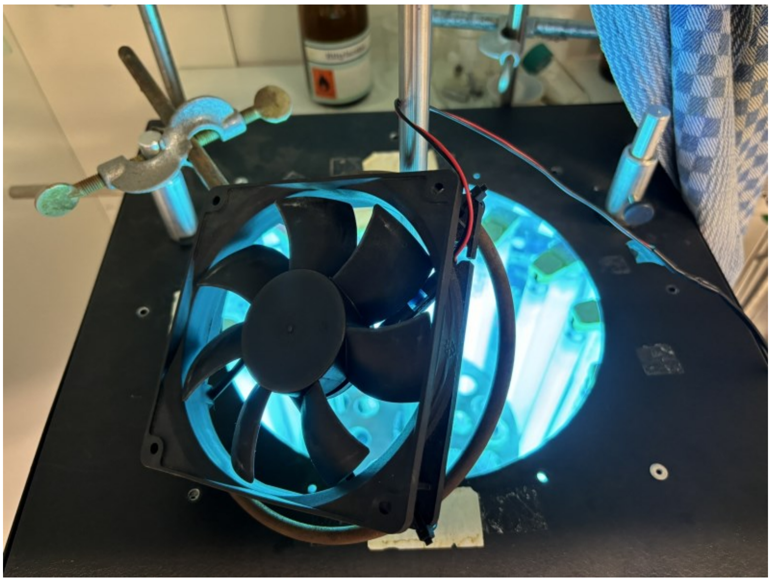

Figure S3. Cooling set up for photochemical reactions run under 300 nm employing a Rayonet photoreactor.

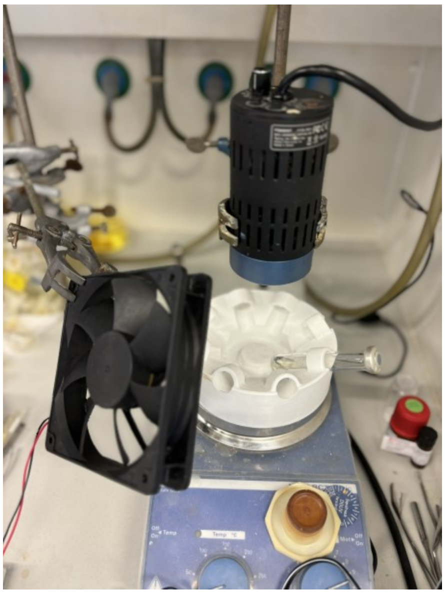

Figure S4. Set up for photochemical reactions under 370 nm with a Kessil lamp.

5. Spectra of lamps

The spectra of the Kessil lamps were taken from the Kessil website: https://www.kessil.com/products/science_PR160L.php

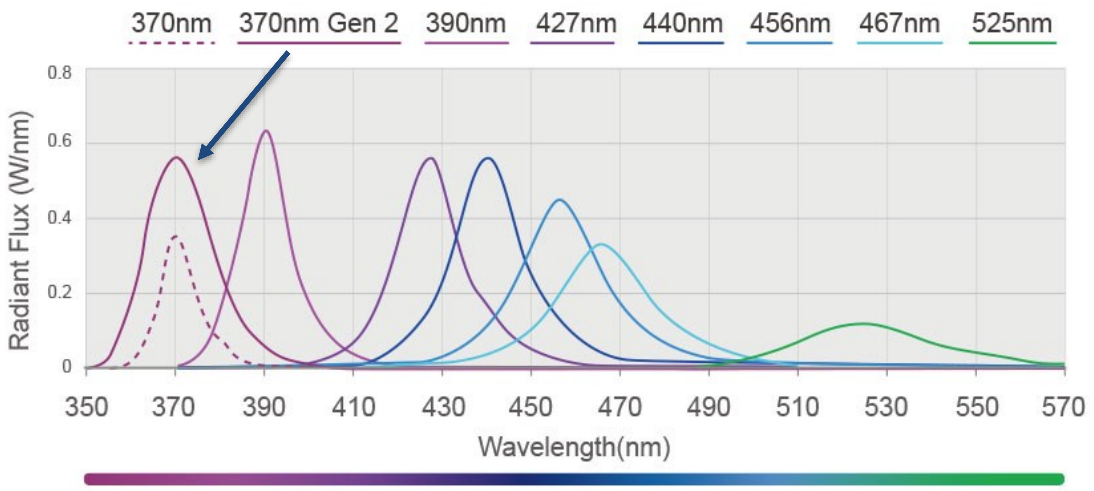

Figure S5.

6. Substrate scope

[^{2}H]1-Methoxynaphthalene (1)

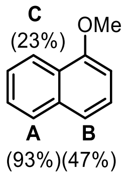

Obtained from 1-methoxynaphthalene following procedure **GP1** [300 nm]. (>95%, D_{Total} = 1.63); **^{1}H NMR (600 MHz, CD_3CN)** δ 8.23 (0.77H, d, *J*=8.2 Hz, labeled, 23% D), 7.87 (0.07H, d, *J*=8.0 Hz, labeled, 93% D), 7.57–7.49 (2H, m), 7.48 (0.53H, d, *J*=8.3 Hz, labeled, 47% D), 7.47–7.44 (1H, m), 6.95 (1H, d, *J*=7.5 Hz), 4.02 (3H, s); **^{13}C NMR (151 MHz, CD_3CN)** δ 156.2, 135.4, 128.4–127.8 (m, labeled), 127.4, 127.1, 126.3, 126.2, 122.5–122.1 (m, labeled), 120.9–120.5 (m, labeled), 105.0, 56.2; **HRMS** (EI) Calculated for $C_{11}H_{10}O$ $[M]^+$ 158.0726 found 158.0723 (4%); Calculated for $C_{11}H_9DO$ $[M]^+$ 159.0789 found 159.0789 (46%); Calculated for $C_{11}H_8D_2O$ $[M]^+$ 160.0858 found 160.0850 (44%); Calculated for $C_{11}H_7D_3O$ $[M]^+$ 161.0915 found 161.0916 (6%); D_{Total} = 1.52 calculated by HRMS.

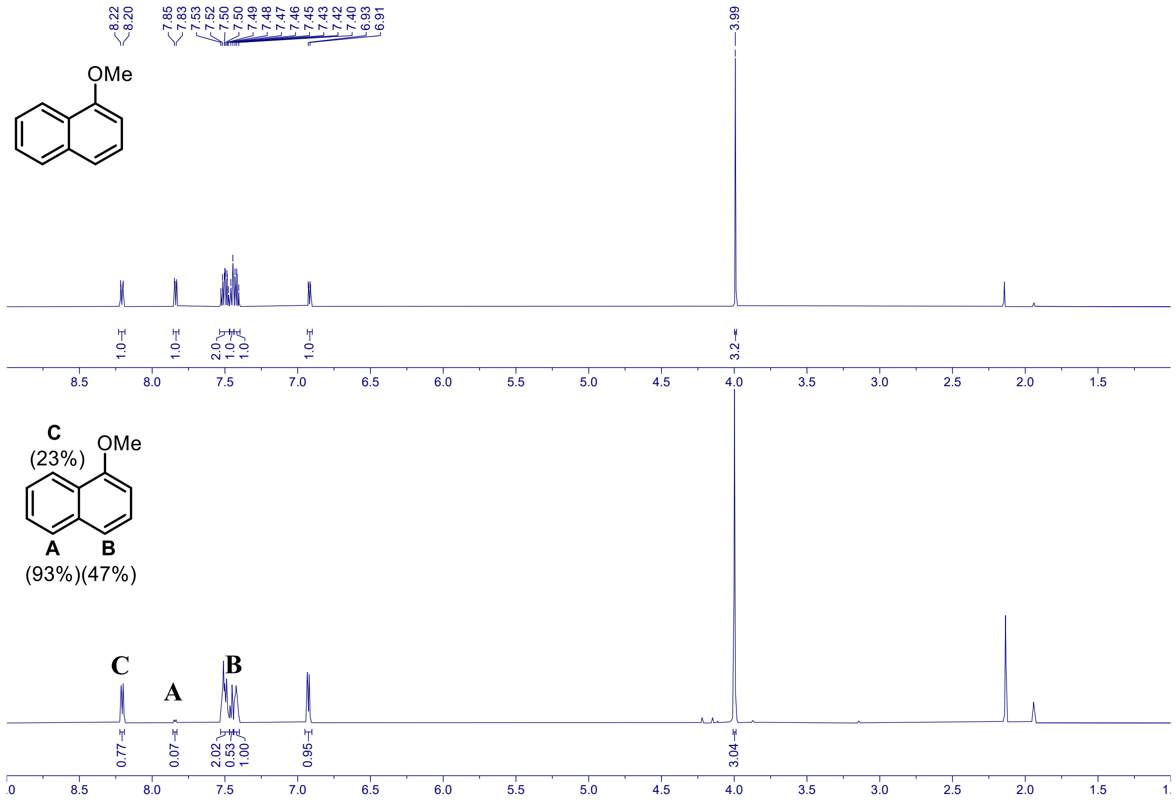

Figure S6. Stacked ^{1}H NMR (600 MHz, CD_3CN) spectra of **1**: natural abundance (top) and labelled (bottom).

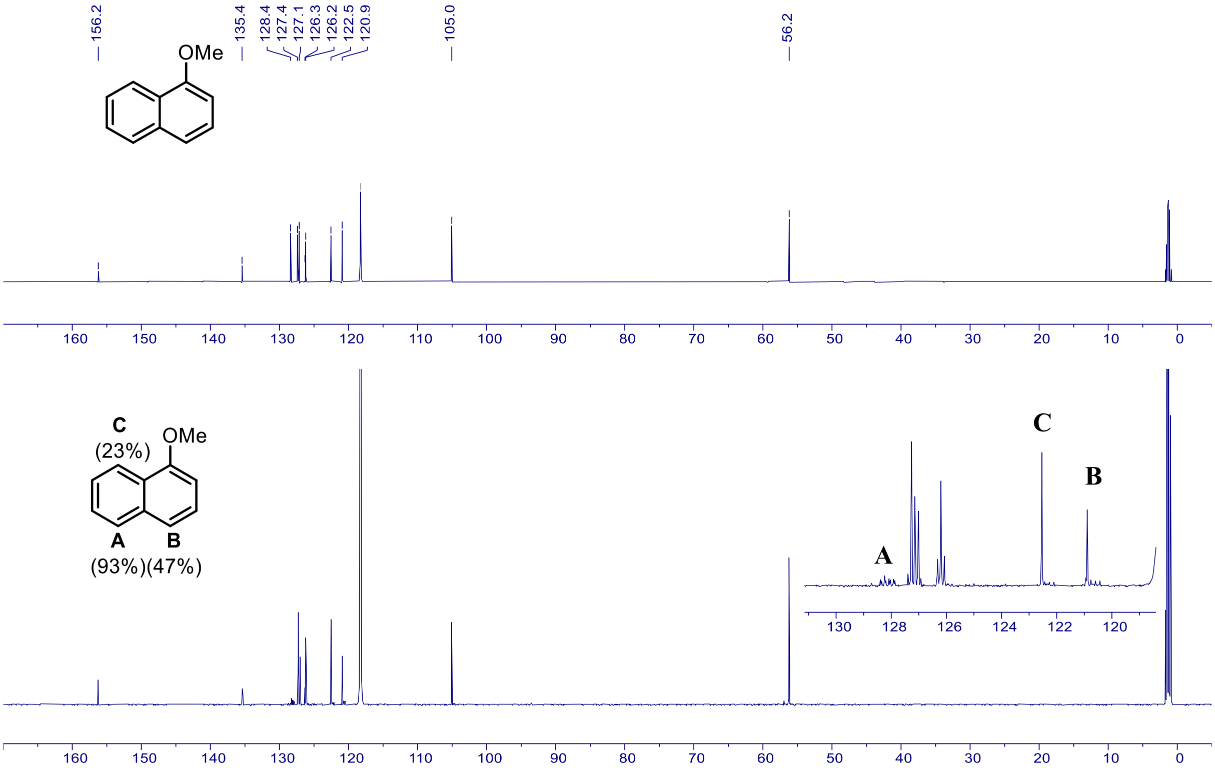

Figure S7. Stacked ^{1}H NMR (151 MHz, CD_3CN) spectra of **1**: natural abundance (top) and labelled (bottom).

[^{2}H]1-Naphthol (2)

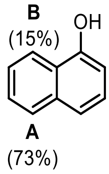

Obtained from 1-naphthol following procedure **GP1**. Purification by prep TLC. [300 nm]. (72%, D_{Total} = 0.88); **^{1}H NMR (600 MHz, $CDCl_3$)** δ 8.27–8.15 (0.85H, m, <u>labeled, 15% D</u>), 7.87–7.79 (0.27H, m, <u>labeled, 73% D</u>), 7.53–7.41 (3H, m), 7.31 (1H, t, *J*=7.8 Hz), 6.82 (1H, d, *J*=7.4 Hz), 5.35 (1H, bs); **^{13}C NMR (151 MHz, $CDCl_3$)** δ 151.5, 134.9, 127.8–127.3 (<u>m, labeled</u>), 126.6, 126.0, 125.4, 124.4, 121.7–121.2 (<u>m, labeled</u>), 120.9, 108.7; **HRMS** (EI) Calculated for $C_{10}H_8O$ $[M]^+$ 144.0570 found 144.0569 (19%); Calculated for $C_{10}H_7DO$ $[M]^+$ 145.0632 found 145.0631 (60%); Calculated for $C_{10}H_6D_2O$ $[M]^+$ 146.0695 found 146.0695 (18%); ($C_{10}H_5D_8O$ 3%); D_{Total} = 1.05 calculated by HRMS.

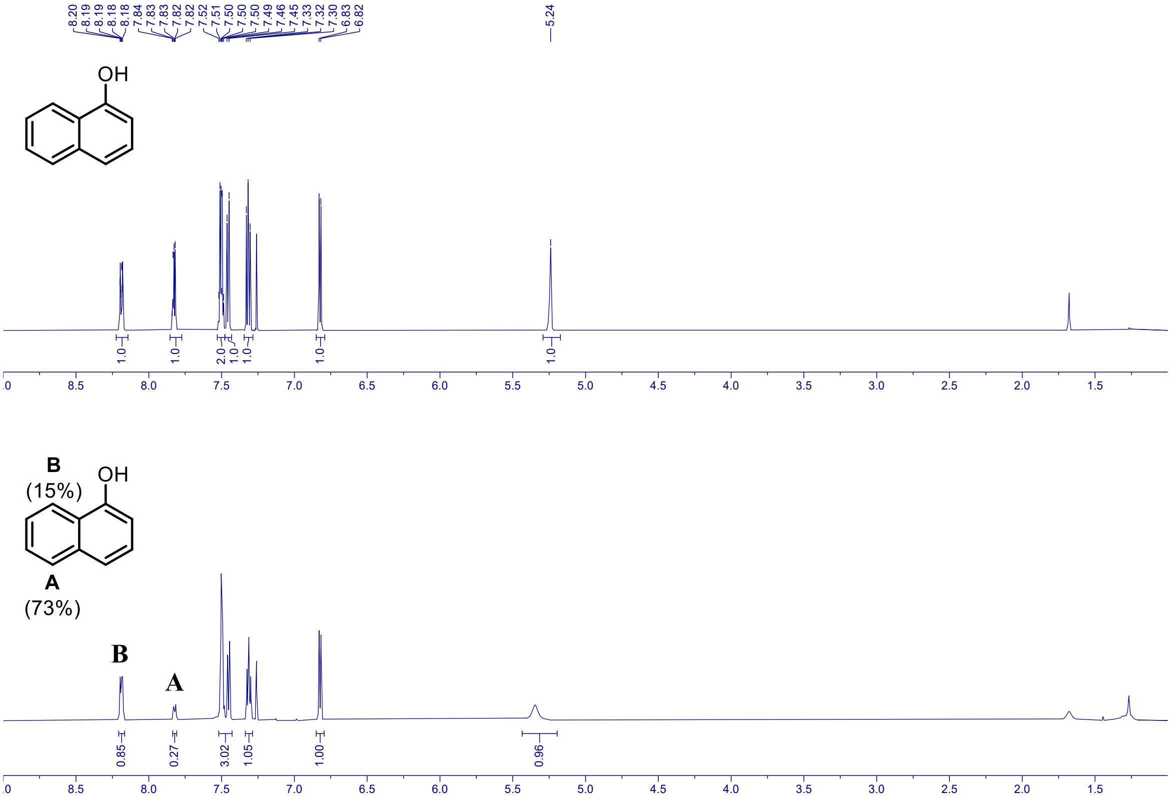

Figure S8. Stacked ^{1}H NMR (600 MHz, $CDCl_3$) spectra of **2**: natural abundance (top) and labelled (bottom).

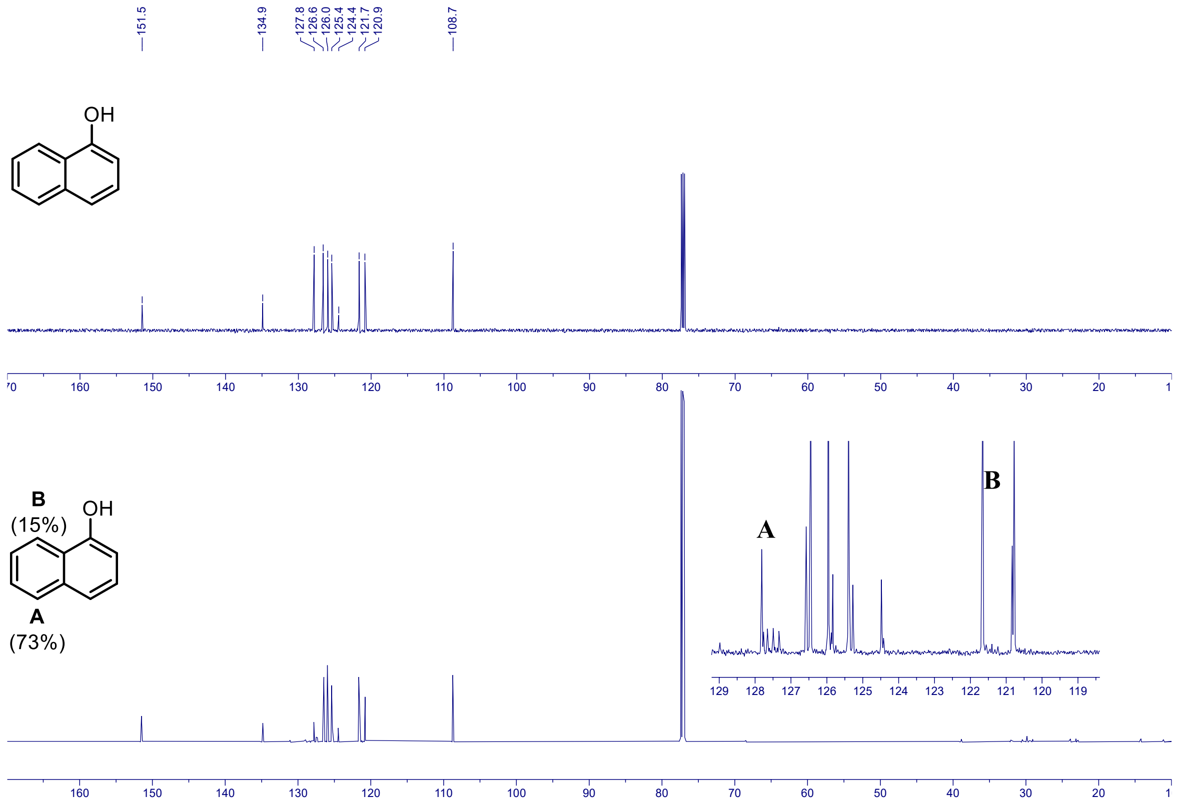

Figure S9. Stacked ^{13}C NMR (151 MHz, $CDCl_3$) spectra of **2**: natural abundance (top) and labelled (bottom).

[^{2}H]1-Naphthylamine (3)

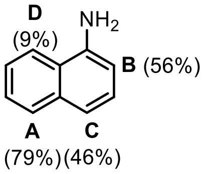

Obtained from 1-naphthylamine following procedure **GP2**. [370 nm]. (>95%, D_{Total} = 1.90); **^{1}H NMR (600 MHz, THF-d$_8$)** δ 7.94 (0.91H, d, *J*=8.0 Hz, labeled, 9% D), 7.71 (0.21H, d, *J*=7.8 Hz, labeled, 79% D), 7.41–7.26 (2H, m), 7.18 (1H, s), 7.12 (0.54H, d, *J*=8.1 Hz, labeled, 46% D), 6.67 (0.44H, d, *J*=7.4 Hz, labeled, 56% D), 5.05 (2H, s); **^{13}C NMR (151 MHz, THF-d$_8$)** δ 145.4, 135.9, 129.0–128.5 (m, labeled), 127.4, 126.1, 124.7, 124.6, 122.6, 117.5–117.0 (m, labeled), 109.0–108.6 (m, labeled); **HRMS** (EI) Calculated for $C_{10}H_9N$ $[M]^+$ 143.0730 found 143.0729 (4%); Calculated for $C_{10}H_8DN$ $[M]^+$ 144.0792 found 144.0793 (33%); Calculated for $C_{10}H_7D_2N$ $[M]^+$ 145.0855 found 145.0852 (48%); Calculated for $C_{10}H_6D_3N$ $[M]^+$ 146.0918 found 146.0916 (15%); D_{Total} = 1.74 calculated by HRMS.

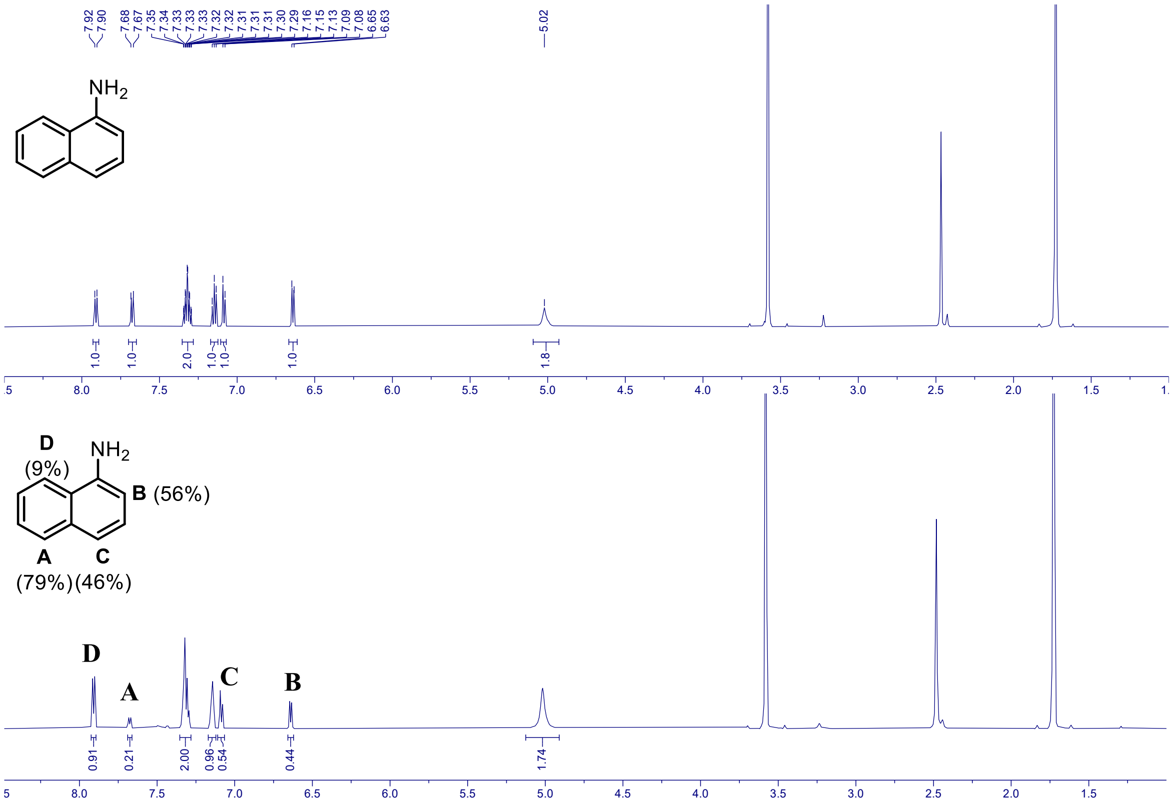

Figure S10. Stacked ^{1}H NMR (600 MHz, THF-*d$_8$*) spectra of **3**: natural abundance (top) and labelled (bottom).

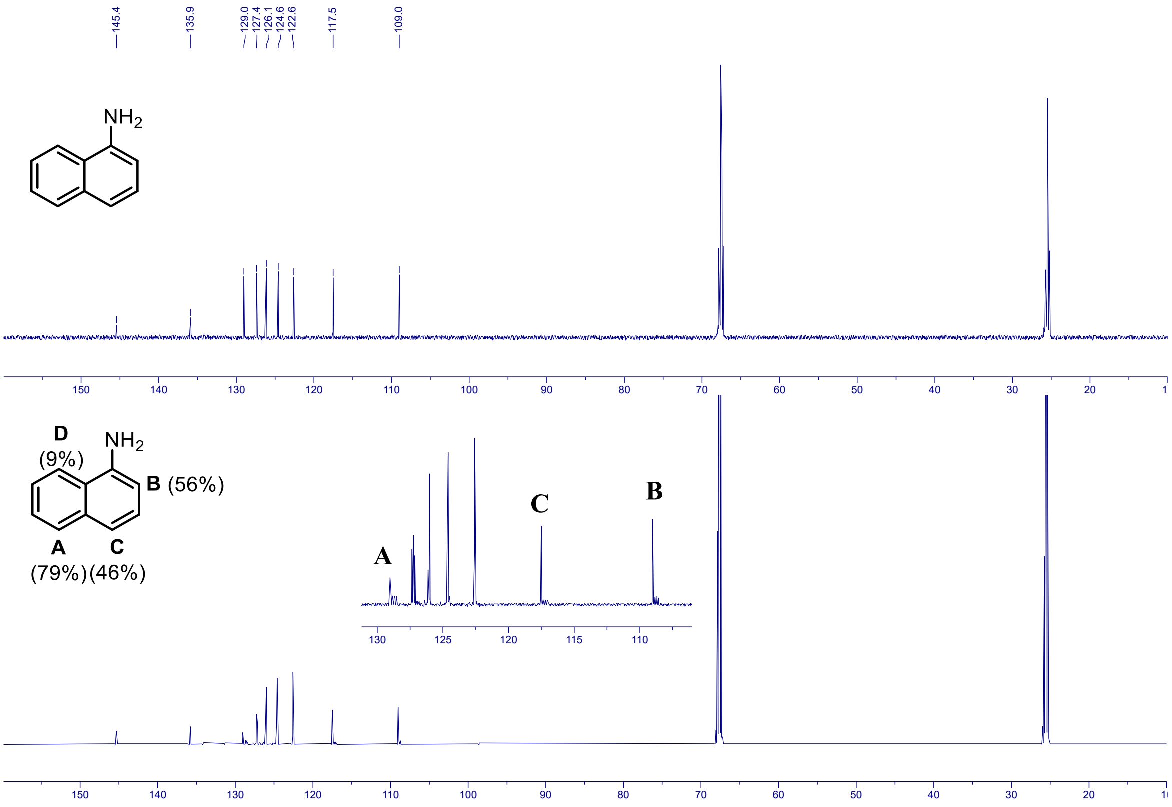

Figure S11. Stacked ^{13}C NMR (151 MHz, THF-d_8) spectra of **3**: natural abundance (top) and labelled (bottom).

[^{2}H]*tert*-Butyl ((4-methoxynaphthalen-1-yl)methyl)(1-methoxypropan-2-yl)carbamate (4)

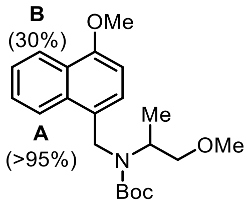

Obtained from *tert*-butyl ((4-methoxynaphthalen-1-yl)methyl)(1-methoxypropan-2-yl)carbamate following procedure **GP1** [300 nm]. (>95%, D_{Total} = 1.25); **^{1}H NMR (600 MHz, $CDCl_3$, rotamers)**: δ 8.30 (0.7H, d, *J*=8.2 Hz, labeled 30% D), 7.55–7.46 (2H, m), 7.29 (1H, d, *J*=7.8 Hz), 6.76 (1H, d, *J*=7.8 Hz), 4.98 (0.5H, bs), 4.81 (0.5H, bs) 4.79–4.72 (1H, m), 4.30 (0.5H, bs), 3.99 (3H, s), 3.84–3.70 (0.5H, m), 3.39–3.11 (5H, m), 1.58–1.47 (5H, m), 1.46–1.35 (4H, m), 1.11–0.96 (3H, m). Due to the presence of rotamers and the broadening of signals, the full characterization by ^{13}C NMR of this compound could not be possible (see below). Representative signals: **^{13}C NMR (151 MHz, $CDCl_3$, rotamers)**: δ 155.9 (bs), 126.5 (bs), 125.9 (bs), 125.1 (bs), 124.3(bs), 122.7 (bs), 103.1, 79.9 (bs), 75.5 (bs), 74.9 (bs), 58.8, 55.6, 51.9 (bs), 51.3 (bs), 47.9 (bs), 45.8 (bs), 28.6 (bs), 16.2 (bs), 15.4 (bs); **HRMS** (ESI) calculated for $C_{21}H_{28}DNO_4Na$ $[M+Na]^+$ 383.2057 found 383.2051 (73%); calculated for $C_{21}H_{27}D_2NO_4Na$ $[M+Na]^+$ 384.2120 found 384.2102 (27%); D_{Total} = 1.27 calculated by HRMS.

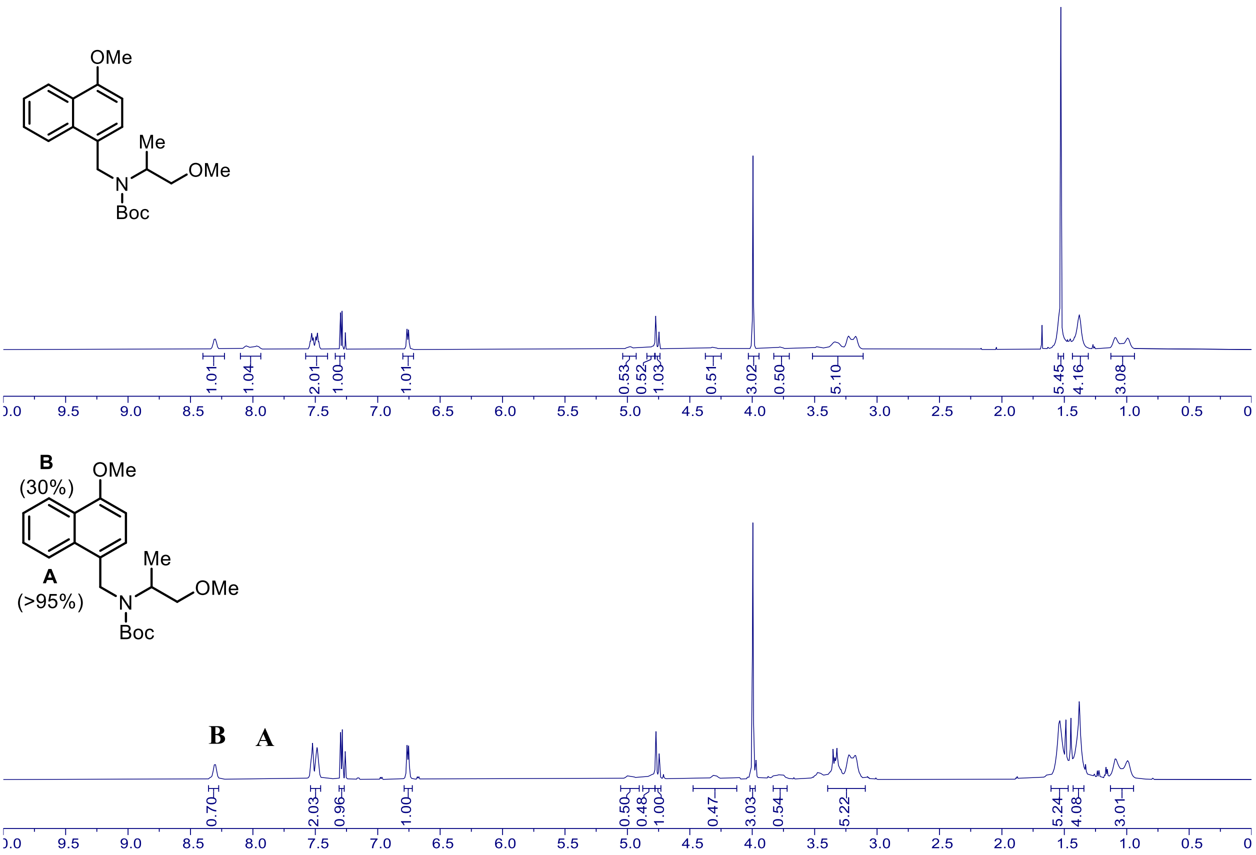

Figure S12. Stacked ^{1}H NMR (600 MHz, $CDCl_3$) spectra of **4**: natural abundance (top) and labelled (bottom).

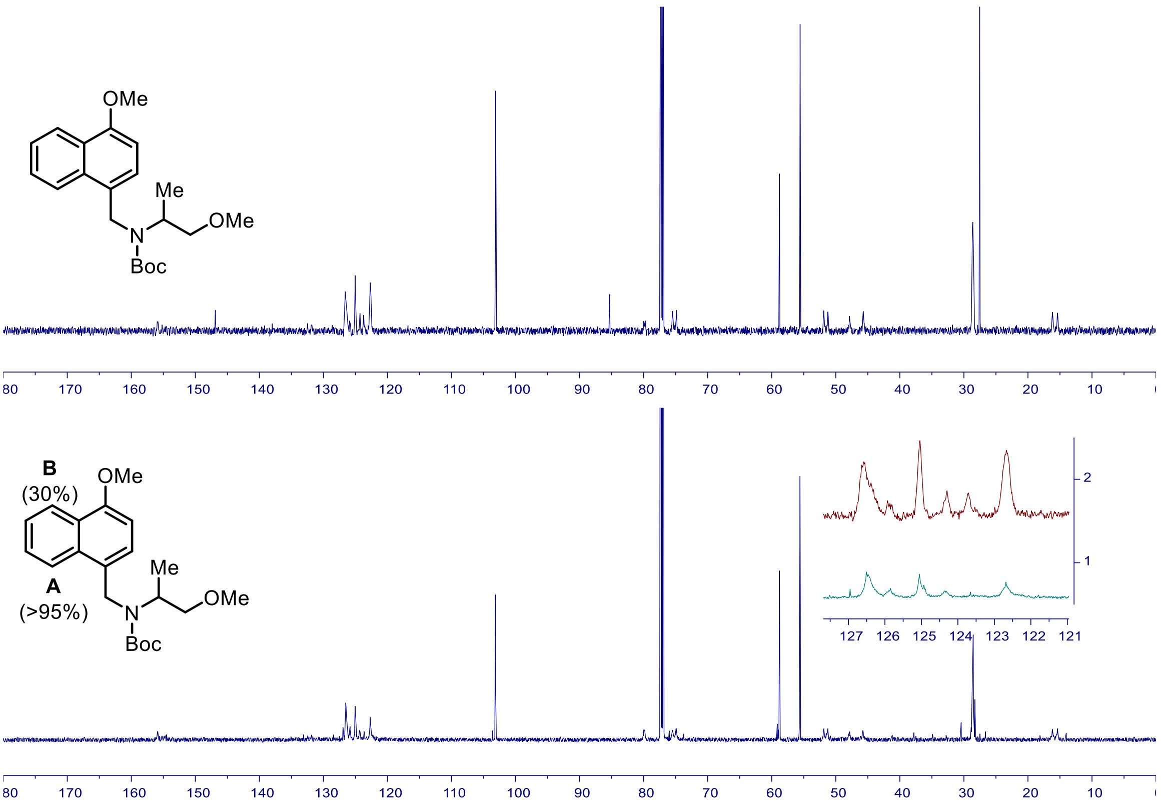

Figure S13. Stacked ^{13}C NMR (151 MHz, $CDCl_3$) spectra of **4**: natural abundance (top) and labelled (bottom).

[^{2}H]1-Ethylnaphthalene (5)

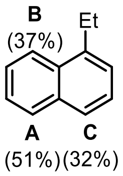

Obtained from 1-ethylnaphthalene following procedure **GP1** [254 nm]. (80%, D_{Total} = 1.20); **^{1}H NMR (600 MHz, CDCl$_3$)** δ 8.10 (0.63H, d, *J*=8.3 Hz, labeled, 37% D), 7.89 (0.49H, d, *J*=8.1 Hz, labeled, 51% D), 7.74 (0.68H, d, *J*=8.2 Hz, labeled, 32% D), 7.55–7.48 (2H, m), 7.44 (1H, t, *J*=7.6 Hz), 7.38 (1H, d, *J*=7.0 Hz), 3.16 (2H, q, *J*=7.5 Hz), 1.43 (3H, t, *J*=7.5 Hz); **^{13}C NMR (151 MHz, CDCl$_3$)** δ 140.4, 134.0, 132.0, 128.9–128.4 (m, labeled), 126.6, 125.9, 125.6, 125.0, 123.9–123.4 (m, labeled), 26.1, 15.2; **HRMS** (EI) Calculated for $C_{12}H_{12}$ $[M]^+$ 156.0934 found 156.0936 (30%); Calculated for $C_{12}H_{11}D$ $[M]^+$ 157.0996 found 157.1000 (47%); Calculated for $C_{12}H_{10}D_2$ $[M]^+$ 158.1059 found 158.1064 (20%); ($C_{12}H_9D_3$ 3%); D_{Total} = 0.96 calculated by HRMS.

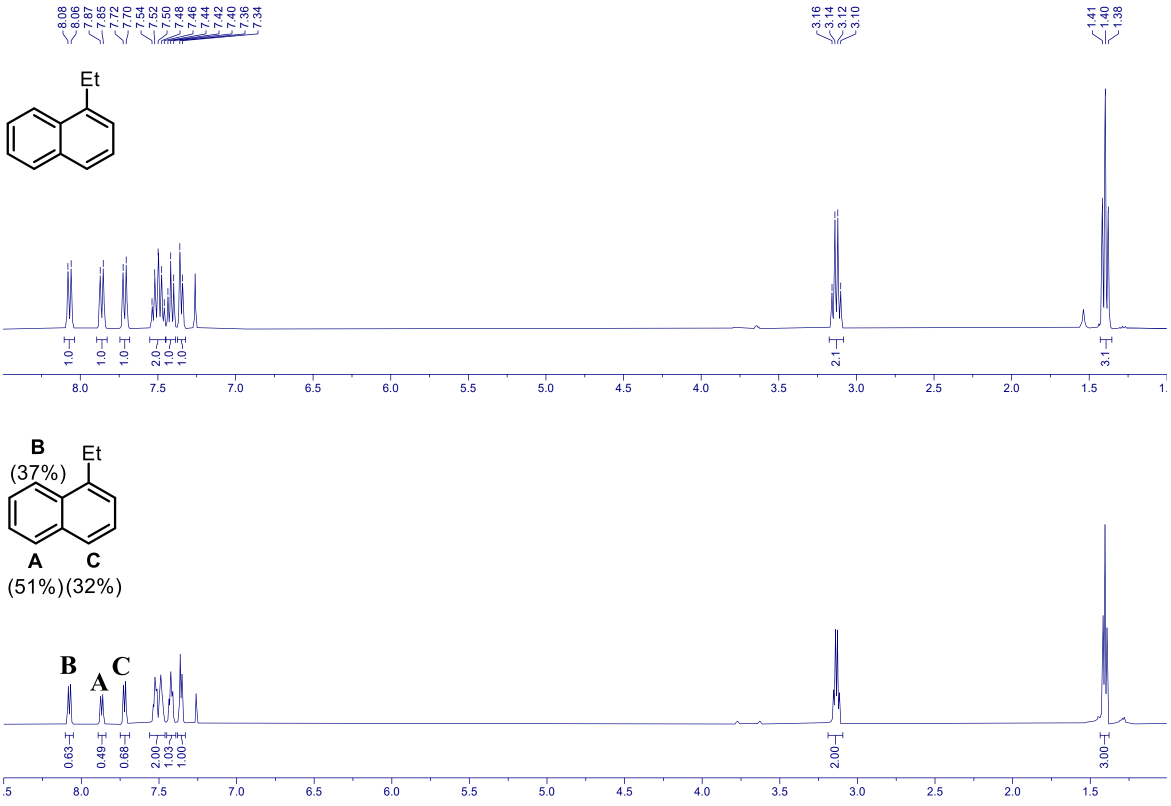

Figure S14. Stacked ^{1}H NMR (600 MHz, CDCl$_3$) spectra of **5**: natural abundance (top) and labelled (bottom).

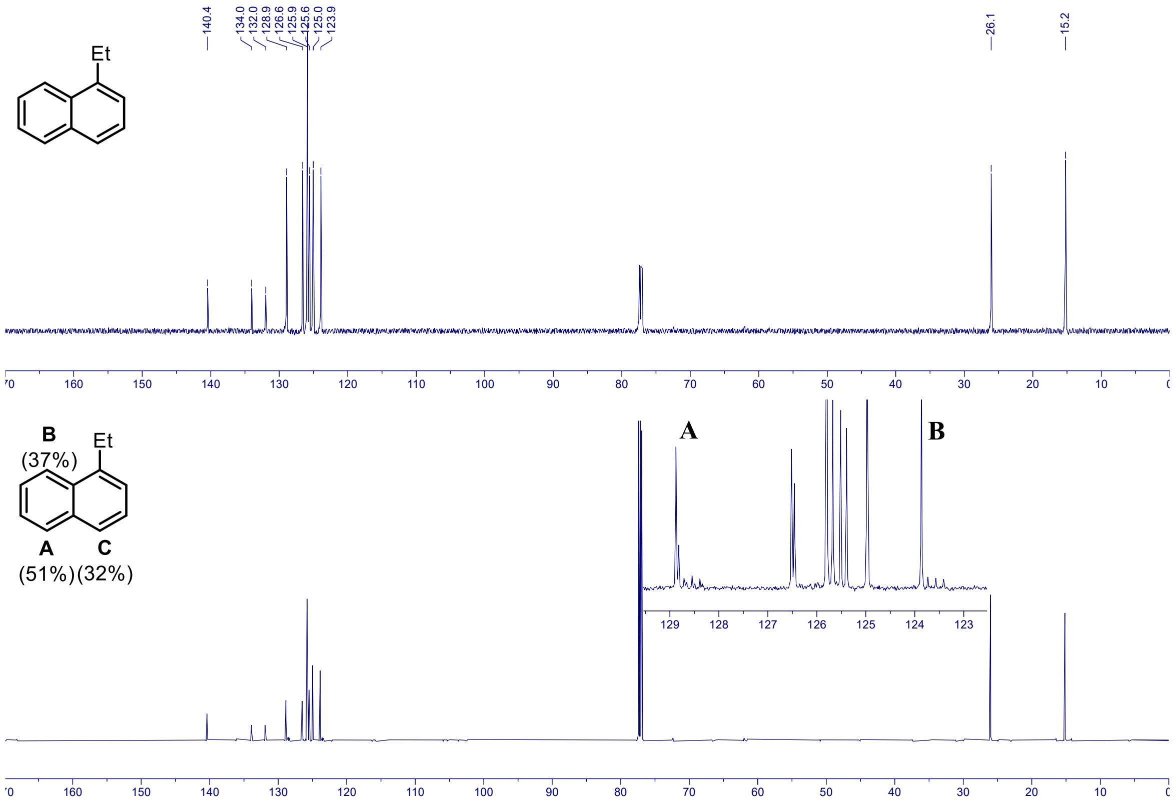

Figure S15. Stacked ^{13}C NMR (151 MHz, $CDCl_3$) spectra of **5**: natural abundance (top) and labelled (bottom).

[²H]2,6-Dimethylaniline (6)

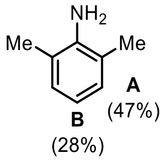

Obtained from 2,6-dimethylaniline following procedure **GP1**. [254 nm]. (70%, D_{Total} = 1.22); **^{1}H NMR (600 MHz, $CDCl_3$)** δ 6.94 (1.06H, d, *J*=7.5 Hz, labeled, 47%), 6.64 (0.72H, bs, labeled, 28%), 2.20 (6H, s); **^{13}C NMR (151 MHz, $CDCl_3$)** δ 142.8, 128.3–127.9 (m, labeled), 121.7, 118.3–118.0 (m, labelled), 17.7; **HRMS** (EI) Calculated for $C_8H_{11}N$ [M^+] 121.0886 found 121.0877 (35%); Calculated for $C_8H_{10}DN$ $[M]^+$ 122.0949 found 122.0944 (40%), Calculated for $C_8H_9D_2N$ $[M]^+$ 123.1012 found 123.1010 (20%); ($C_8H_8D_3N$ 5%); D_{Total} = 0.95 calculated by HRMS.

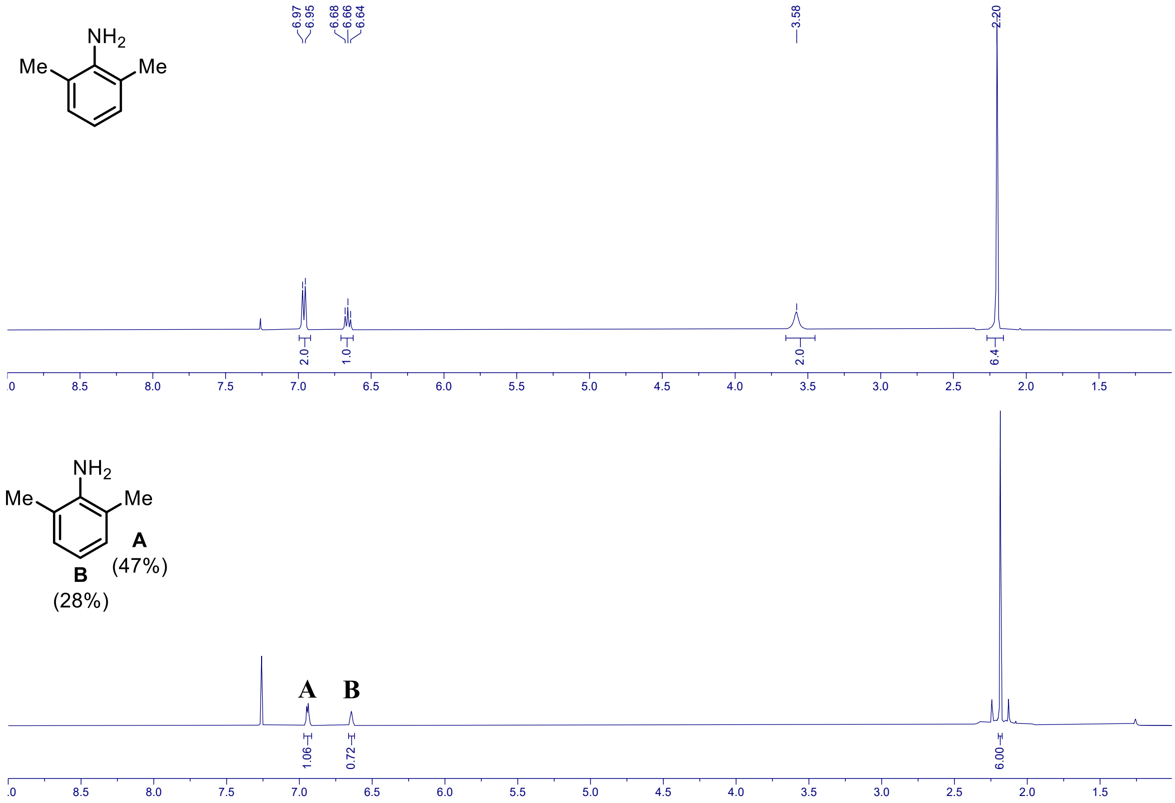

Figure S16. Stacked ^{1}H NMR (600 MHz, $CDCl_3$) spectra of **6**: natural abundance (top) and labelled (bottom).

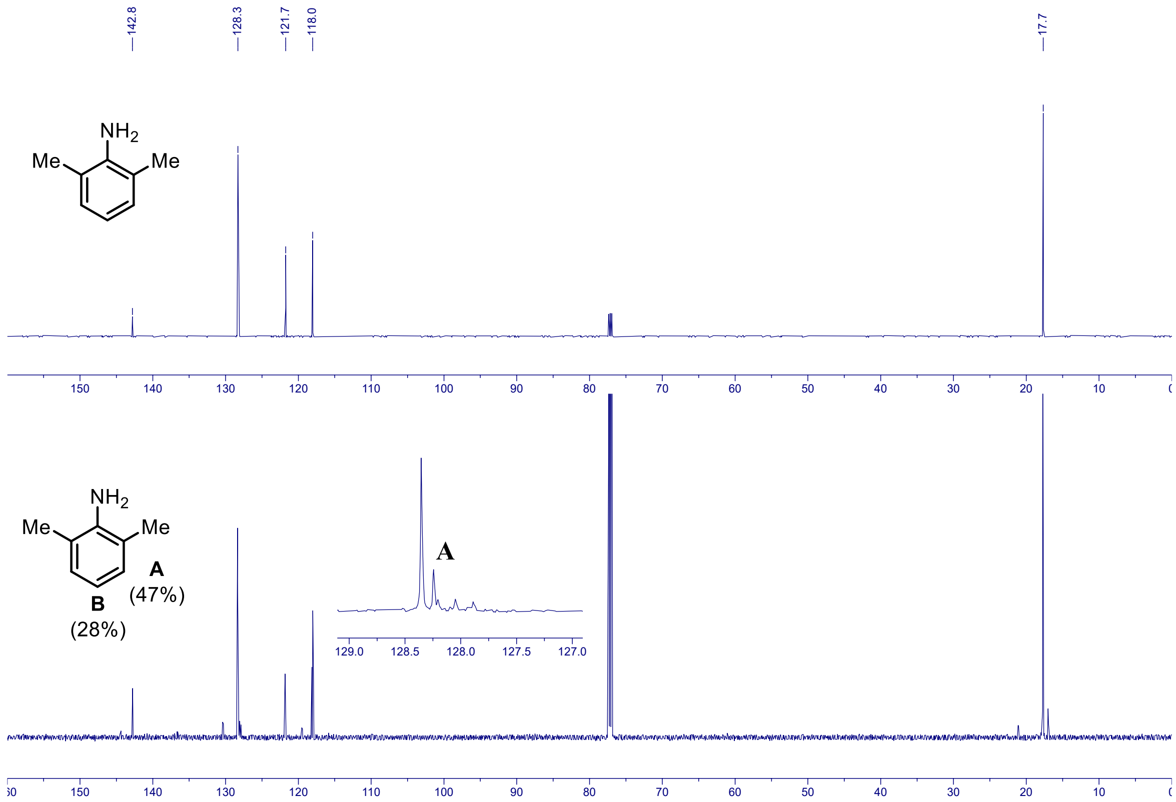

Figure S17. Stacked ^{13}C NMR (151 MHz, $CDCl_3$) spectra of **6**: natural abundance (top) and labelled (bottom).

[²H]*m*-Anisidine (7)

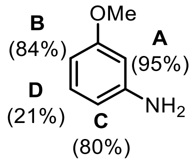

Obtained from *m*-anisidine following procedure **GP1**. [254 nm]. (>95%, D_{Total} = 2.80); **^{1}H NMR (600 MHz, $CDCl_3$)** δ 7.06 (0.79H, s, labeled, 21% D), 6.38–6.32 (0.16H, m, labeled, 84% D), 6.32–6.27 (0.2H, m, labeled, 80% D), 6.25 (0.05H, s, labeled, 85% D), 3.77 (3H, s), 3.66 (2H, s); **^{13}C NMR (151 MHz, $CDCl_3$)** δ 160.7, 147.9, 130.1, 107.9–107.6 (m, labeled), 103.9–103.6 (m, labeled), 101.0–100.8 (m, labeled), 55.0; **HRMS** (EI) Calculated for $C_7H_7D_2NO$ $[M]^+$ 125.0804 found 125.0805 (27%); Calculated for $C_7H_6D_3NO$ $[M]^+$ 126.0867 found 126.0869 (58%); Calculated for $C_7H_5D_4NO$ $[M]^+$ 127.0930 found 127.0932 (12%); (C_7H_8DNO 3%); D_{Total} = 2.76 calculated by HRMS.

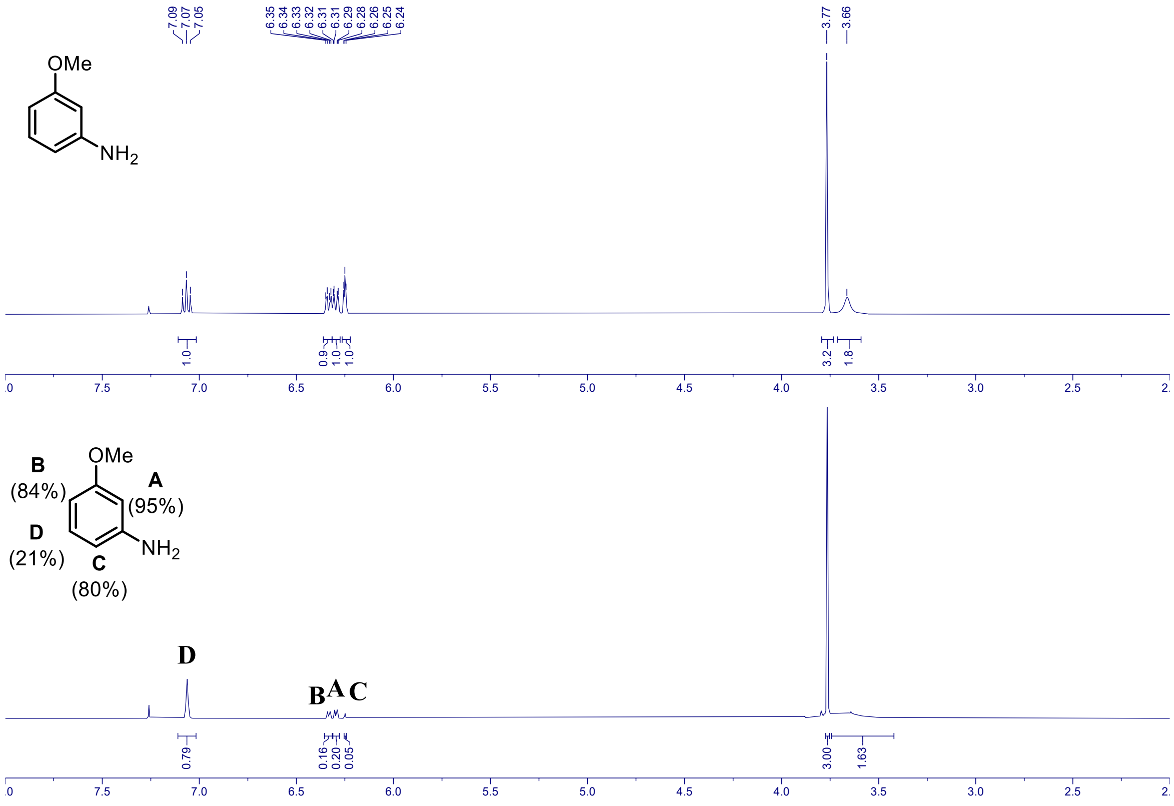

Figure S18. Stacked ^{1}H NMR (600 MHz, $CDCl_3$) spectra of **7**: natural abundance (top) and labelled (bottom).

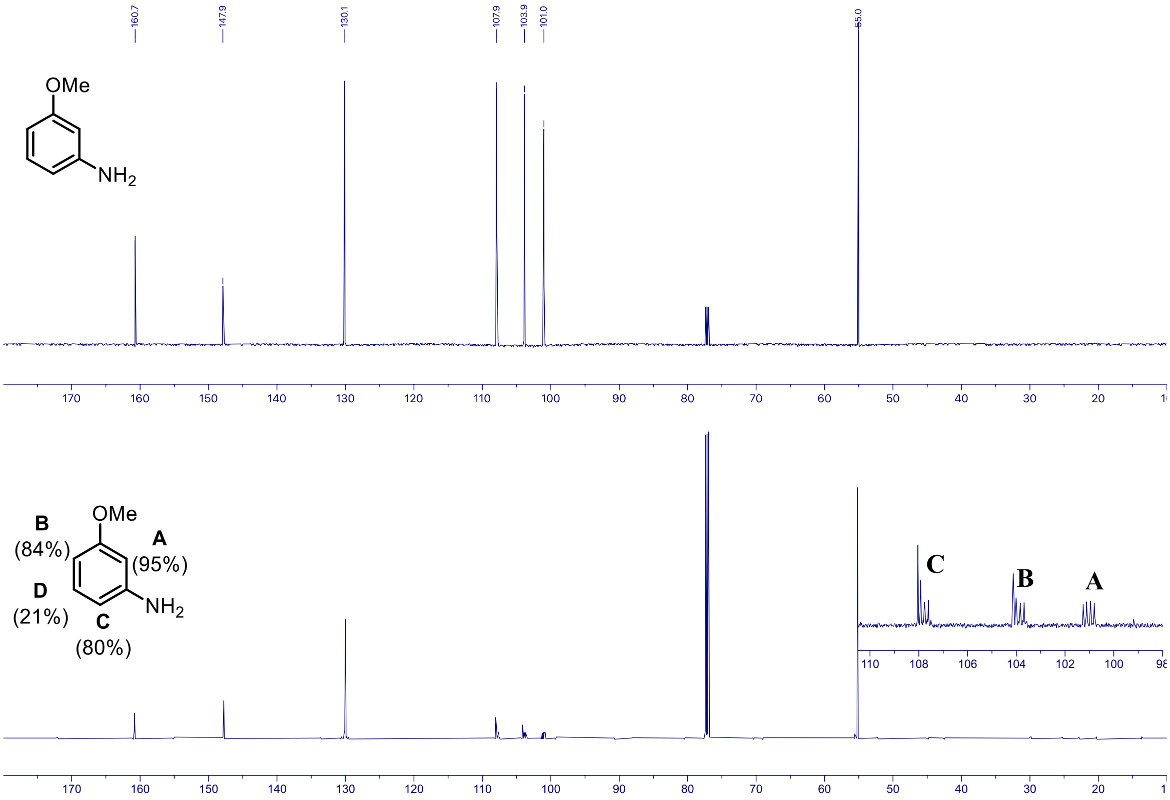

Figure S19. Stacked ^{13}C NMR (151 MHz, $CDCl_3$) spectra of **7**: natural abundance (top) and labelled (bottom).

[^{2}H]3-Methylindole (8)

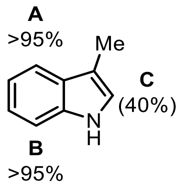

Obtained from 3-methyl indole following procedure **GP1**. [254 nm]. (>95%, D_{Total} = 2.30); **^{1}H NMR (600 MHz, $CDCl_3$)** δ 7.20 (1H, d, J=7.1 Hz), 7.13 (1H, d, J=7.1 Hz), 6.97 (0.6H, bs, labeled 40% D), 2.35 (3H, s); **^{13}C NMR (151 MHz, $CDCl_3$)** δ 136.3, 128.4, 122.0–121.8 (m, labeled), 121.7, 119.1, 118.7 (m, labeled), 111.8, 111.1 – 110.5 (m, labeled), 9.8; **HRMS** (EI) calculated for $C_9H_7D_2N$ $[M]^+$ 133.0860 found 133.0859 (62%); calculated for $C_9H_6D_3N$ $[M]^+$ 134.0923 found 134.0930 (38%); D_{Total} = 2.38 calculated by HRMS.

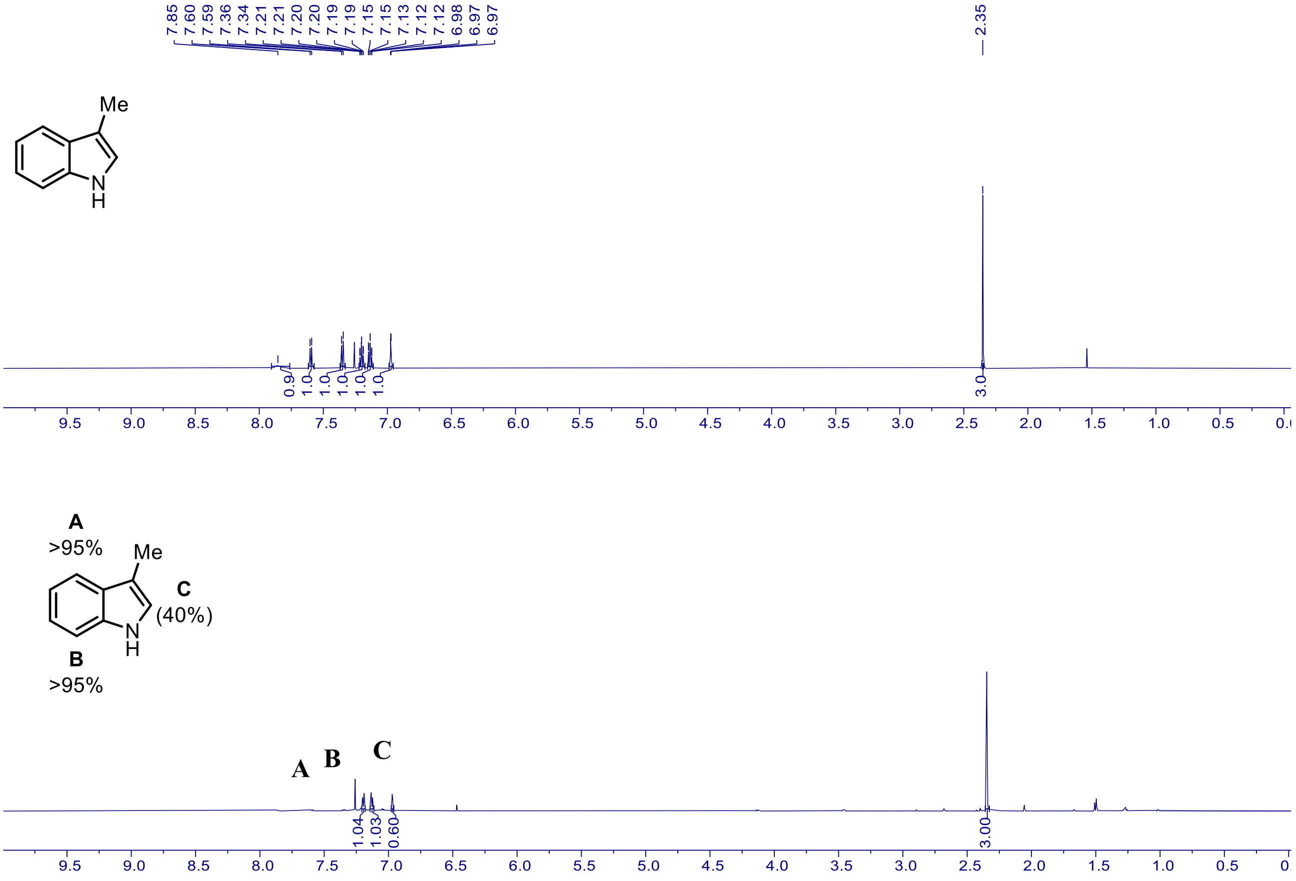

Figure S20. Stacked ^{1}H NMR (600 MHz, $CDCl_3$) spectra of **8**: natural abundance (top) and labelled (bottom).

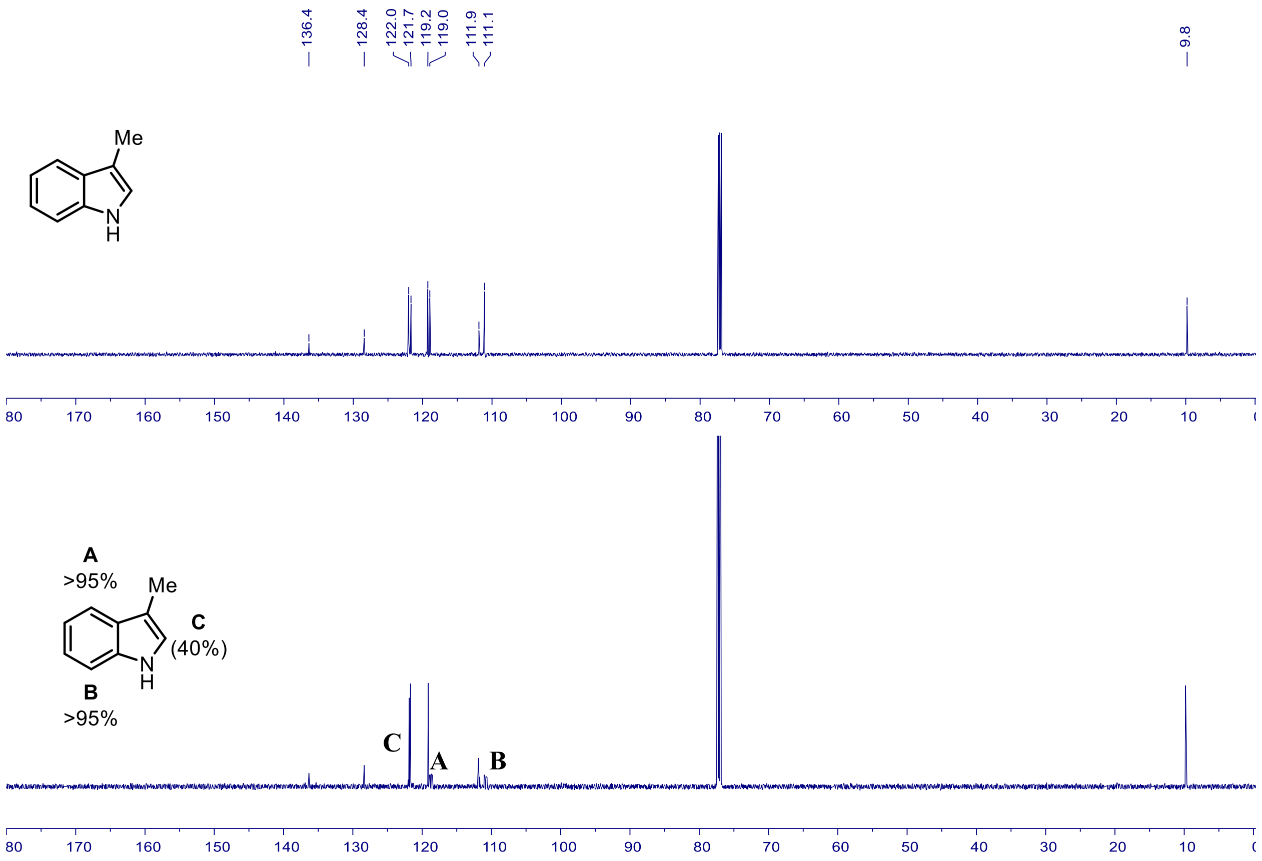

Figure S21. Stacked ^{13}C NMR (151 MHz, $CDCl_3$) spectra of **8**: natural abundance (top) and labelled (bottom).

[²H]Propanolol (9)

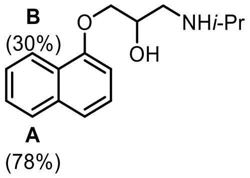

Obtained from propanolol following procedure **GP1** [300 nm]. (72%, D_{Total} = 1.08); **^{1}H NMR (400 MHz, $CDCl_3$)**: δ 8.09 (0.70H, dd, *J*=6.8, 2.9 Hz, <u>labeled 30 % D</u>), 7.65–7.60 (0.22H, m, <u>labeled 78% D</u>), 7.33–7.23 (2H, m), 7.17–7.08 (1H, m), 6.52 (1H, d, *J*=7.6 Hz), 4.68–4.57 (1H, m), 4.03 (1H, dd, *J*=9.8, 4.4 Hz), 3.96 (1H, dd, *J*=9.8, 5.8 Hz), 3.31 (1H, p, *J*=6.5 Hz), 3.25–3.09 (2H, m), 1.32 (3H, d, *J*=6.7 Hz), 1.31 (3H, d, *J*=6.7 Hz); **^{13}C NMR (101 MHz, $CDCl_3$)**: δ 153.9, 134.5, 127.6, 126.5, 125.9, 125.7–125.5 (<u>m, labeled</u>), 125.5–125.3 (<u>m, labeled</u>), 121.9, 121.0, 105.1, 69.8, 66.0, 51.7, 48.4, 19.2, 19.1; **HRMS** (ESI) calculated for $C_{16}H_{22}NO_2$ $[M+H]^+$ 260.1651 found 260.1646 (18%); calculated for $C_{16}H_{21}DNO_2$ $[M+H]^+$ 261.1713 found 261.1705 (62%); calculated for $C_{16}H_{20}D_2NO_2$ $[M+H]^+$ 262.1776 found 262.1756 (20%); D_{Total} = 1.02 calculated by HRMS.

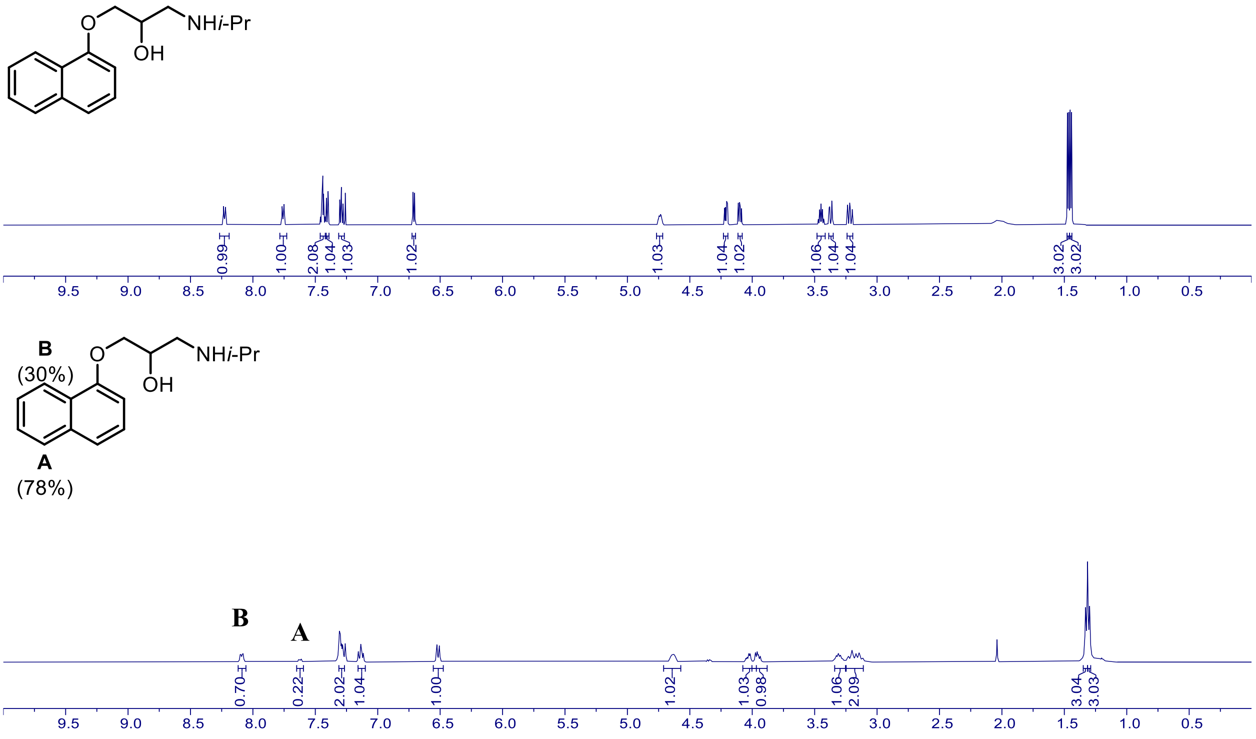

Figure S22. Stacked ^{1}H NMR (400 MHz, $CDCl_3$) spectra of **9**: natural abundance (top) and labelled (bottom).

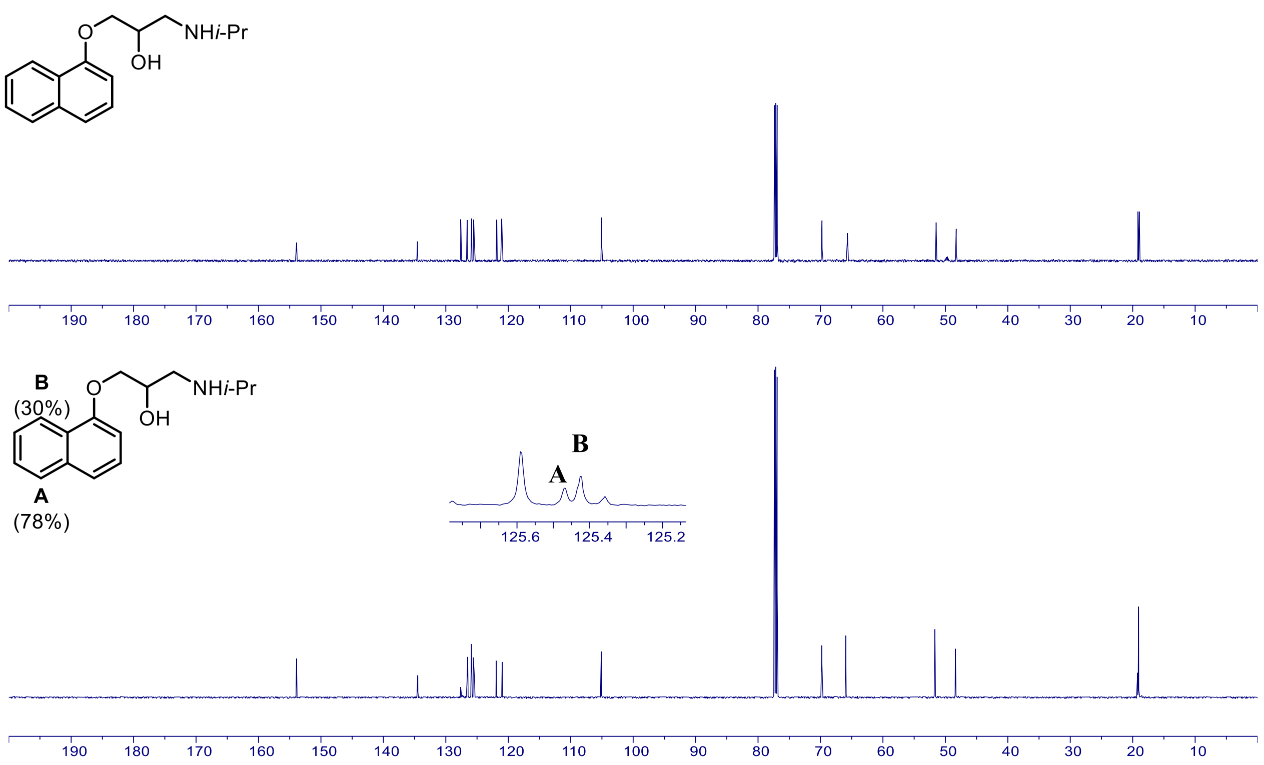

Figure S23. Stacked ^{13}C NMR (101 MHz, $CDCl_3$) spectra of **9**: natural abundance (top) and labelled (bottom).

[^{2}H]Dapoxetine (10)

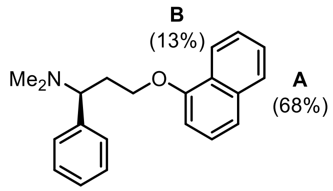

Obtained from dapoxetine following procedure **GP1**. [300 nm]. (>95%, D_{Total} = 0.81); **^{1}H NMR (600 MHz, CD_2Cl_2)** δ 8.06 (0.87H, d, *J*=8.2 Hz, labeled, 13% D), 7.79 (0.32H, d, *J*=8.0 Hz, labeled, 68% D), 7.58 (2H, m), 7.52–7.43 (5H, m), 7.41 (1H, d, *J*=8.3 Hz), 7.28 (1H, t, *J*=8.0 Hz), 6.61 (1H, d, *J*=7.6 Hz), 4.43–4.35 (1H, m), 4.20 (1H, dt, *J*=9.7, 4.7 Hz), 3.78 (1H, td, *J*=9.6, 3.8 Hz), 3.10 (1H, ddd, *J*=14.0, 9.4, 4.8 Hz), 2.97–2.74 (4H, m), 2.61 (3H, bs); **^{13}C NMR (151 MHz, CD_2Cl_2)** δ 154.3, 134.8, 130.5, 130.1, 129.9, 127.9–127.3 (m, labeled), 126.9–126.5 (m, labeled), 126.2, 125.8, 122.0, 120.9, 105.1, 69.7, 64.7, 31.3; **HRMS** (ESI) Calculated for $C_{21}H_{24}NO$ $[M+H]^+$ 306.1858 found 306.184718 (28%); Calculated for $C_{21}H_{23}DNO$ $[M+H]^+$ 307.1921 found 307.1905 (62%); Calculated for $C_{21}H_{22}D_2NO$ $[M+H]^+$ 308.1983 found 308.1952 (10%); D_{Total} = 0.82 calculated by HRMS.

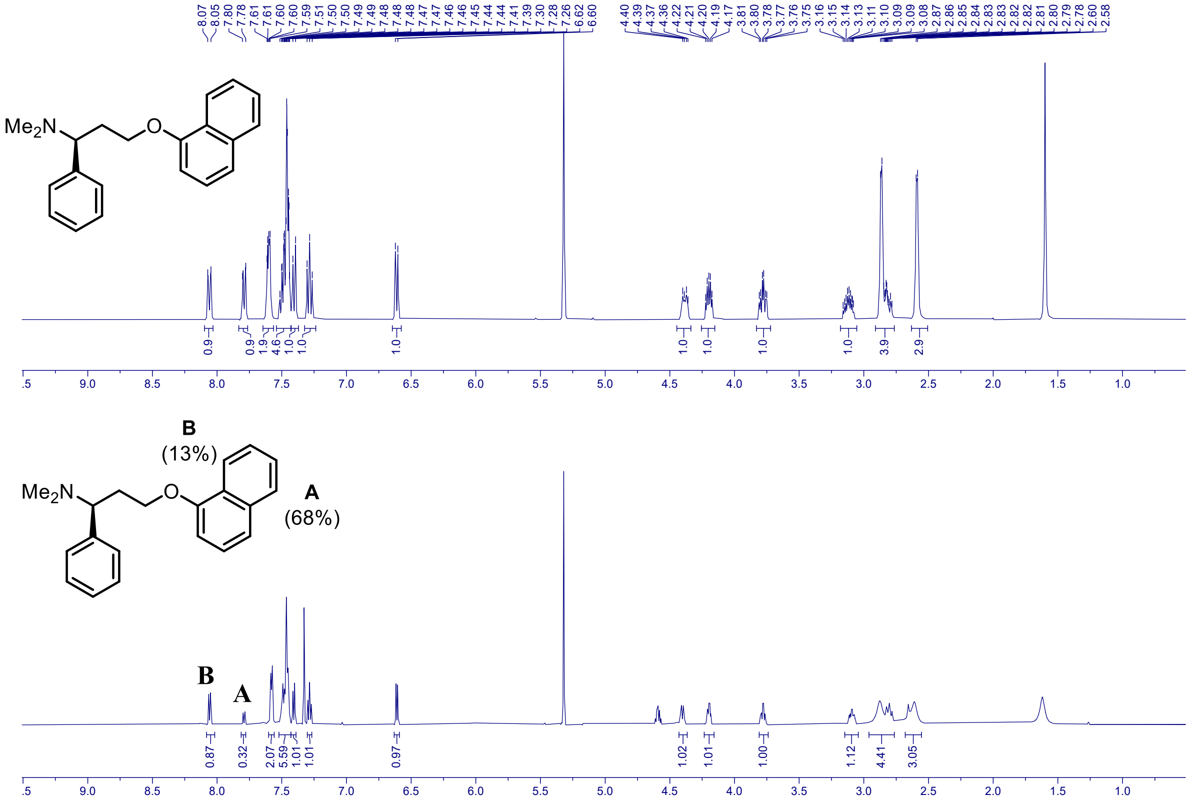

Figure S24. Stacked ^{1}H NMR (600 MHz, CD_2Cl_2) spectra of **10**: natural abundance (top) and labelled (bottom).

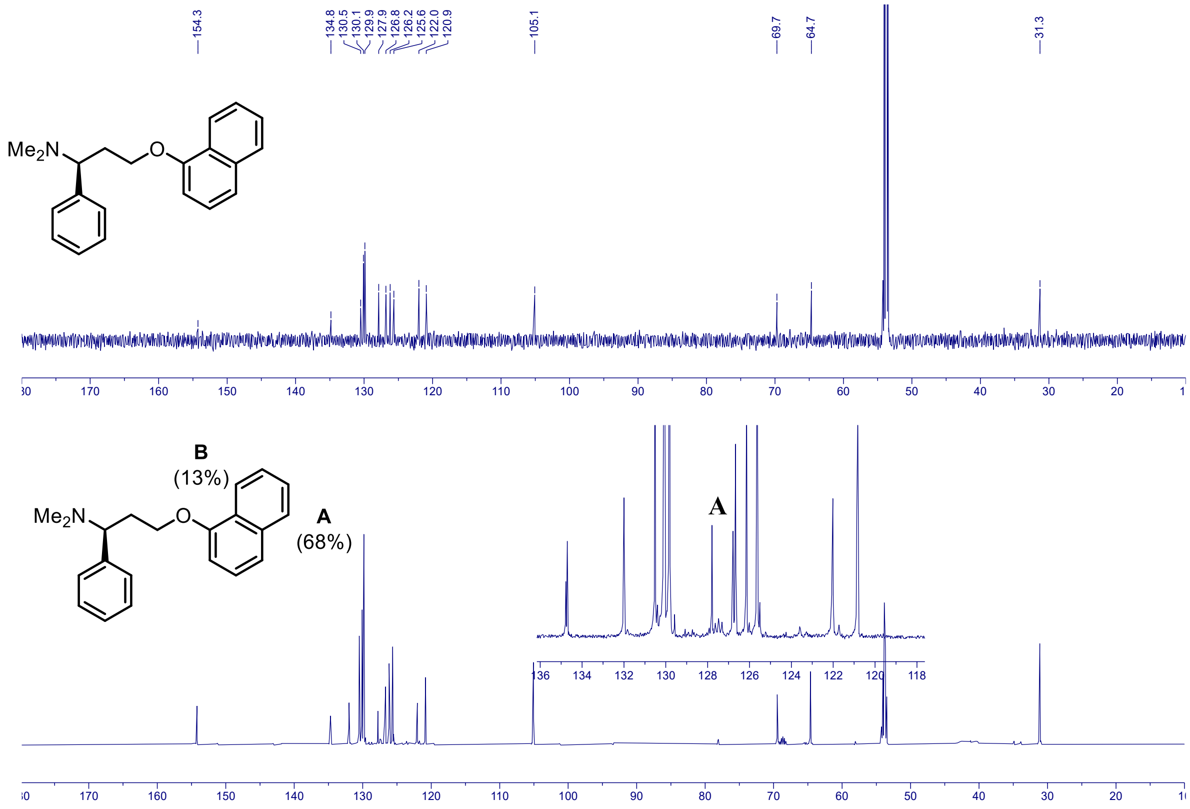

Figure S25. Stacked ^{13}C NMR (151 MHz, CD_2Cl_2) spectra of **10**: natural abundance (top) and labelled (bottom).

[²H]Naftopidil (11)

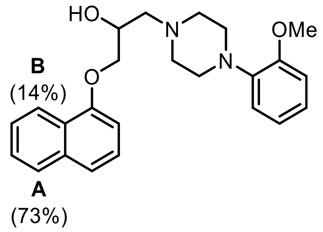

Obtained from naftopidil following procedure **GP1** [300 nm]. (>95%, D_{Total} = 0.87); **^{1}H NMR (600 MHz, CD_2Cl_2)** δ 8.27 (0.79H, d, *J*=7.8 Hz, labeled, 21% D), 7.81 (0.33H, d, *J*=7.7 Hz, labeled, 67% D), 7.55–7.47 (2H, m), 7.45 (1H, d, *J*=8.2 Hz), 7.39 (1H, t, *J*=7.9 Hz), 6.99 (1H, t, *J*=7.7 Hz), 6.96–6.89 (2H, m), 6.88 (2H, d, *J*=7.9 Hz), 4.35–4.25 (1H, m), 4.23–4.14 (2H, m), 3.84 (3H, s), 3.11 (4H, bs), 2.89 (2H, bs), 2.79–2.59 (4H, m); **^{13}C NMR (151 MHz, CD_2Cl_2)** δ 154.9, 152.8, 141.9, 134.9, 127.8–127.4 (m, labeled), 126.8, 126.3, 126.0, 125.6, 123.1, 122.2–121.8 (m, labeled), 121.3, 120.7, 118.5, 111.9, 105.3, 71.2, 66.0, 61.2, 55.7, 51.1; **HRMS** (ESI) Calculated for $C_{24}H_{29}N_2O_3$ $[M+H]^+$ 144.0570 found 144.0569 (43%); Calculated for $C_{24}H_{28}DN_2O_3$ $[M+H]^+$ 145.0632 found 145.0631 (49%); Calculated for $C_{24}H_{27}D_2N_2O_3$ $[M+H]^+$ 146.0695 found 146.0695 (8%); D_{Total} = 0.65 calculated by HRMS.

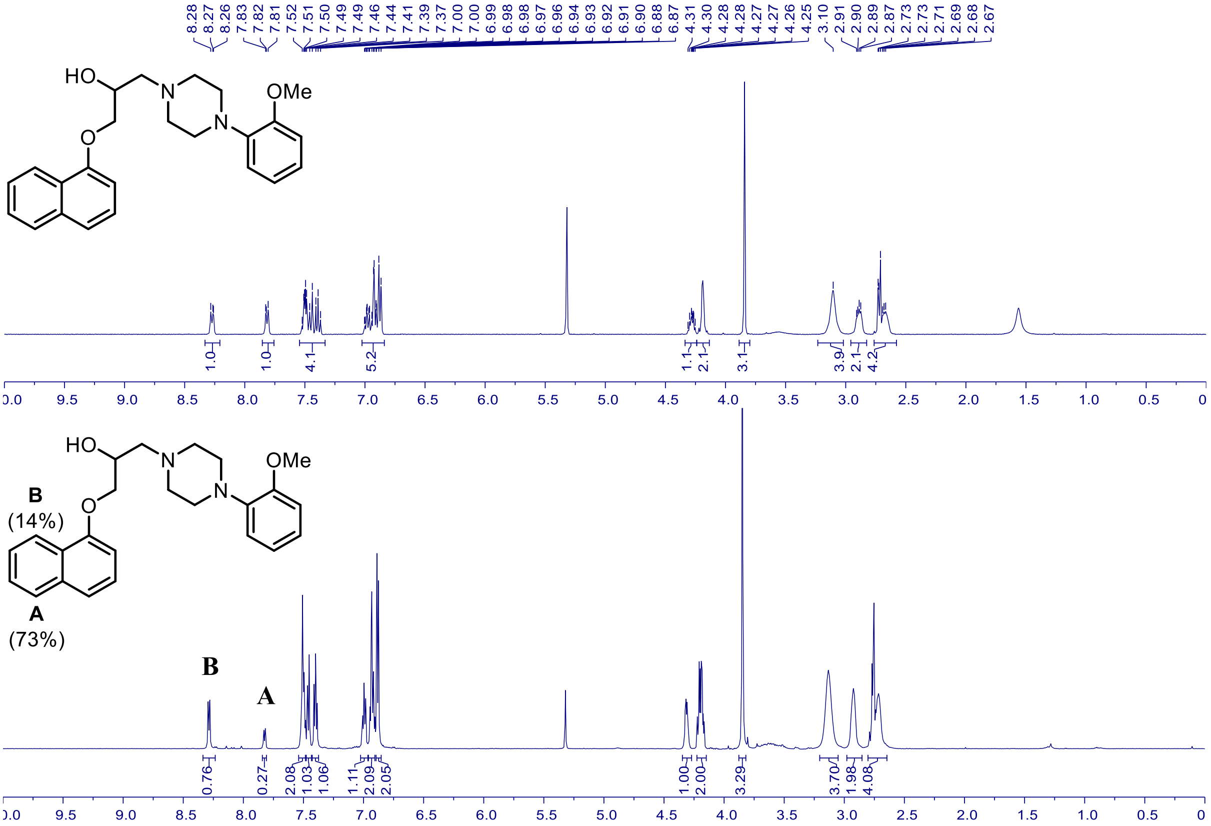

Figure S26. Stacked ^{1}H NMR (600 MHz, CD_2Cl_2) spectra of **11**: natural abundance (top) and labelled (bottom).

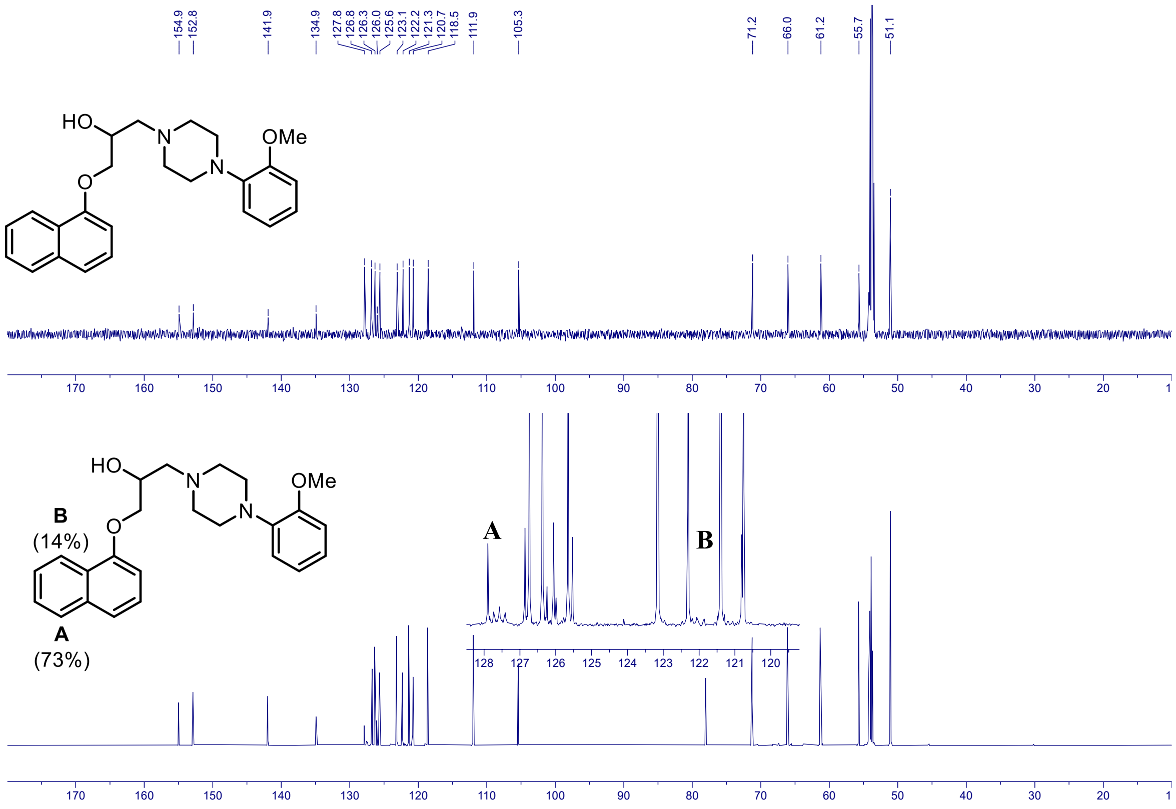

Figure S27. Stacked ^{13}C NMR (151 MHz, CD_2Cl_2) spectra of **11**: natural abundance (top) and labelled (bottom).

[^{2}H]Mephenesin (12)

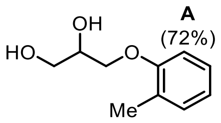

Obtained from mephenesin following procedure **GP1** [254 nm] using 1 mL of HFIP-d_1. Purification by prep TLC. (50%, D_{Total} = 0.72); **^{1}H NMR (600 MHz, CD_3OD)** δ 7.16–7.06 (2H, m), 6.88 (0.28H, d, *J*=8.0 Hz, labeled, 72% D), 6.81 (1H, t, *J*=7.4 Hz), 4.08–3.93 (3H, m), 3.81–3.70 (1H, m), 3.70–3.62 (1H, m), 2.22 (3H, s); **^{13}C NMR (151 MHz, CD_3OD)** δ 158.3, 131.5, 127.9, 127.8, 121.6, 112.2–111.8 (m, labeled), 71.9, 70.2, 64.3, 16.3; **HRMS** (EI) Calculated for Calculated for $C_{10}H_{14}O_3$ $[M]^+$ 182.0938 found 182.0938 (26%); Calculated for $C_{10}H_{13}DO_3$ $[M]^+$ 183.1000 found 183.1001 (64%); ($C_{10}H_{12}D_2O_3$ 11%); D_{Total} = 0.86 calculated by HRMS.

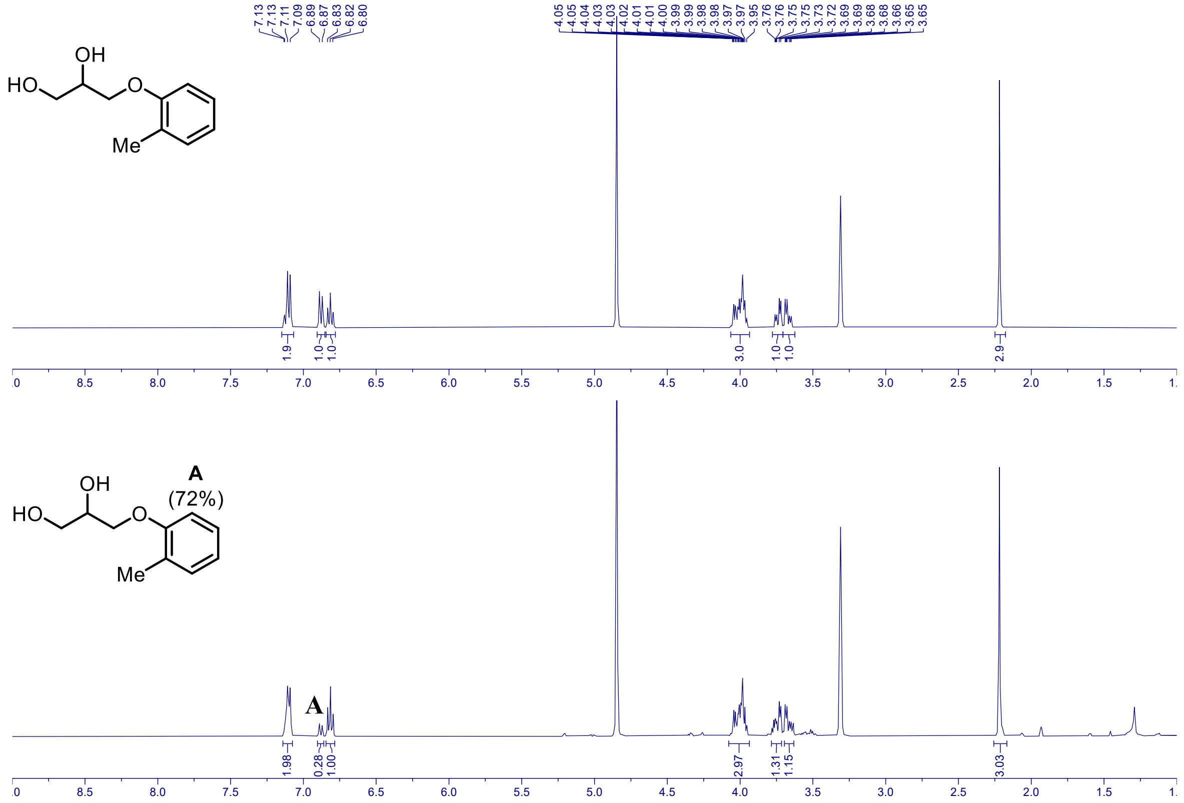

Figure S28. Stacked ^{1}H NMR (600 MHz, CD_3OD) spectra of **12**: natural abundance (top) and labelled (bottom).

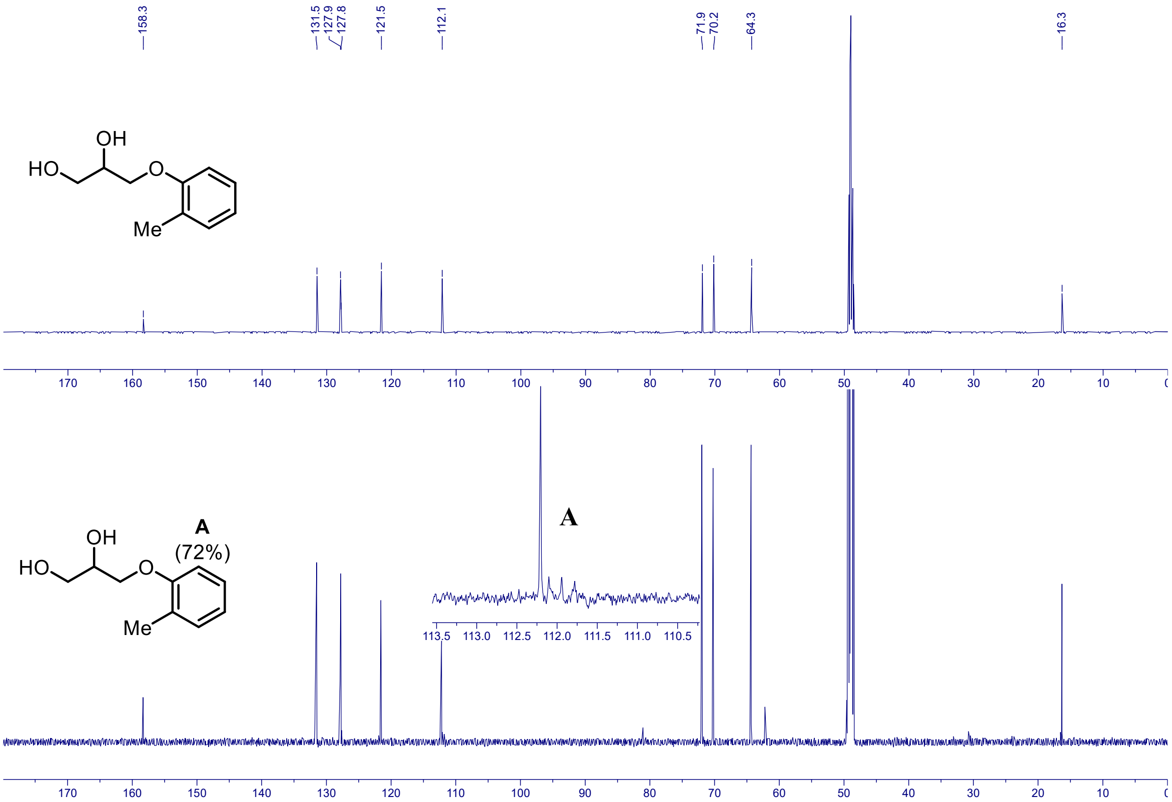

Figure S29. Stacked ^{13}C NMR (151 MHz, CD_3OD) spectra of **12**: natural abundance (top) and labelled (bottom).

[^{2}H]Methocarbamol (13)

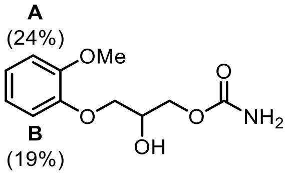

Obtained from methocarbamol following procedure **GP1** [254 nm]. (70%, D_{Total} = 0.43); **^{1}H NMR (400 MHz, acetone-*d_6*)** δ 6.99 (0.76H, d, *J*=7.8 Hz, labeled, 24% D), 6.96 (0.81H, d, *J*=8.1 Hz, labeled, 19% D), 6.92 (1H, t, *J*=7.7 Hz), 6.87 (1H, t, *J*=7.6 Hz), 5.89 (2H, bs), 4.31 (1H, bs), 4.22–4.16 (1H, m), 4.16–4.09 (2H, m), 4.05–3.96 (2H, m) 3.81 (3H, s); **^{13}C NMR (151 MHz, acetone-*d_6*)** δ 157.8, 150.9, 149.6, 122.4, 121.7, 115.4–114.6 (m, labeled), 113.4–112.6 (m, labeled), 71.5, 69.2, 66.4, 56.2; **HRMS** (ESI) Calculated for $C_{11}H_{15}NO_5Na$ $[M+Na]^+$ 264.0848 found 264.0842 (59%); Calculated for $C_{11}H_{14}DNO_5Na$ $[M+Na]^+$ 265.0911 found 265.0899 (39%); Calculated for $C_{11}H_{13}D_2NO_5Na$ $[M+Na]^+$ 266.0974 found 266.0963 (2%); D_{Total} = 0.43 calculated by HRMS.

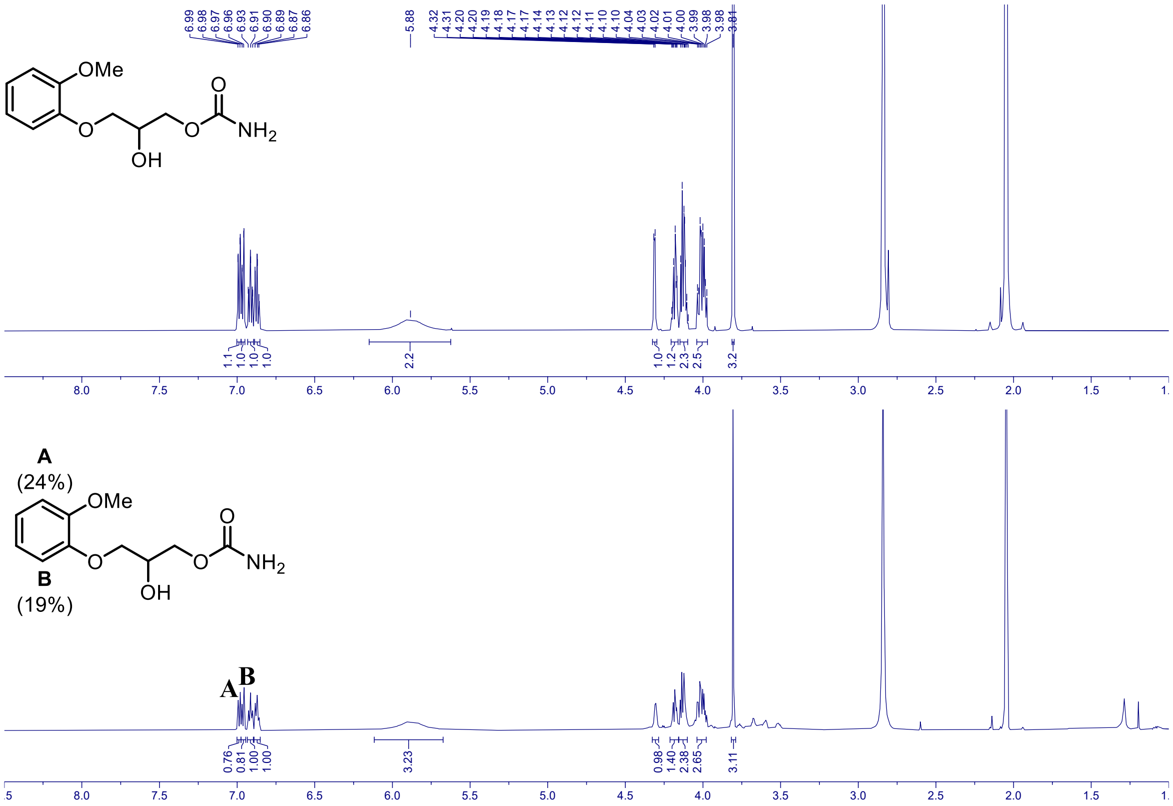

Figure S30. Stacked ^{1}H NMR (400 MHz, acetone-*d_6*) spectra of **13**: natural abundance (top) and labelled (bottom).

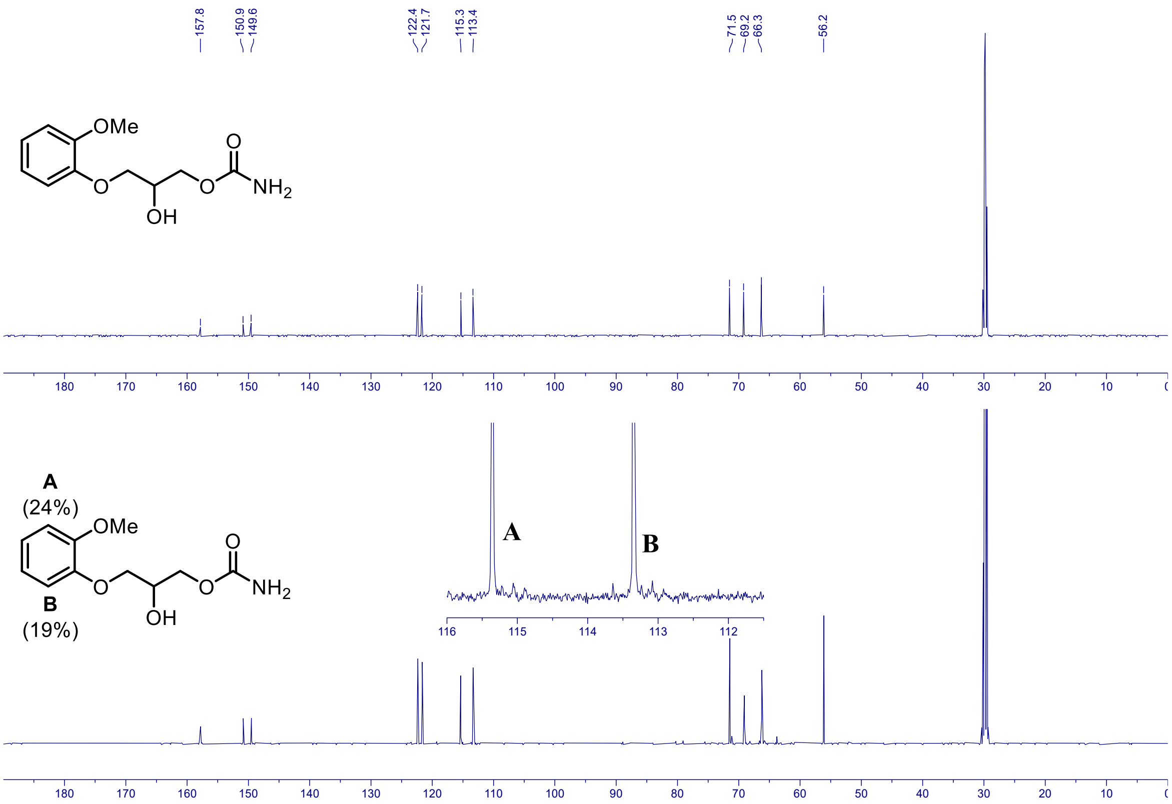

Figure S31. Stacked ^{13}C NMR (101 MHz, acetone-d_6) spectra of **13**: natural abundance (top) and labelled (bottom).

[^{2}H](+)-Rolipram (14)

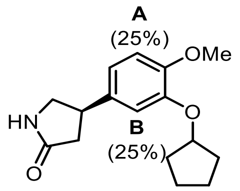

Obtained from (+)-rolipram following procedure **GP1** [254 nm]. (>95%, D_{Total} = 0.50); **^{1}H NMR (600 MHz, $CDCl_3$)**: δ 6.82 (1H, dd, *J*=8.1, 2.5 Hz), 6.77–6.74 (1.5H, m, <u>labeled 25% D</u>), 6.51 (1H, bs), 4.79–4.73 (1H, m), 3.82 (3H, s), 3.75 (1H, t, *J*=9.1 Hz), 3.61 (1H, p, *J*=8.4 Hz), 3.38 (1H, t, *J*=8.5 Hz), 2.70 (1H, dd, *J*=16.8, 9.0 Hz), 2.47 (1H, dd, *J*=16.9, 9.1 Hz), 1.94–1.78 (6H, m), 1.65–1.55 (2H, m); **^{13}C NMR (151 MHz, $CDCl_3$)**: δ 178.1, 149.3, 148.0, 134.6, 118.9, 114.1–113.6 (<u>m, labeled</u>), 112.6–112.2 (<u>m, labeled</u>), 80.7, 56.3, 49.9, 40.1, 38.2, 32.9, 24.1; **HRMS** (ESI) calculated for $C_{16}H_{21}NO_3Na$ $[M+Na]^+$ 298.1419 found 298.1412 (71%); calculated for $C_{16}H_{20}DNO_3Na$ $[M+Na]^+$ 299.1482 found 299.14641 (27%); calculated for $C_{16}H_{19}D_2NO_3Na$ $[M+Na]^+$ 300.1545 found 300.1506 (2%); D_{Total} = 0.31 calculated by HRMS.

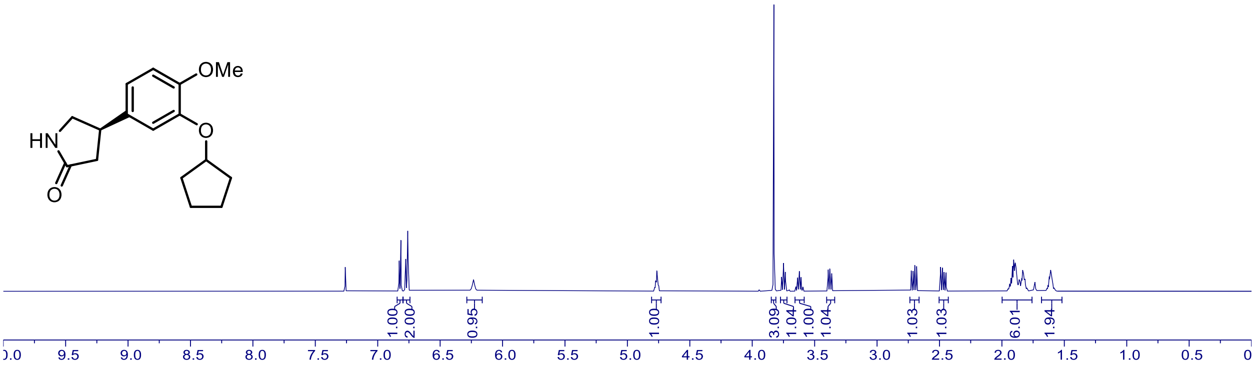

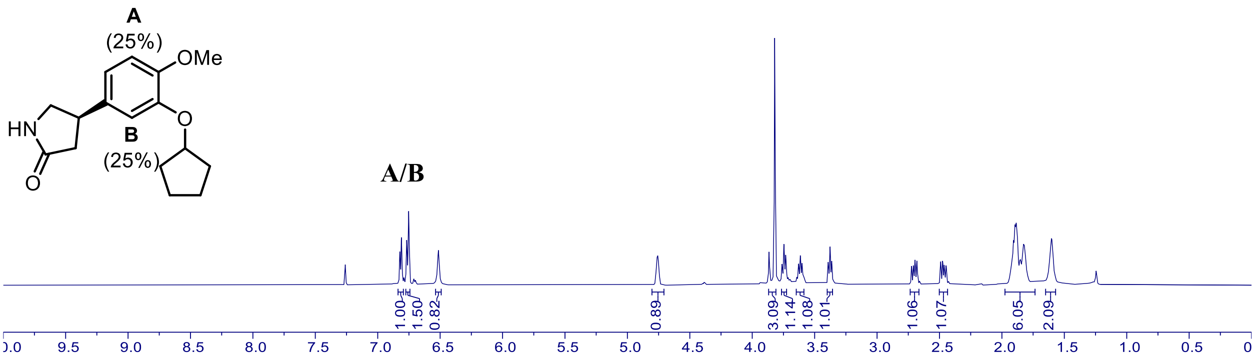

Figure S32. Stacked ^{1}H NMR (600 MHz, $CDCl_3$) spectra of **14**: natural abundance (top) and labelled (bottom).

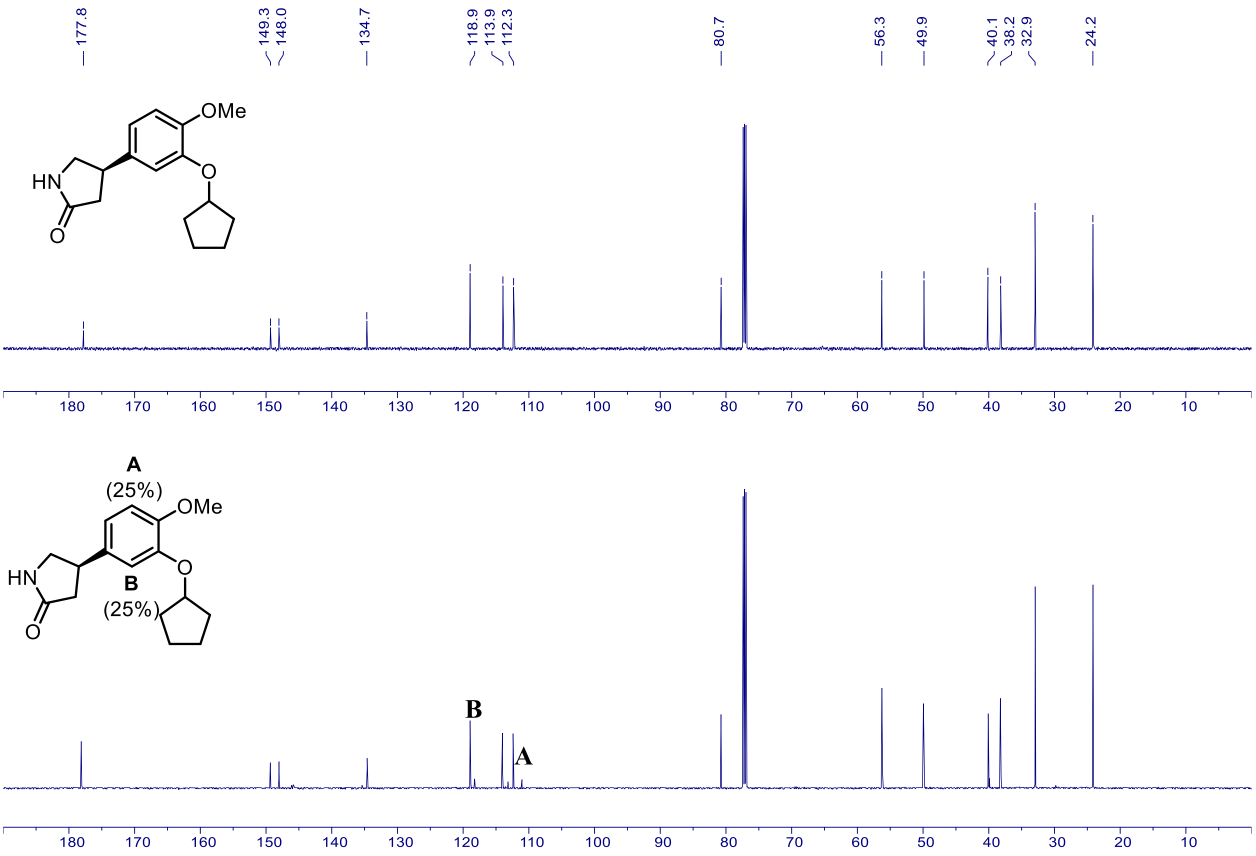

Figure S33. Stacked ^{13}C NMR (151 MHz, $CDCl_3$) spectra of **14**: natural abundance (top) and labelled (bottom).

[^{2}H]Empagliflozin (15)

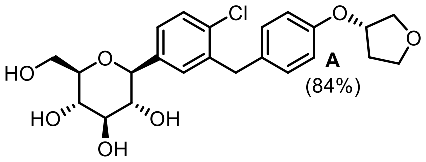

Obtained from empagliflozin following procedure **GP1** [254 nm]. Purification by prep TLC. (50%, D_{Total} = 1.68); **^{1}H NMR (400 MHz, CD_3OD)** δ 7.36–7.32 (2H, m), 7.28 (1H, d, *J*=8.1 Hz), 7.11 (2H, s), 6.79 (0.32H, d, *J*=8.7 Hz, labeled 84%D), 4.99–4.95 (1H, bs), 4.12–4.06 (1H, m), 4.06–3.96 (1H, m), 3.95–3.79 (5H, m), 3.69 (1H, dd, *J*=12.0, 5.4 Hz), 3.50–3.38 (3H, m), 3.31–3.27 (2H, m) 2.24–2.15 (1H, m), 2.11–2.03 (1H, m); **^{13}C NMR (101 MHz, CD_3OD)** δ 157.2, 140.1, 139.8, 134.4, 133.4, 131.9, 131.0, 130.1, 128.3, 116.5–116.0 (m, labeled), 82.9, 82.2, 79.7, 78.5, 76.5, 74.0, 71.8, 68.1, 63.1, 39.2, 33.9; **HRMS** (ESI) Calculated for $C_{23}H_{27}ClO_7Na$ $[M+Na]^+$ 473.1343 found 473.1330 (3%); Calculated for $C_{23}H_{26}DClO_7Na$ $[M+Na]^+$ 474.1406 found 474.1391 (29%); Calculated for $C_{23}H_{25}D_2ClO_7Na$ $[M+Na]^+$ 475.1469 found 475.1451 (68%); D_{Total} = 1.65 calculated by HRMS.

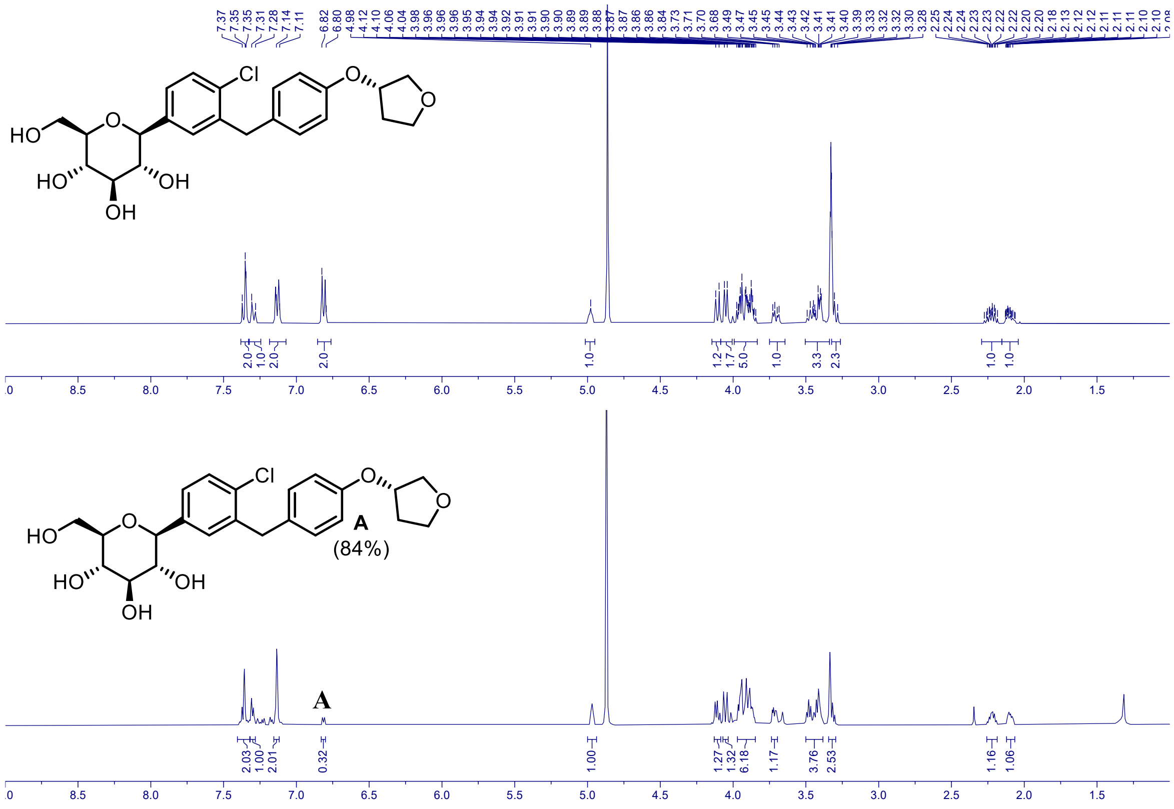

Figure S34. Stacked ^{1}H NMR (400 MHz, CD_3OD) spectra of **15**: natural abundance (top) and labelled (bottom).

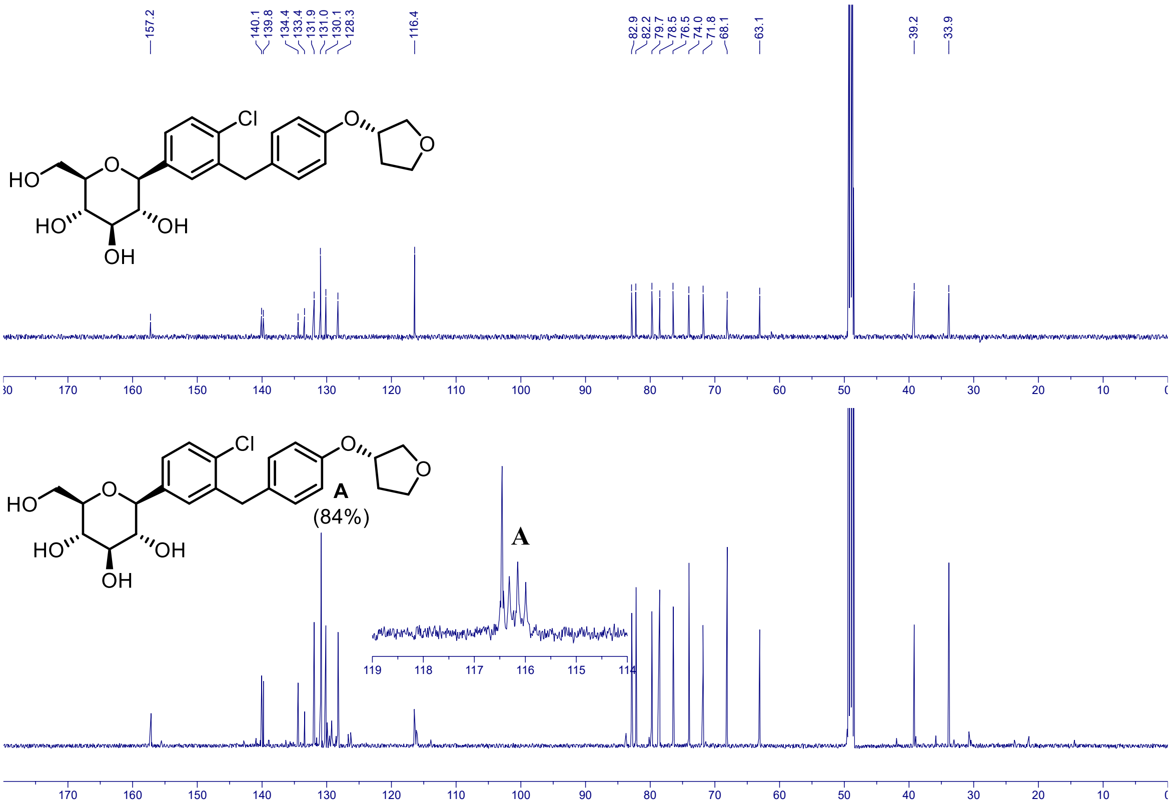

Figure S35. Stacked ^{13}C NMR (101 MHz, CD_3OD) spectra of **15**: natural abundance (top) and labelled (bottom).

[²H]Dopamine hydrochloride (16)

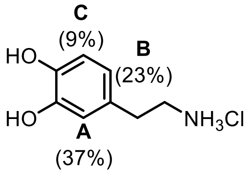

Obtained from dopamine hydrochloride following procedure **GP3**. [254 nm]. (55%, D_{Total} = 0.69); **^{1}H NMR (400 MHz, CD$_3$OD)** δ 6.74 (0.77H, d, *J*=8.0 Hz, labeled, 23% D), 6.69 (0.63H, d, *J*=2.1 Hz, labeled, 37% D), 6.58 (1H, d, *J*=8.1 Hz, labeled, 9% D), 3.10 (2H, t, *J*=7.6 Hz), 2.80 (2H, t, *J*=7.6 Hz); **^{13}C NMR (101 MHz, CD$_3$OD)** δ 146.7, 145.6, 129.2, 121.0, 116.8, 116.7–116.3 (m, labeled), 42.2, 34.0; **HRMS** (ESI) Calculated for $C_8H_{12}NO_2$ $[M+H]^+$ 154.0868 found 154.0862 (50%); Calculated for $C_8H_{11}DNO_2$ $[M+H]^+$ 155.0931 found 155.0924 (42%); Calculated for $C_8H_{10}D_2NO_2$ $[M+H]^+$ 156.0994 found 156.0986 (8%); D_{Total} = 0.58 calculated by HRMS.

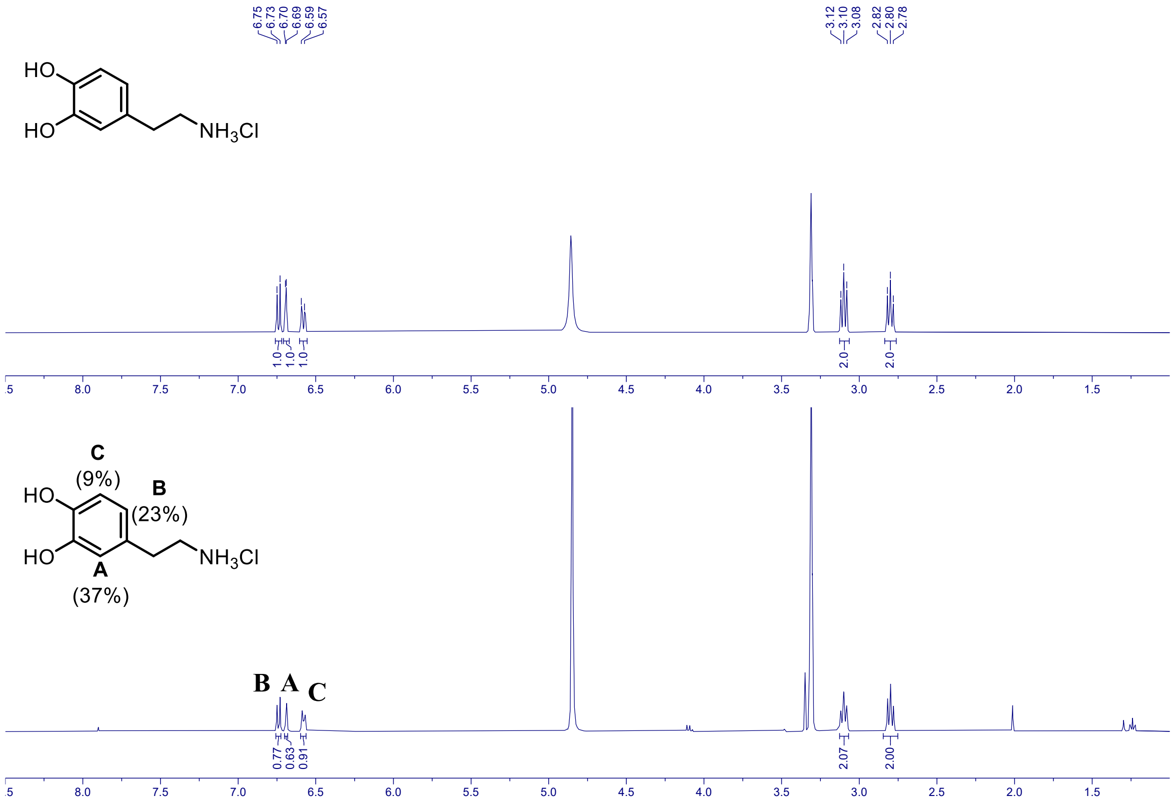

Figure S36. Stacked ^{1}H NMR (400 MHz, CD$_3$OD) spectra of **16**: natural abundance (top) and labelled (bottom).

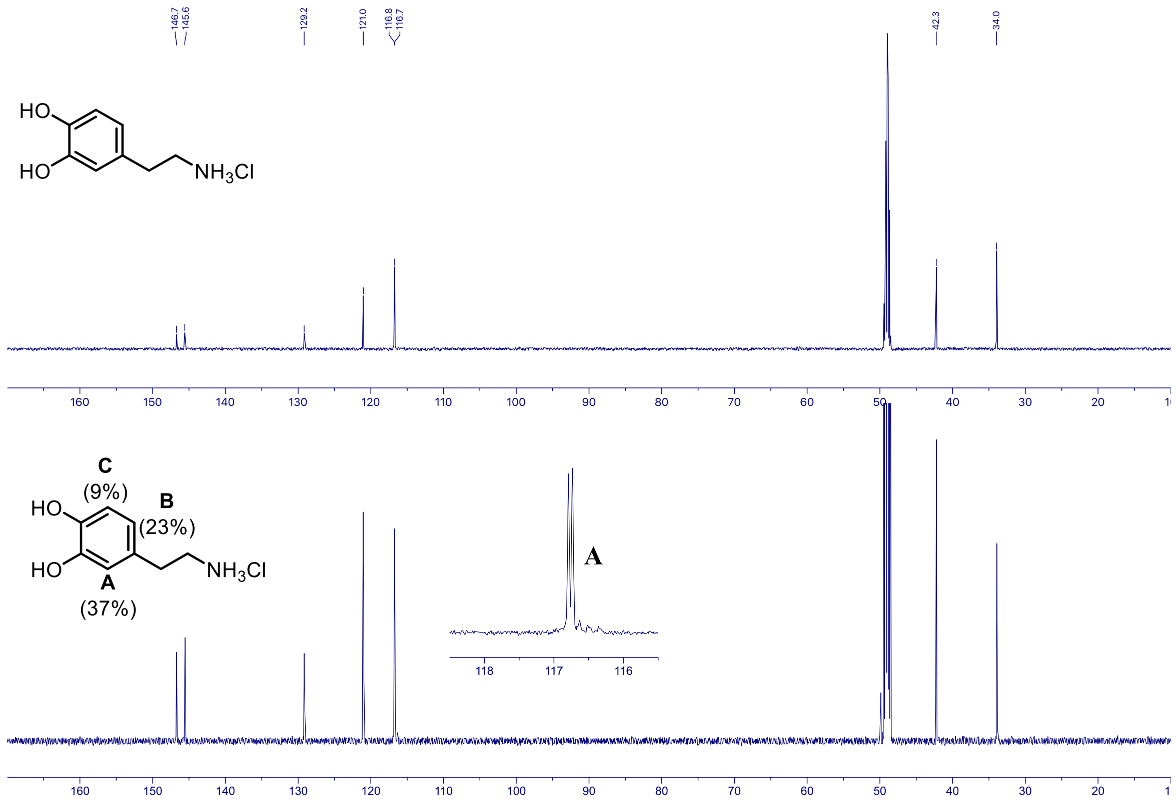

Figure S37. Stacked ^{13}C NMR (101 MHz, CD_3OD) spectra of **16**: natural abundance (top) and labelled (bottom).

[^{2}H]Dropropizine (17)

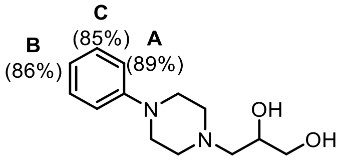

Obtained from dropropizine following procedure **GP1** [254 nm]. (>95%, D_{Total} = 4.34); **^{1}H NMR (600 MHz, CD_2Cl_2)** δ 7.32–7.25 (0.31H, m, labeled, 85% D), 6.98–6.94 (0.22H, m, labeled, 89% D), 6.90–6.85 (0.14H, m, labeled, 86% D), 3.89 (1H, dq, *J*=8.7, 4.1 Hz), 3.73 (1H, dd, *J*=11.4, 3.8 Hz), 3.53 (1H, dd, *J*=11.4, 4.8 Hz), 3.28–3.17 (5H, m), 2.86 (2H, ddd, *J*=10.7, 6.4, 3.6 Hz), 2.72–2.60 (3H, m), 2.46 (1H, dd, *J*=12.5, 4.0 Hz); **^{13}C NMR (151 MHz, $CDCl_3$) δ** 151.8, 129.4–128.7 (m, labeled), 119.9–119.3 (m, labeled), 116.3–115.7 (m, labeled), 67.4, 65.3, 60.8, 53.6, 49.6; **HRMS** (ESI) Calculated for $C_{13}H_{19}D_2N_2O_2$ $[M+H]^+$ 239.17286 found 239.17207 (2%); Calculated for $C_{13}H_{18}D_3N_2O_2$ $[M+H]^+$ 240.1791 found 240.1782 (14%), Calculated for $C_{13}H_{17}D_4N_2O_2$ $[M+H]^+$ 241.1854 found 241.1843 (37%); Calculated for $C_{13}H_{16}D_5N_2O_2$ $[M+H]^+$ 242.1917 found 242.1902 (47%); D_{Total} = 4.29 calculated by HRMS.

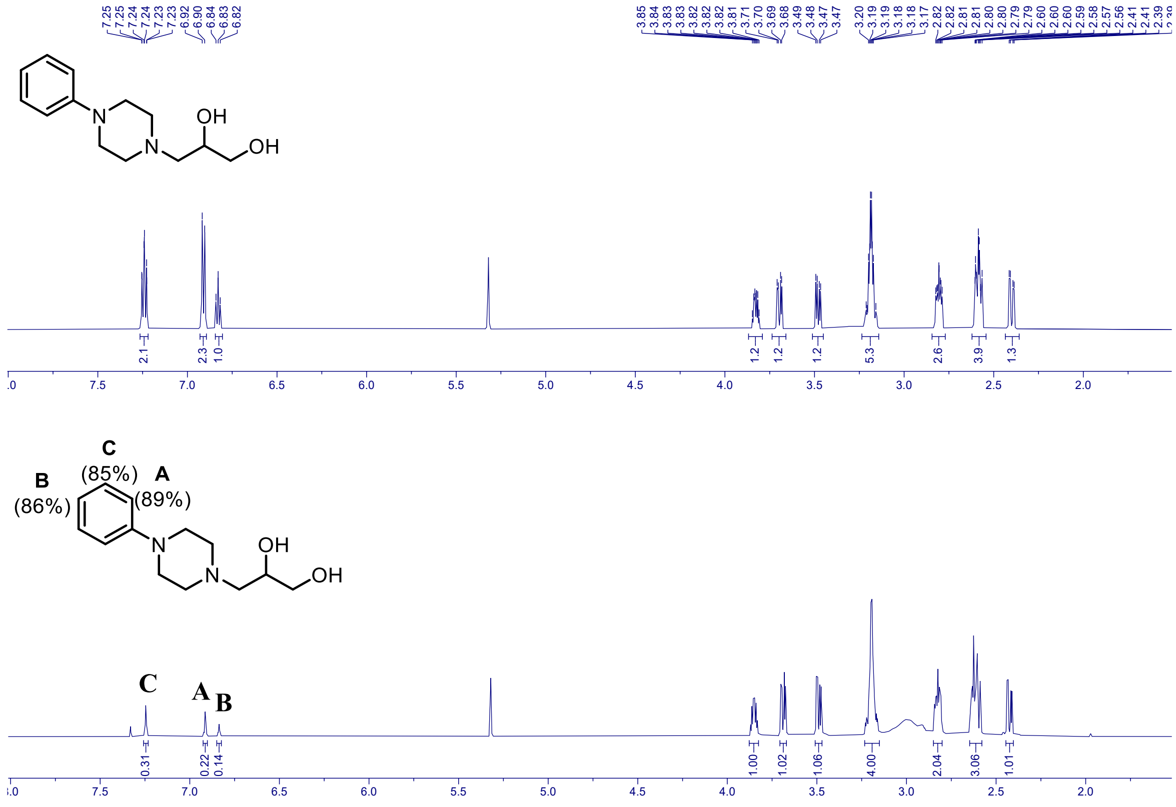

Figure S38. Stacked ^{1}H NMR (600 MHz, CD_2Cl_2) spectra of **17**: natural abundance (top) and labelled (bottom).

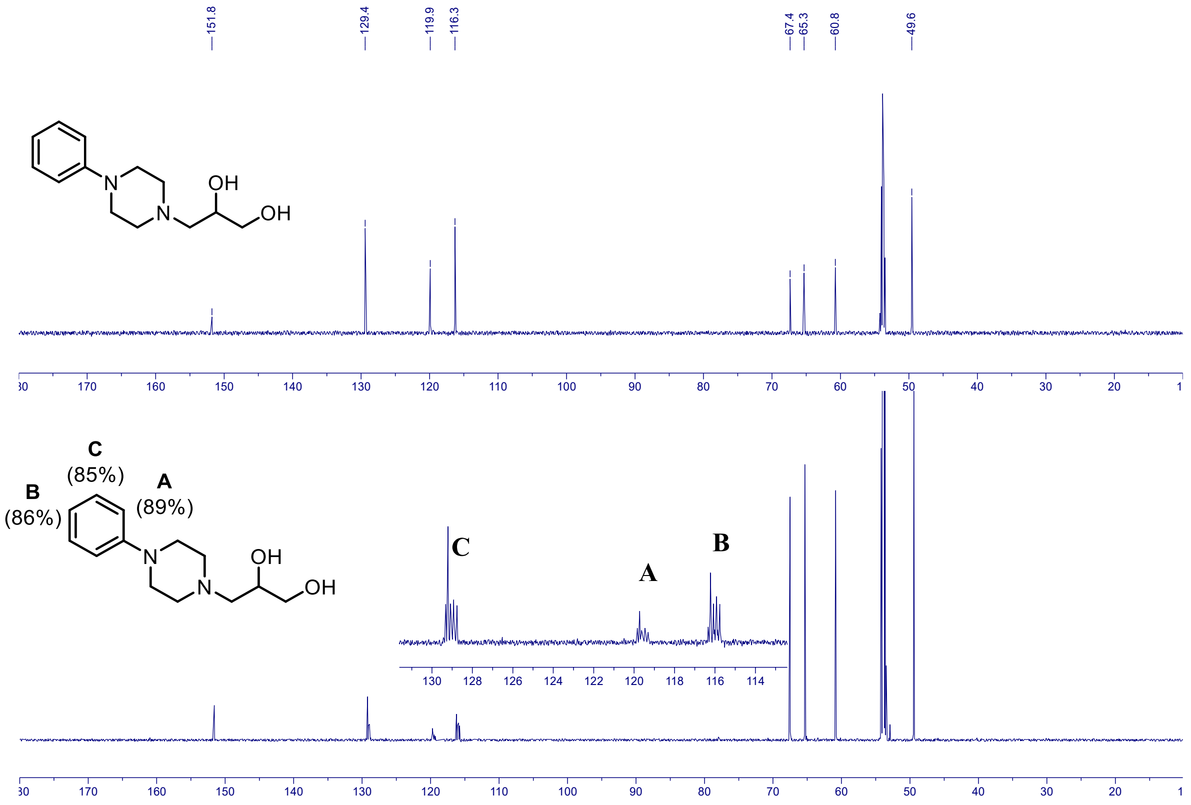

Figure S39. Stacked ^{13}C NMR (151 MHz, CD_2Cl_2) spectra of **17**: natural abundance (top) and labelled (bottom).

[^{2}H]Paroxetine (18)

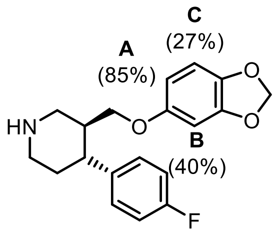

Obtained from paroxetine hydrochloride following procedure **GP1** [310 nm]. Extraction $NaHCO_3$/EtOAc and purification by prep TLC. (70%, D_{Total} = 1.52); **^{1}H NMR (400 MHz, CD_2Cl_2)** δ 7.20 (2H, dd, J=8.5, 5.6 Hz), 6.98 (2H, t, J=8.8 Hz), 6.67–6.54 (0.73H, m, labeled, 27% D), 6.33 (0.6H, s, labeled, 40% D), 6.13 (0.15H, dd, J=8.5, 2.5 Hz, labeled, 85% D), 5.86 (2H, s), 3.56 (1H, dd, J=9.4, 3.0 Hz), 3.43 (1H, dd, J=9.4, 7.4 Hz), 3.38 (1H, dd, J=12.4, 3.9 Hz), 3.13 (1H, d, J=12.2 Hz), 2.71 (1H, td, J=12.2, 2.8 Hz), 2.62 (1H, d, J=12.2 Hz), 2.57 (1H, td, J=11.7, 4.0 Hz), 2.07–2.00 (1H, m), 1.91 (1H, bs), 1.77 (1H, dq, J=13.0, 2.9 Hz), 1.68 (1H, qd, J=12.4, 4.1 Hz); **^{13}C NMR (101 MHz, CD_2Cl_2)** δ 161.9 (d, $J_{C\text{-}F}$ = 243.4 Hz), 154.8, 148.6, 142.0, 140.3, 129.3 (d, $J_{C\text{-}F}$ = 7.9 Hz), 115.6 (d, $J_{C\text{-}F}$ = 21.2 Hz), 108.3–108.0 (m, labeled), 106.0–105.0 (m, labeled), 101.7, 98.2–97.8 (m, labeled), 69.6, 50.5, 49.8, 46.7, 44.4, 42.5, 34.7; **HRMS** (EI) Calculated for $C_{19}H_{20}NO_3F$ $[M]^+$ 329.1422 found 329.1422 (14%); Calculated for $C_{19}H_{19}DNO_3F$ $[M]^+$ 330.1482 found 330.1485 (64%); Calculated for $C_{19}H_{18}D_2NO_3F$ $[M]^+$ 331.1547 found 331.1547 (22%); D_{Total} = 1.08 calculated by HRMS.

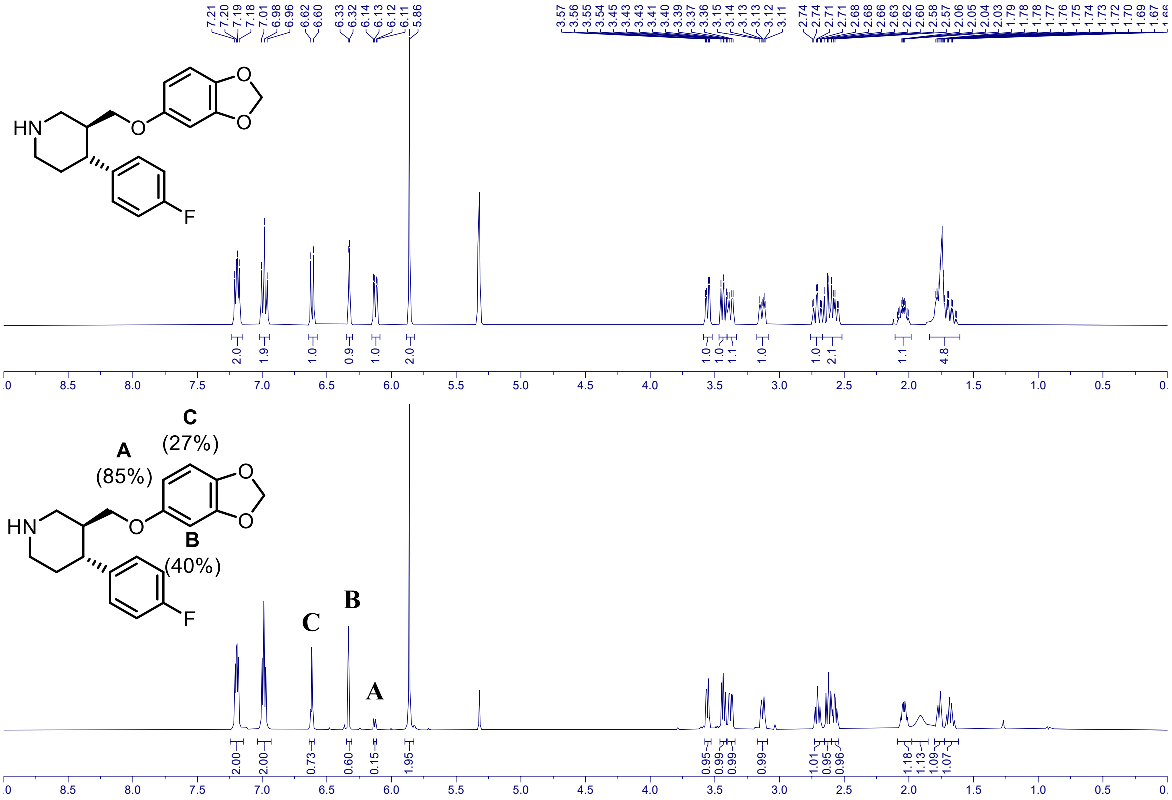

Figure S40. Stacked ^{1}H NMR (400 MHz, CD_2Cl_2) spectra of **18**: natural abundance (top) and labelled (bottom).

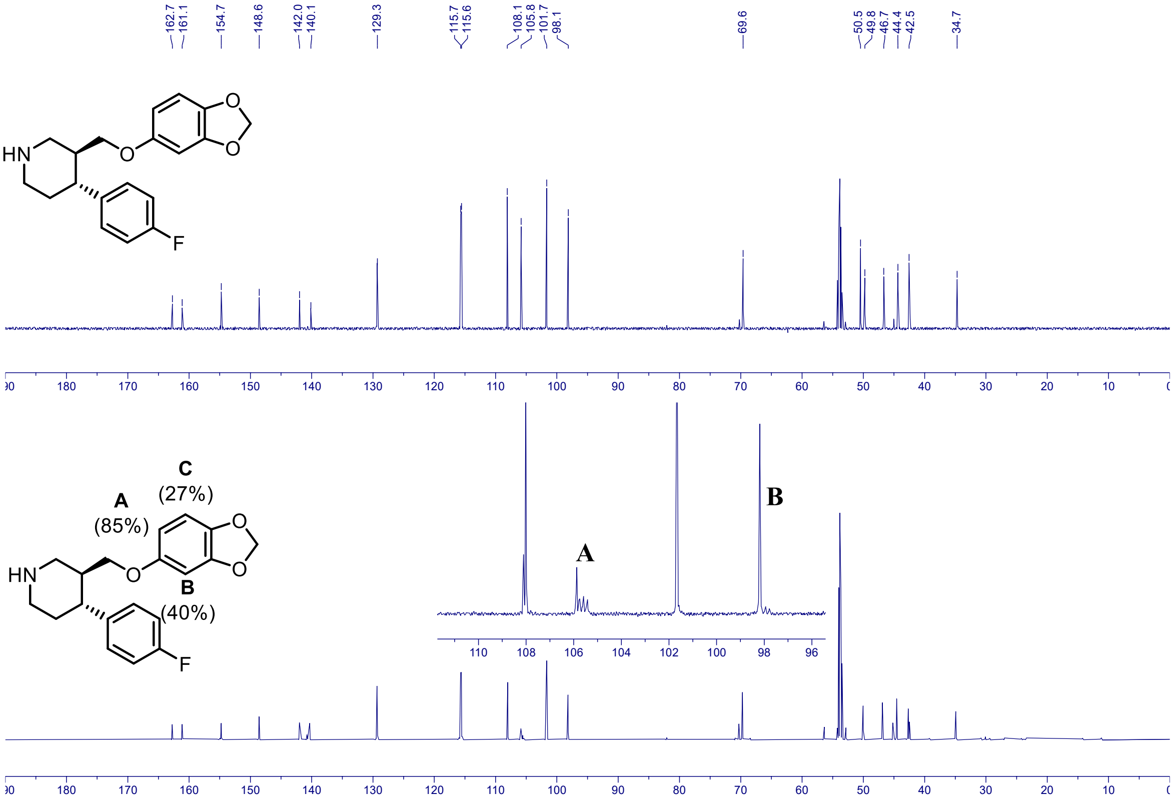

Figure S41. Stacked ^{13}C NMR (101 MHz, CD_2Cl_2) spectra of **18**: natural abundance (top) and labelled (bottom).

[^{2}H]*N*-Napthylparoxetine (19)

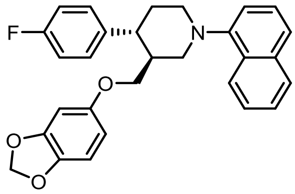

Obtained from *N*-napthylparoxetine following procedure **GP2** [370 nm]. (72%, $D_{Total} = 0.90$); **^{1}H NMR (600 MHz, CDCl$_3$)**: 8.25 (1H, d, *J*=8.2 Hz), 7.86–7.83 (0.1H, m, labeled 90% D), 7.57 (1H, d, *J*=8.2 Hz), 7.54–7.46 (2H, m), 7.42 (1H, t, *J*=7.8 Hz), 7.30 (2H, t, *J*=6.8 Hz), 7.15 (1H, d, *J*=7.4 Hz), 7.07–6.99 (2H, m), 6.62 (1H, dd, *J*=8.4, 1.5 Hz), 6.33 (1H, t, *J*=2.0 Hz), 6.13 (1H, dt, *J*=8.7, 2.0 Hz), 5.87 (2H, d, *J*=1.6 Hz), 3.83–3.76 (1H, m), 3.67 (1H, dd, *J*=8.9, 2.5 Hz), 3.59–3.51 (2H, m), 2.94–2.82 (2H, m), 2.69 (1H, td, *J*=11.8, 4.0 Hz), 2.59–2.50 (1H, m), 2.25–2.16 (1H, m), 2.03–1.98 (1H, m); **^{13}C NMR (151 MHz, CD$_3$OD)**: (deuterated carbon is not seen due to long T_1 relaxation time) δ 161.8 (d, $J_{C\text{-}F}$ = 244.6 Hz), 154.4, 150.3, 148.3, 141.7, 139.9 (d, $J_{C\text{-}F}$ = 3.3 Hz), 134.9, 129.2, 129.1 (d, $J_{C\text{-}F}$ = 7.7 Hz), 126.0, 125.8, 125.5, 123.8, 123.5, 115.7 (d, $J_{C\text{-}F}$ = 21.1 Hz), 114.9, 108.0, 105.8, 101.2, 98.2, 69.6, 57.5, 54.5, 44.5, 43.0, 35.3; **HRMS** (ESI) calculated for $C_{29}H_{26}DFNO_3$ $[M+H]^+$ 457.2038 found 457.2032 (98%); calculated for $C_{29}H_{25}D_2FNO_3$ $[M+H]^+$ 458.2101 found 458.2108 (2%); $D_{Total} = 1.02$ calculated by HRMS.

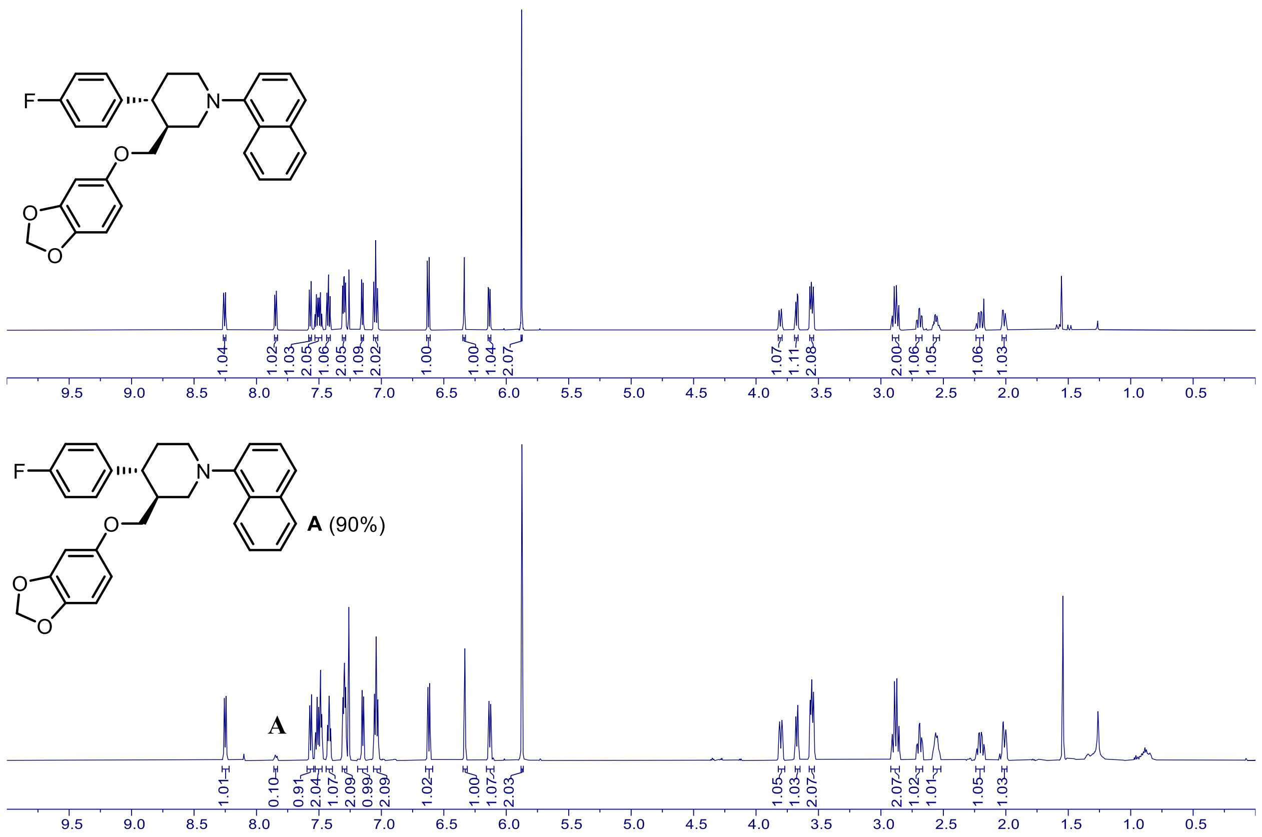

Figure S42. Stacked ^{1}H NMR (600 MHz, $CDCl_3$) spectra of **19**: natural abundance (top) and labelled (bottom).

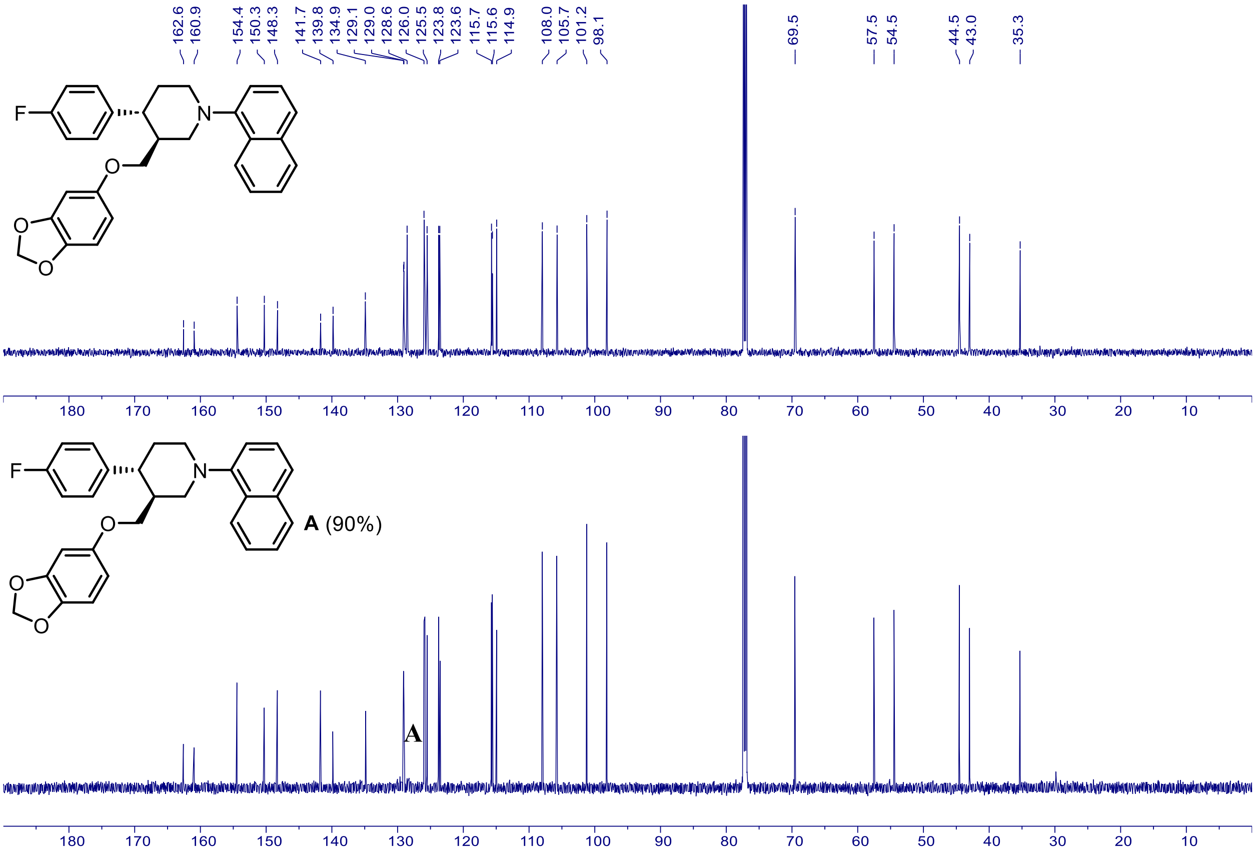

Figure S43. Stacked ^{13}C NMR (151 MHz, $CDCl_3$) spectra of **19**: natural abundance (top) and labelled (bottom).

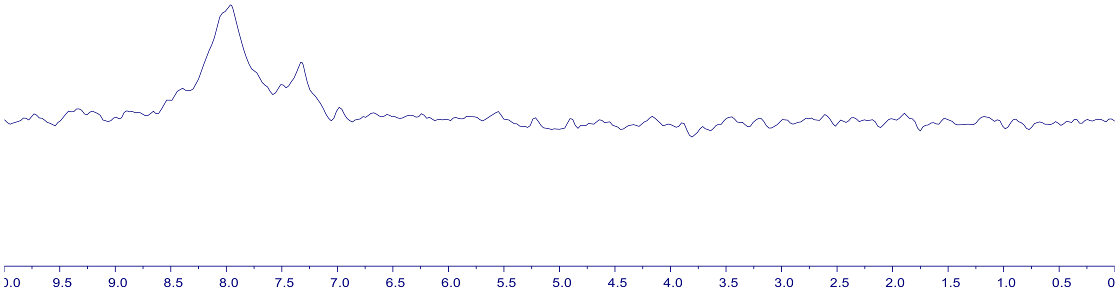

Figure S44. ^2H NMR (61 MHz, $CHCl_3$) spectra of **19**.

[^{2}H]Dehydroabetic acid (20)

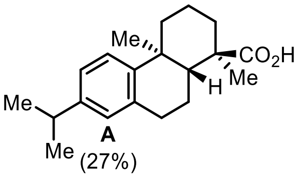

Obtained from dehydroabetic acid following procedure **GP1**. [254 nm]. (46%, D_{Total} = 0.27); **^{1}H NMR (400 MHz, CDCl$_3$)** δ 7.17 (1H, d, *J*=8.1 Hz), 7.00 (1H, d, *J*=8.0 Hz), 6.88 (0.73H, s, labeled, 27% D), 3.04–2.78 (3H, m), 2.31 (1H, d, *J*=12.9 Hz), 2.24 (1H, d, *J*=12.4 Hz), 1.82–1.69 (4H, m), 1.57–1.47 (3H, m), 1.28 (3H, s), 1.23 (3H, s), 1.23–1.18 (6H, m),; **^{13}C NMR (101 MHz, CDCl$_3$)** δ 184.9, 146.9, 145.9, 134.9, 127.1, 124.3, 124.0, 47.6, 44.7, 38.0, 37.0, 36.9, 33.6, 30.2, 25.3, 24.1, 21.9, 18.7, 16.4; **HRMS** not found.

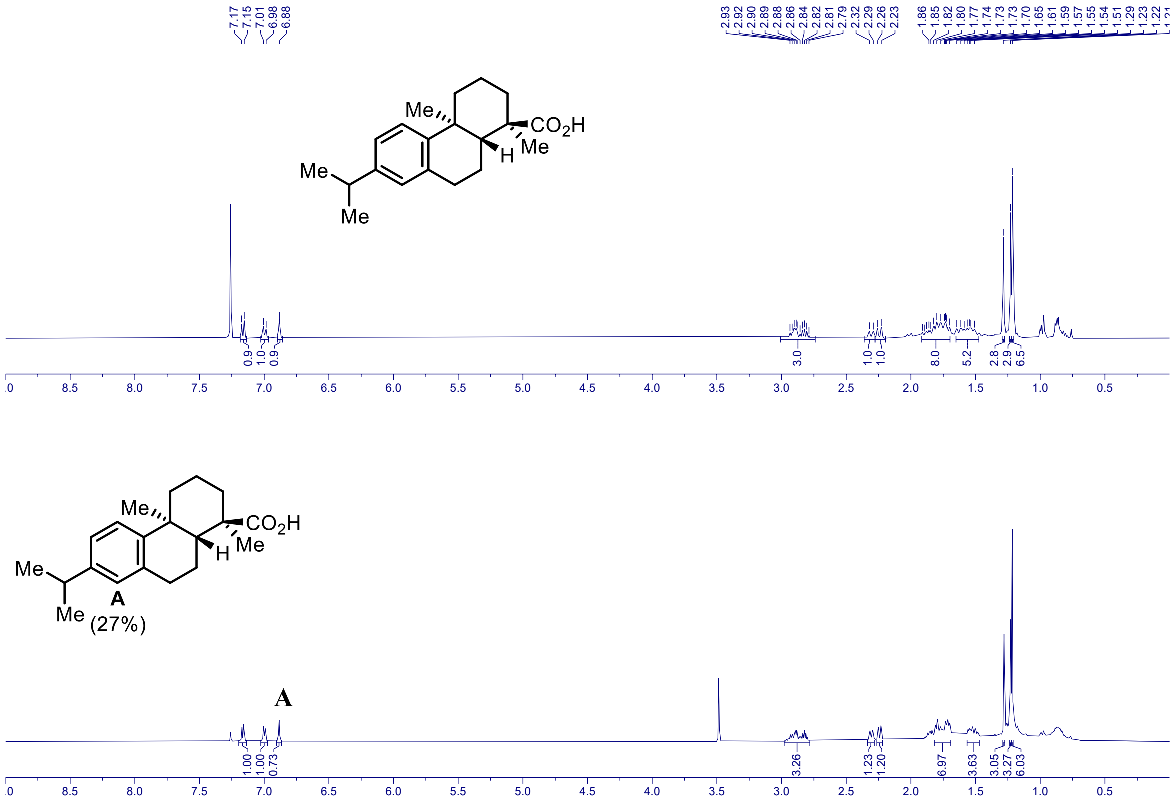

Figure S45. Stacked ^{1}H NMR (400 MHz, CDCl$_3$) spectra of **20**: natural abundance (top) and labelled (bottom).

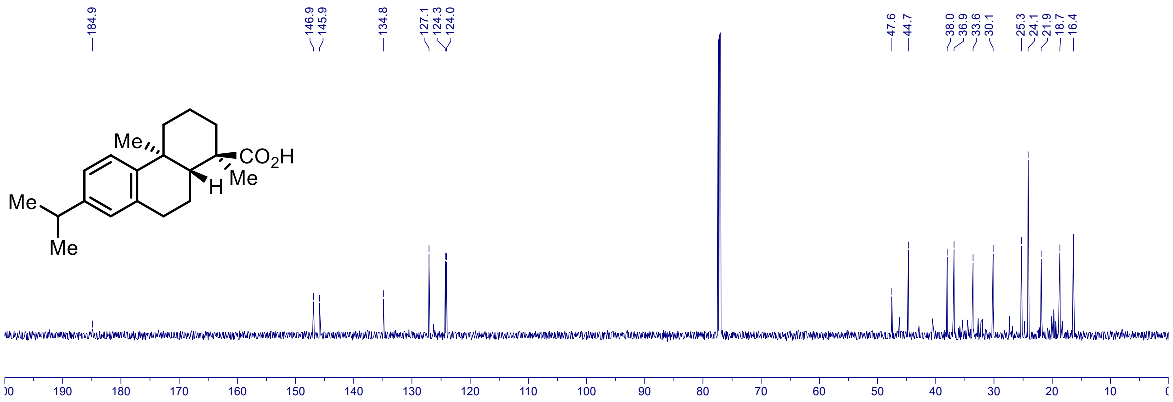

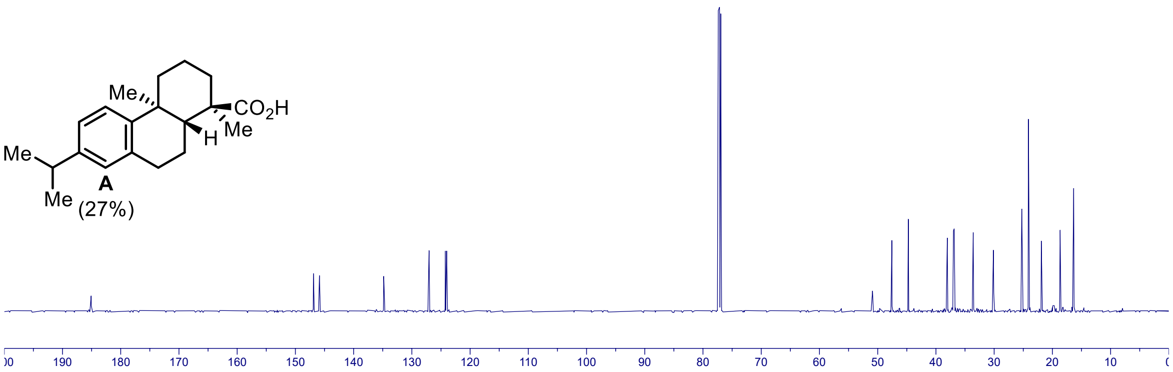

Figure S46. Stacked ^{13}C NMR (101 MHz, $CDCl_3$) spectra of **20**: natural abundance (top) and labelled (bottom).

[^{2}H]Imipramine hydrochloride (21)

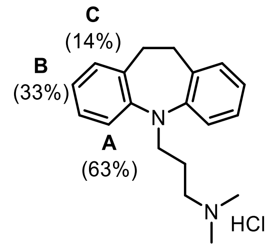

Obtained from imipramine hydrochloride following procedure **GP1**. [254 nm]. (>95%, D_{Total} = 2.20); **^{1}H NMR (600 MHz, acetone-*d*$_6$)** δ 7.20 (0.74H, d, *J*=8.0 Hz, labeled, 63% D), 7.15–7.08 (3.73H, m, labeled, 14% D), 6.92 (1.34H, t, *J* = 7.3 Hz, labeled, 33% D), 3.92 (2H, t, *J*=6.8 Hz), 3.22–3.07 (6H, m), 2.67 (6H, d, *J*=1.4 Hz), 2.16–2.03 (3H, m); **^{13}C NMR (151 MHz, acetone-*d*$_6$)** δ 149.0, 135.0, 130.7, 127.4, 123.6–123.2 (m, labeled), 120.8–120.3 (m, labeled), 56.1, 48.7, 42.4, 32.7, 23.4; **HRMS** (EI) Calculated for $C_{19}H_{24}N_2$ $[M]^+$ 280.1934 found 280.1936 (7%); Calculated for $C_{19}H_{23}DN_2$ $[M]^+$ 281.1997 found 281.1998 (27%); Calculated for $C_{19}H_{22}D_2N_2$ $[M]^+$ 282.2060 found 282.2060 (39%); Calculated for $C_{19}H_{21}D_3N_2$ $[M]^+$ 283.2122 found 283.2121 (22%); Calculated for $C_{19}H_{20}D_4N_2$ $[M]^+$ 284.2185 found 284.2183 (5%); D_{Total} = 1.91 calculated by HRMS.

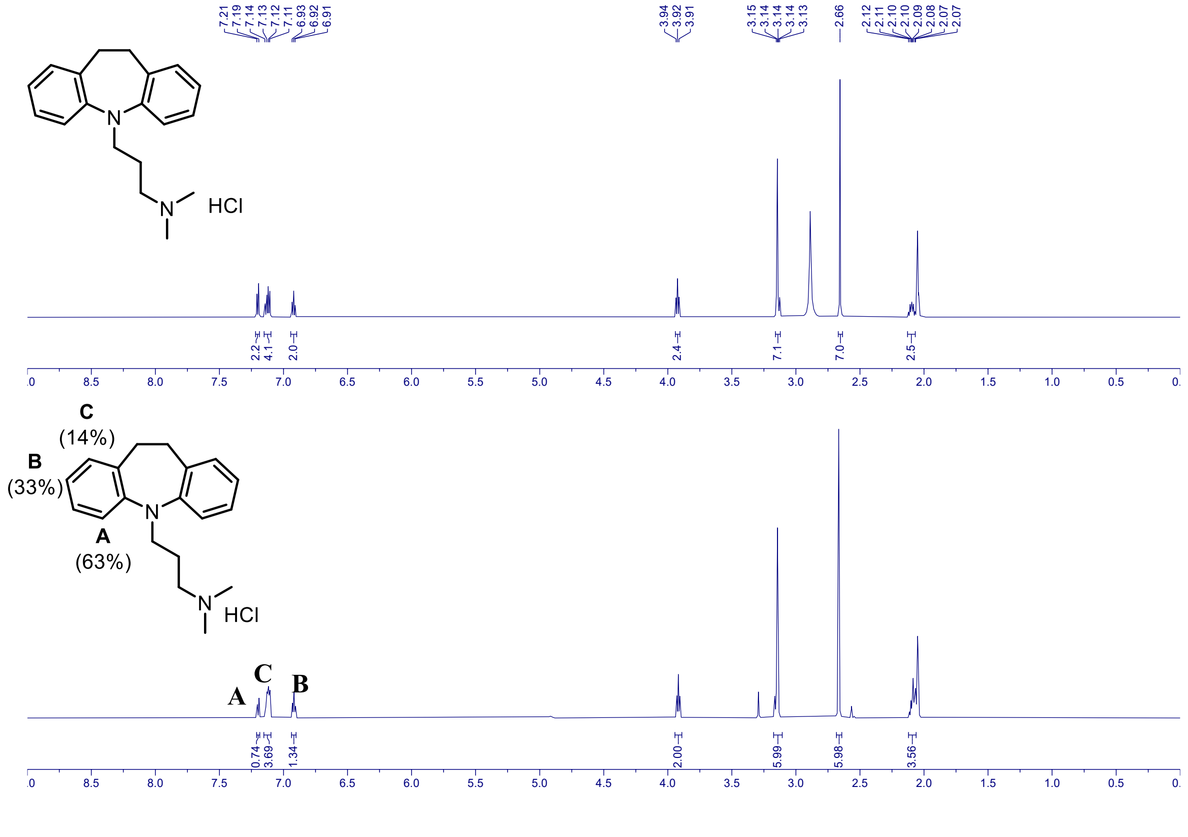

Figure S47. Stacked ^{1}H NMR (600 MHz, acetone-*d*$_6$) spectra of **21**: natural abundance (top) and labelled (bottom).

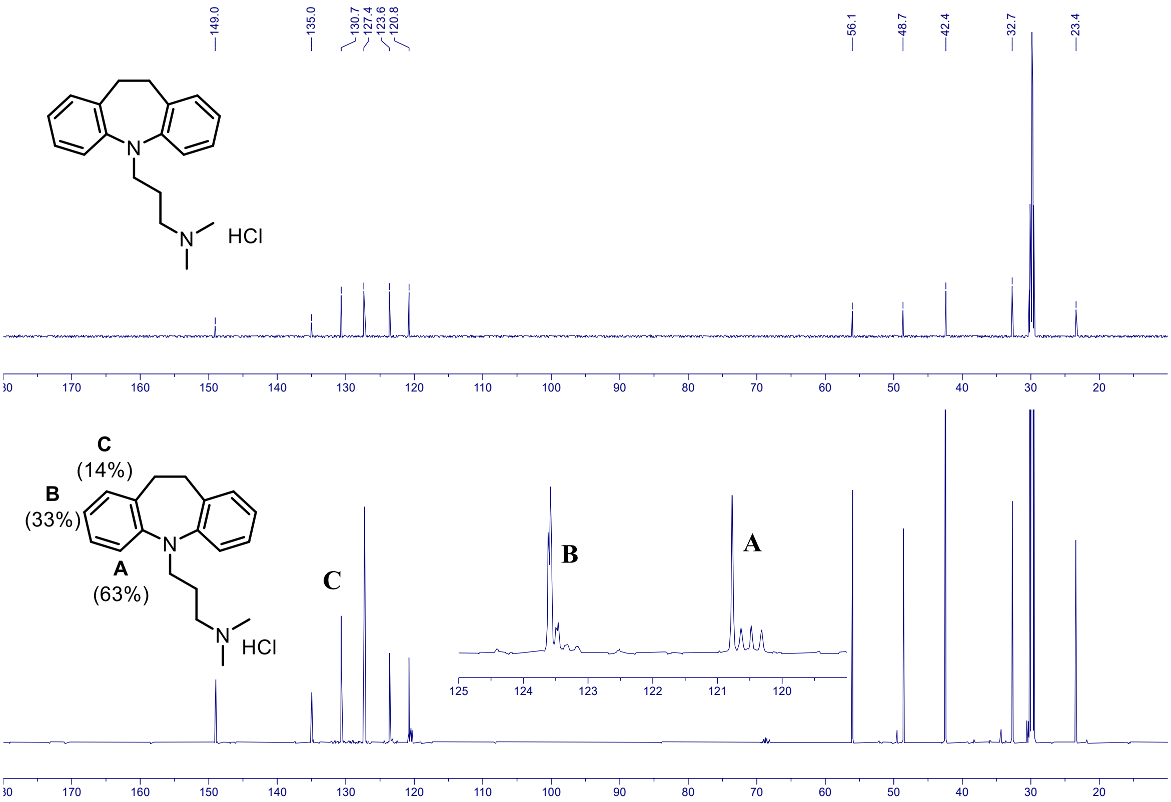

Figure S48. Stacked ^{13}C NMR (151 MHz, acetone-d_6) spectra of **21**: natural abundance (top) and labelled (bottom).

[^{2}H]Estradiol-17-valerate (22)

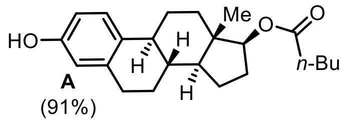

Obtained from estradiol-17-valerate following procedure **GP1** [254 nm]. (87%, D_{Total} = 0.91); **^{1}H NMR (600 MHz, $CDCl_3$)** δ 7.14 (1H, d, *J*=8.5 Hz), 6.63 (1H, d, *J*=8.5 Hz), 6.57 (0.09H, bs, labeled, >90% D), 5.03 (1H, bs), 4.70 (1H, t, *J*=8.5 Hz), 2.89–2.77 (2H, m), 2.33 (2H, t, *J*=7.5 Hz), 2.29–2.15 (3H, m), 1.90–1.82 (2H, m), 1.74 (1H, dddd, *J*=12.3, 9.8, 7.0, 3.1 Hz), 1.62 (2H, p, *J*=7.5 Hz), 1.54 (1H, dddd, *J*=13.8, 11.2, 7.7, 3.1 Hz), 1.48–1.32 (7H, m), 1.30–1.23 (1H, m), 0.93 (3H, t, *J*=7.4 Hz), 0.83 (3H, s); **^{13}C NMR (151 MHz, $CDCl_3$)**: δ 174.2, 153.4, 138.1, 132.5, 126.5, 115.0 (t, *J* = 23.1 Hz, labeled), 112.7, 82.6, 49.8, 43.8, 43.0, 38.6, 36.9, 34.4, 29.5, 27.6, 27.2, 27.2, 26.2, 23.3, 22.3, 13.7, 12.1; **HRMS** (ESI) calculated for $C_{23}H_{32}O_3Na$ $[M+Na]^+$ 379.2249 found 379.2245 (9%); calculated for $C_{23}H_{31}DO_3Na$ $[M+Na]^+$ 380.2312 found 380.2306 (82%); calculated for $C_{23}H_{30}D_2O_3Na$ $[M+Na]^+$ 381.2375 found 381.2348 (9%); D_{Total} = 1.0 calculated by HRMS.

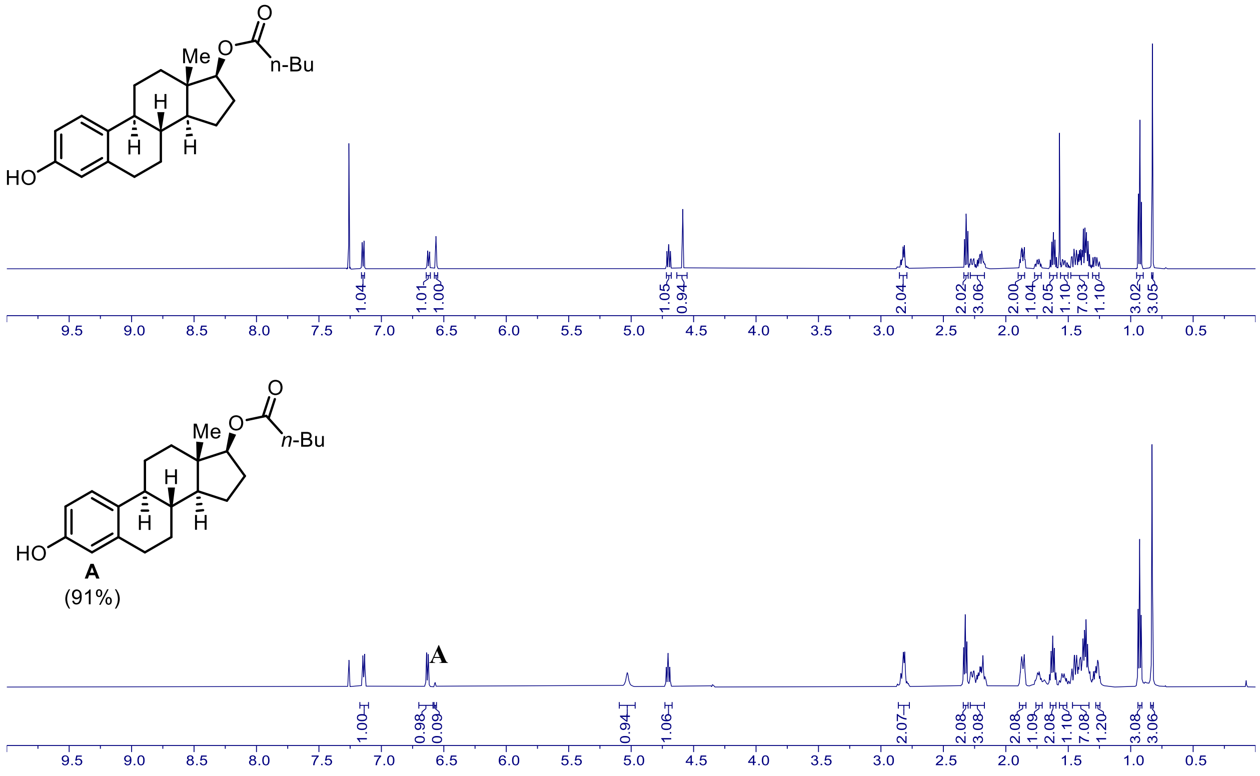

Figure S49. Stacked ^{1}H NMR (600 MHz, $CDCl_3$) spectra of **22**: natural abundance (top) and labelled (bottom).

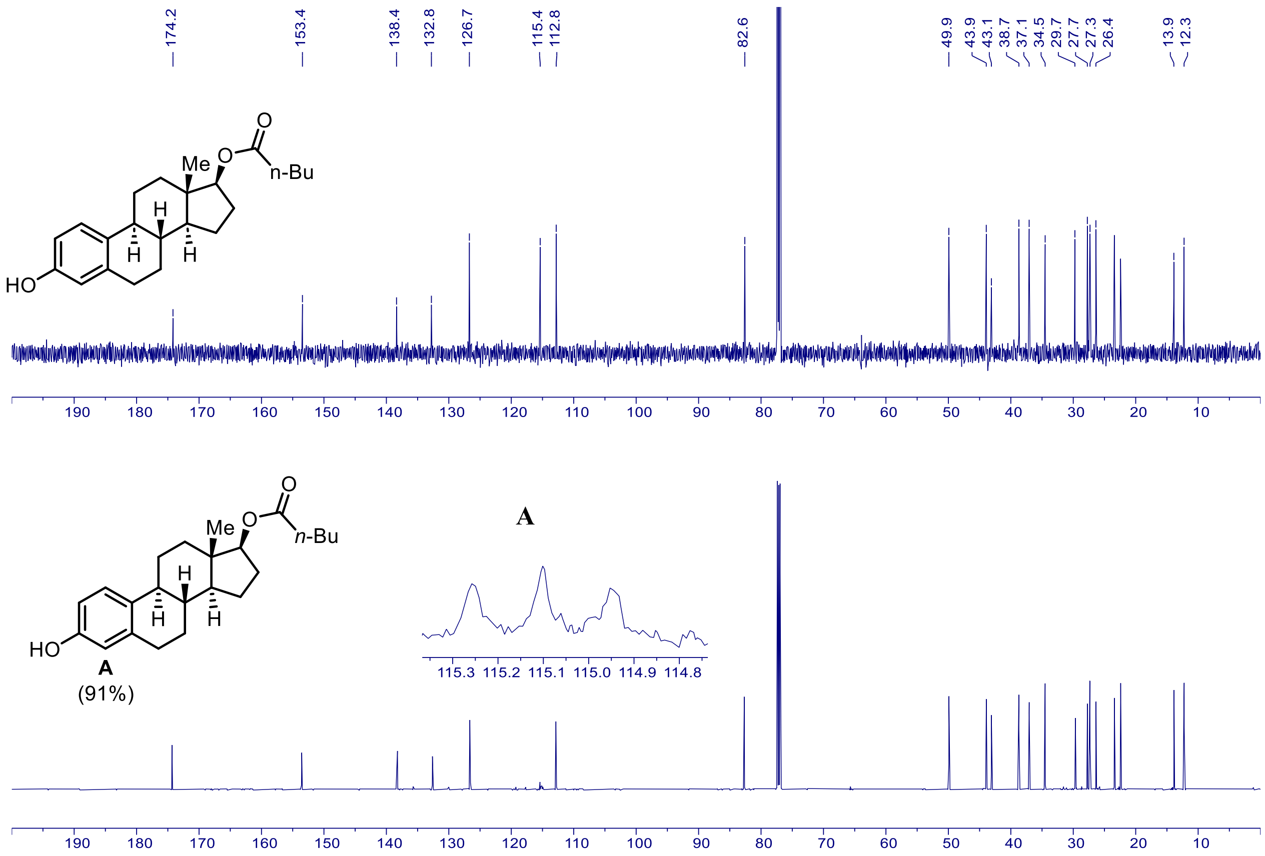

Figure S50. Stacked ^{13}C NMR (151 MHz, $CDCl_3$) spectra of **22**: natural abundance (top) and labelled (bottom)

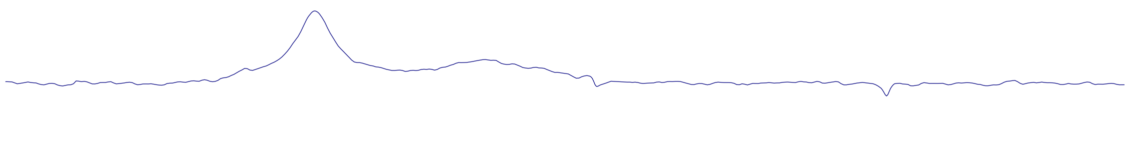

Figure S51. ^{2}H NMR (61 MHz, $CHCl_3$) spectra of **22**.

[^{2}H]Melatonin (23)

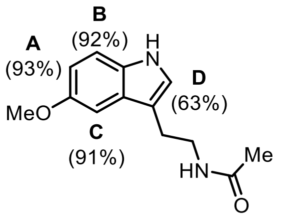

Obtained from melatonin following procedure **GP1** [254 nm]. (73%, D_{Total} = 3.39); **^{1}H NMR (400 MHz, CD_3CN)** δ 8.92 (1H, bs), 7.25 (0.08H, s, labeled, 92% D), 7.06 (0.09H, s, labeled, 91% D), 7.04 (0.37H, d, *J*=2.5 Hz, labeled, 63% D), 6.75 (0.07H, s, labeled, 93% D), 6.38 (1H, bs), 3.79 (3H, s), 3.39 (2H, t, *J*=7.2 Hz), 2.83 (2H, t, *J* = 7.2 Hz), 1.80 (3H, s); **^{13}C NMR (101 MHz, CD_2Cl_2)** δ 170.1, 154.4, 131.9, 128.2, 123.3–122.8 (m, labeled), 113.1, 112.7–112.4 (m, labeled), 112.3–111.7 (m, labeled), 100.7–100.2 (m, labeled), 56.1, 40.1, 25.7, 23.5; **HRMS** (ESI) Calculated for $C_{13}H_{15}DN_2O_2Na$ $[M+Na]^+$ 256.1172 found 256.1162 (1%); Calculated for $C_{13}H_{14}D_2N_2O_2Na$ $[M+Na]^+$ 257.1235 found 257.1226 (10%); Calculated for $C_{13}H_{13}D_3N_2O_2Na$ $[M+Na]^+$ 258.1298 found 258.1289 (46%); Calculated for $C_{13}H_{12}D_4N_2O_2Na$ $[M+Na]^+$ 259.1361 found 259.1351 (43%); D_{Total} = 3.31 calculated by HRMS.

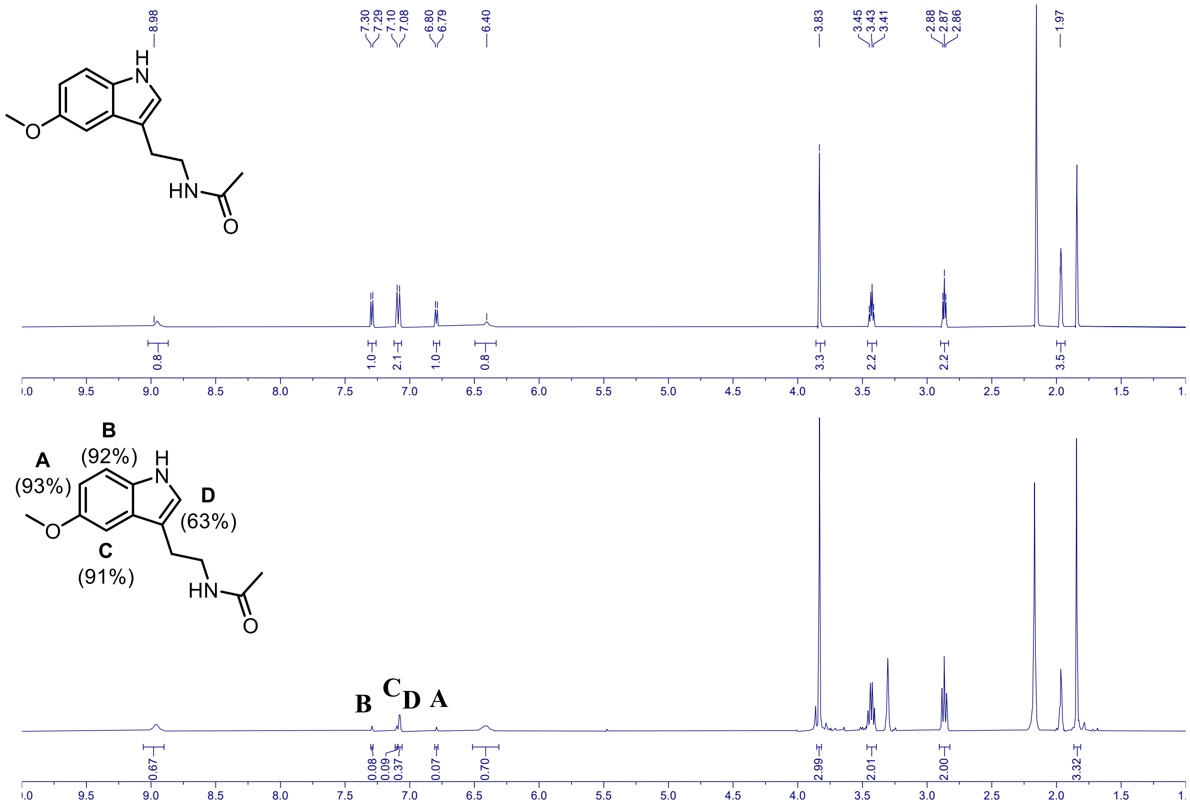

Figure S52. Stacked ^{1}H NMR (400 MHz, CD_3CN) spectra of **23**: natural abundance (top) and labelled (bottom).

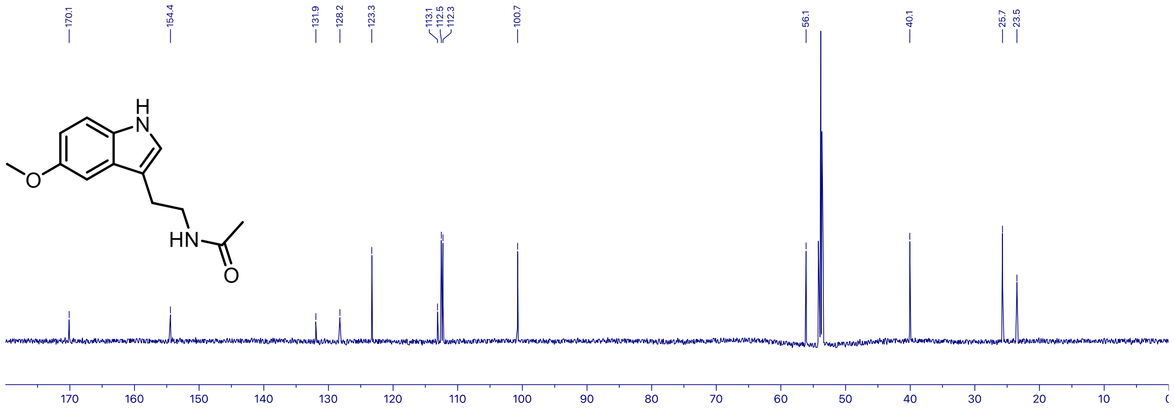

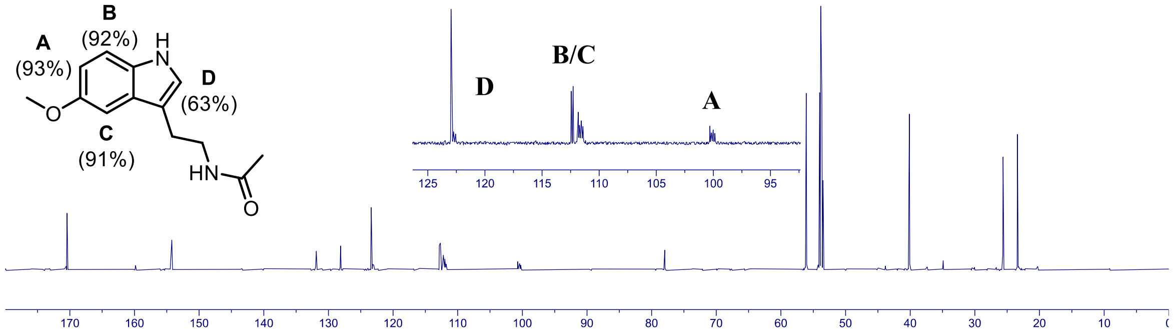

Figure S53. Stacked ^{13}C NMR (101 MHz, CD_3CN) spectra of **23**: natural abundance (top) and labelled (bottom).

[^{2}H]Serotonine hydrochloride (24)

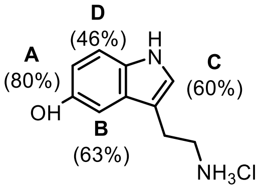

Obtained from serotonine hydrochloride following procedure GP1 [254 nm]. (50%, D_{Total} = 2.49); **^{1}H NMR (400 MHz, CD_3OD)** δ 7.20 (0.54H, s, <u>labeled 46% D</u>), 7.12–7.05 (0.40H, m, <u>labeled 60% D</u>), 6.92 (0.37H, s, <u>labeled 63% D</u>), 6.73–6.67 (0.20H, m, <u>labeled 80% D</u>), 3.21 (2H, t, *J*=7.4 Hz), 3.05 (2H, t, *J*=7.4 Hz); **^{13}C NMR (101 MHz, CD_3OD)** δ 151.5, 133.3, 128.9, 125.0–124.5 (<u>m, labeled</u>), 113.3–112.3 (2C <u>m, labeled</u>), 109.4, 103.1–102.8 (<u>m, labeled</u>), 41.1, 24.6; **HRMS** (ESI) Calculated for $C_{10}H_{12}DN_2O$ $[M+H]^+$ 178.10907 found 178.10850 (10%); Calculated for $C_{10}H_{11}D_2N_2O$ $[M+H]^+$ 179.1153 found 179.1148 (32%); Calculated for $C_{10}H_{10}D_3N_2O$ $[M+H]^+$ 180.1216 found 180.1209 (43%); Calculated for $C_{10}H_9D_4N_2O$ $[M+H]^+$ 181.1279 found 181.1271 (15%); D_{Total} = 2.63 calculated by HRMS.

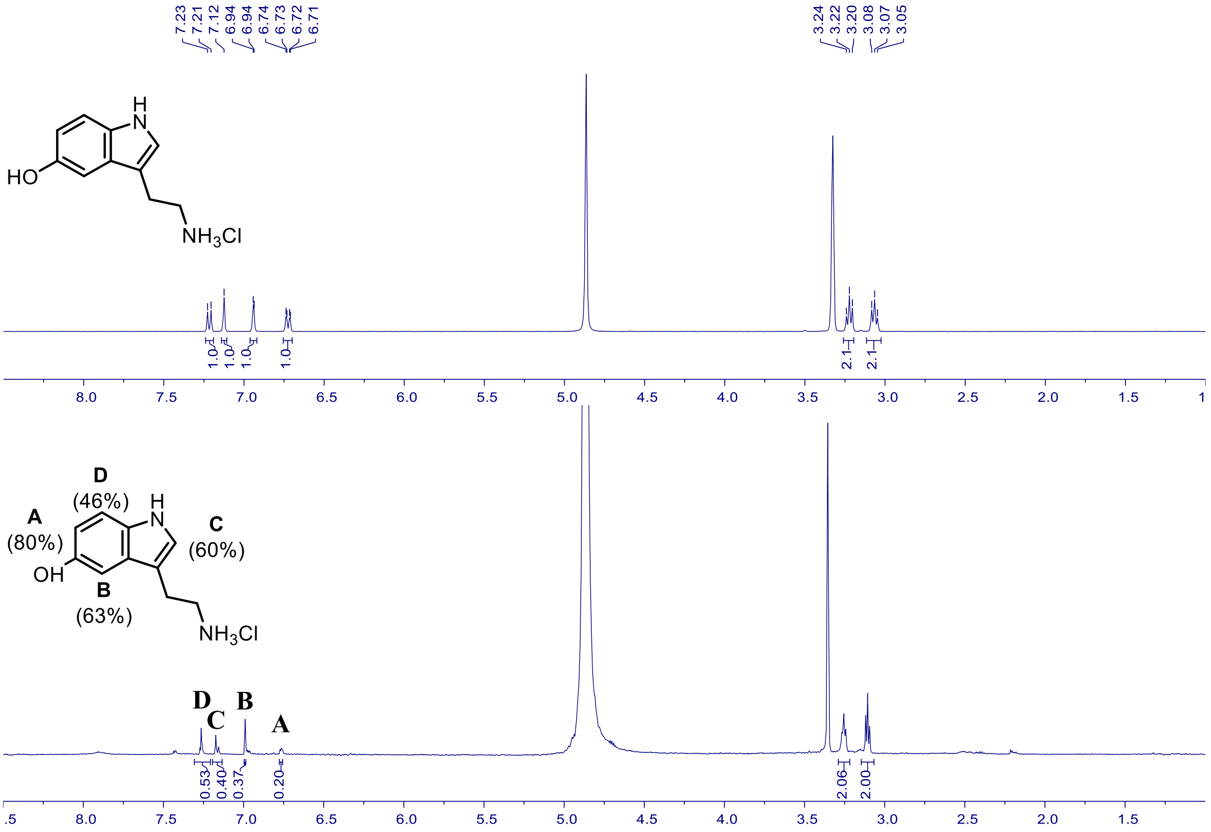

Figure S54. Stacked ^{1}H NMR (400 MHz, CD_3OD) spectra of **24**: natural abundance (top) and labelled (bottom).

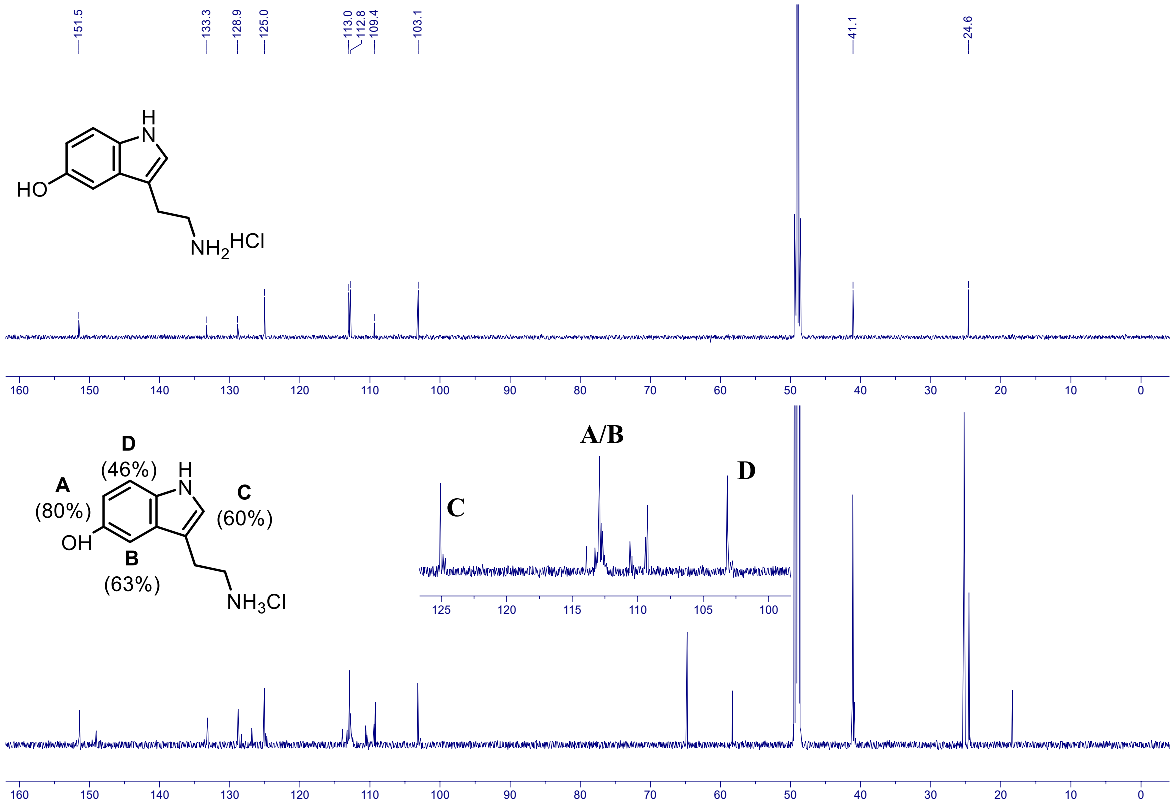

Figure S55. Stacked ^{13}C NMR (101 MHz, CD_3OD) spectra of **24**: natural abundance (top) and labelled (bottom).

[^{2}H]Tryptophan (25)

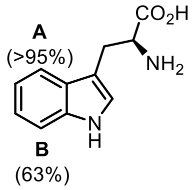

Obtained from *L*-tryptophan following procedure **GP1** [254 nm]. (61%, D_{Total} = 1.63); **^{1}H NMR (600 MHz, CD_3OD)**: δ 7.70 (0.04H, d, *J*=7.9 Hz, labeled >95% D), 7.37 (0.37H, d, *J*=8.1 Hz, labeled 63% D), 7.20 (1H, s), 7.14–7.10 (1H, m), 7.05 (1H, d, *J*=7.1 Hz), 3.87 (1H, dd, *J*=9.4, 4.1 Hz), 3.52 (1H, dd, *J*=15.2, 4.1 Hz), 3.16 (1H, dd, *J*=15.2, 9.4 Hz); **^{13}C NMR (151 MHz, CD_3OD)**: (one deuterated carbon is not seen due to long T_1 relaxation time) δ 173.0, 137.0, 127.0, 123.7, 121.2, 118.6, 111.2–111.0 (m, labeled), 108.2, 55.3, 27.1; **HRMS** (ESI) calculated for $C_{11}H_{11}DN_2O_2$ $[M]^+$ 205.0962 found 205.1636 (20%); calculated for $C_{11}H_{10}D_2N_2O_2$ $[M]^+$ 206.1024 found 206.1033 (17%); calculated for $C_{11}H_9D_3N_2O_2$ $[M]^+$ 207.1087 found 207.1096 (50%); calculated for $C_{11}H_8D_4N_2O_2$ $[M]^+$ 208.1150 found 208.1158 (13%); D_{Total} = 2.56 calculated by HRMS.

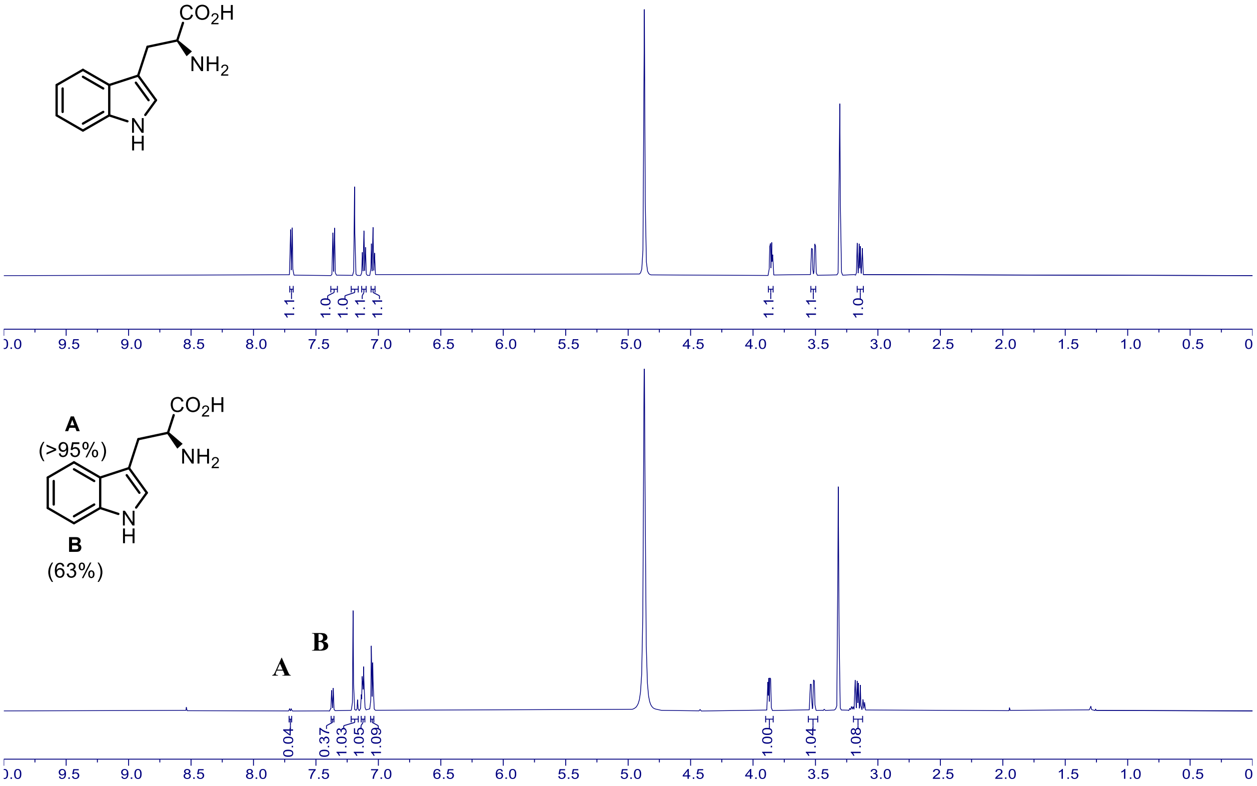

Figure S56. Stacked ^{1}H NMR (600 MHz, CD_3OD) spectra of **25**: natural abundance (top) and labelled (bottom).

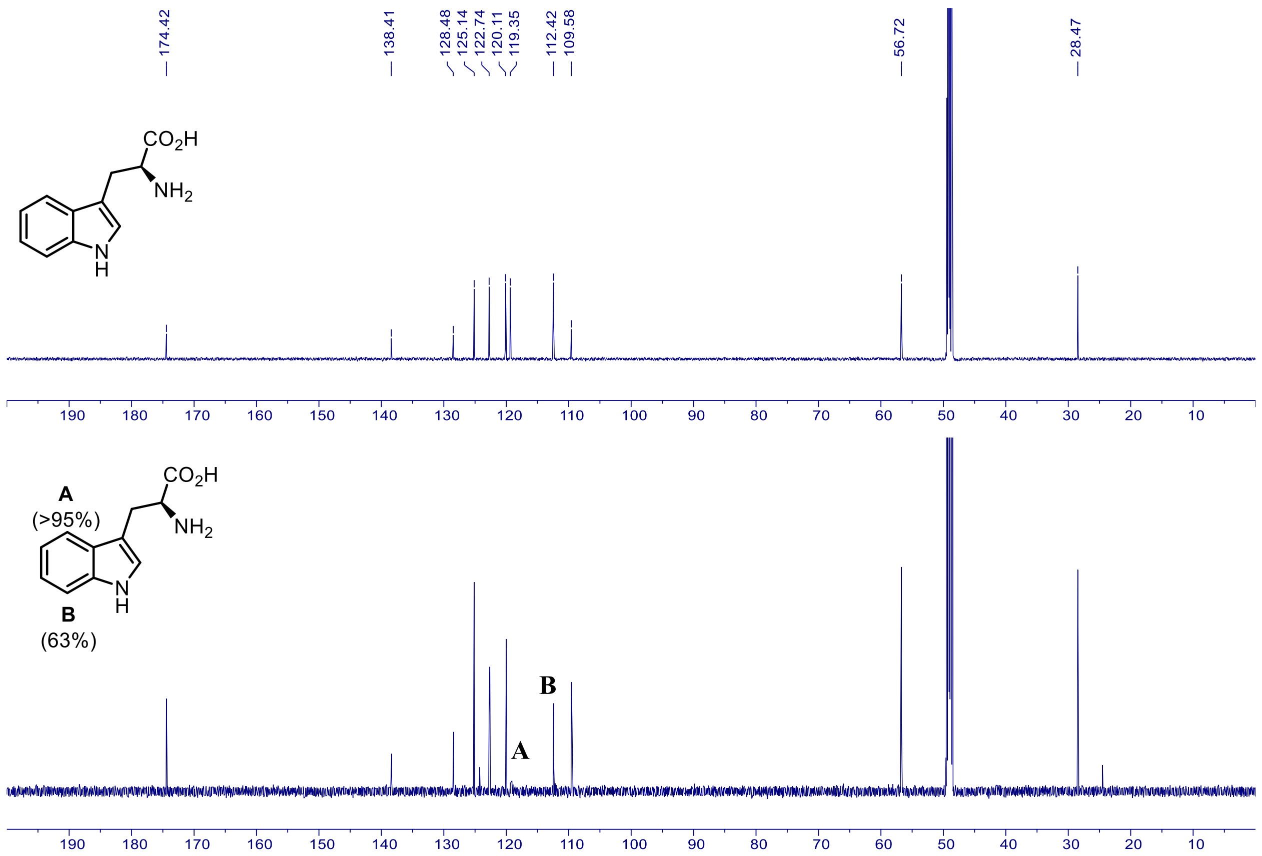

Figure S57. Stacked ^{13}C NMR (151 MHz, CD_3OD) spectra of **25**: natural abundance (top) and labelled (bottom).

[²H]*L*-Trp, *N*-Cbz-*L*-Met (26)

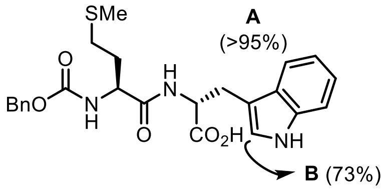

Obtained from *L*-trp, *N*-cbz-*L*-met following procedure **GP1** [254 nm]. (53%, D_{Total} = 1.73); **^{1}H NMR (600 MHz, CD_3OD)**: δ 7.39–7.21 (6H, m), 7.12 (0.27H, bs, labeled 73%D), 7.08 (1H, d, *J*=6.8 Hz), 7.00 (7H, d, *J*=6.8 Hz), 5.08 (1H, d, *J*=12.4 Hz), 5.04 (1H, d, *J*=12.5 Hz), 4.71 (1H, dd, *J*=7.6, 5.2 Hz), 4.26–4.19 (1H, m), 3.36–3.39 (1H, m), 3.22 (1H, dd, *J*=15.0, 7.0 Hz), 2.53–2.39 (2H, m), 2.00 (3H, s), 1.94–1.98 (1H, m), 1.87–1.73 (1H, m); **^{13}C NMR (151 MHz, CD_3OD)**: (deuterated carbons are not seen due to long T_1 relaxation time) δ 172.6, 157.0, 136.7, 136.5, 128.1, 127.6, 127.5, 127.4, 123.2, 120.8, 118.3, 118.1, 109.2, 66.4, 54.1, 31.4, 29.6, 28.1, 27.0, 13.8; **HRMS** (ESI) calculated for $C_{24}H_{25}D_2N_3O_5SNa$ $[M+Na]^+$ 494.1695 found 494.1682 (33%); calculated for $C_{24}H_{24}D_3N_3O_5SNa$ $[M+Na]^+$ 495.1758 found 495.1741 (59%); calculated for $C_{24}H_{23}D_4N_3O_5SNa$ $[M+Na]^+$ 496.1820 found 496.1791 (8%); D_{Total} = 2.75 calculated by HRMS.

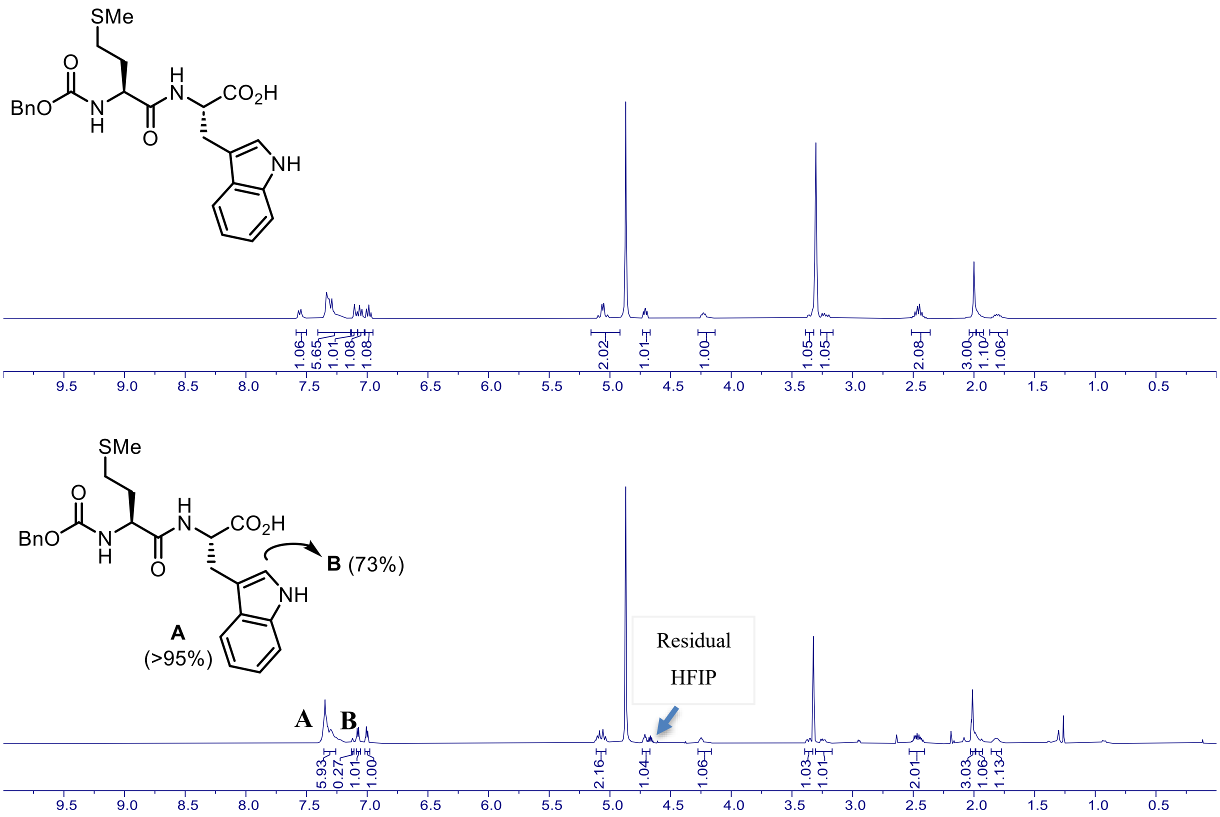

Figure S58. Stacked ^{1}H NMR (600 MHz, CD_3OD) spectra of **26**: natural abundance (top) and labelled (bottom).

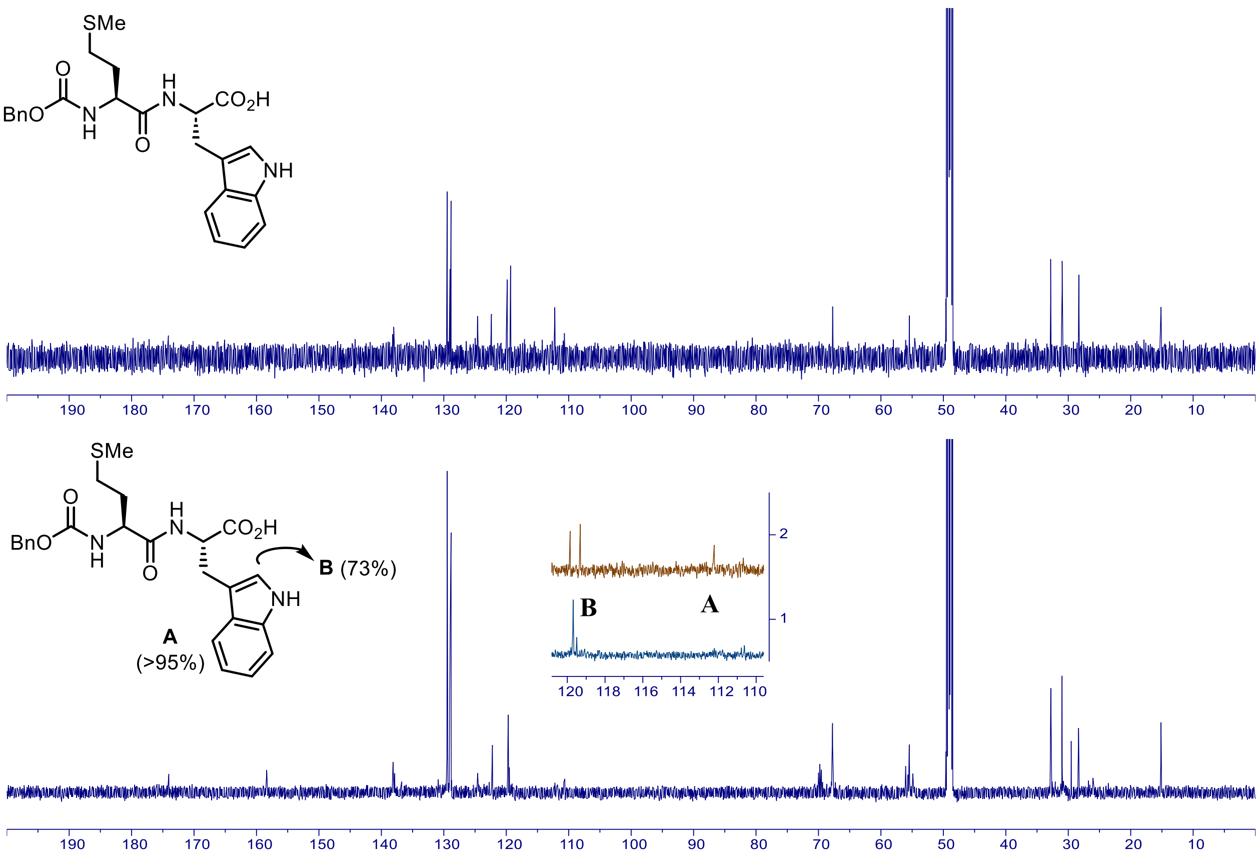

Figure S59. Stacked ^{13}C NMR (151 MHz, CD_3OD) spectra of **26**: natural abundance (top) and labelled (bottom).

[^{2}H]Vincamine (27)

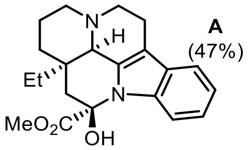

Obtained from vincamine following procedure **GP1** [254 nm]. (87%, D_{Total} = 0.47); **^{1}H NMR (600 MHz, $CDCl_3$)**: δ 7.50–7.48 (0.53H, m, labeled 47% D), 7.15–7.07 (3H, m), 4.63 (1H, s), 3.93 (1H, s), 3.83 (3H, s), 3.34 (1H, dd, *J*=13.5, 6.6 Hz), 3.29 (1H, td, *J*=13.5, 5.6 Hz), 3.06–2.93 (1H, m), 2.62 (1H, dt, *J*=8.7, 2.5 Hz), 2.58–2.48 (2H, m), 2.29–2.21 (2H, m), 2.13 (1H, d, *J*=14.2 Hz), 1.82–1.73 (1H, m), 1.73–1.65 (1H, m), 1.52–1.43 (2H, m), 1.41–1.33 (1H, m), 0.92 (3H, t, *J*=7.6 Hz); **^{13}C NMR (151 MHz, $CDCl_3$)**: δ 174.6, 134.2, 131.5, 129.1, 121.8, 120.4, 118.6 (s, non-labeled), 118.3 (t, *J* = 22.9 Hz, labeled 47% D), 110.4, 106.0, 82.0, 59.3, 54.4, 51.1, 44.7, 44.6, 35.2, 29.0, 25.3, 20.9, 17.0, 7.7; **HRMS** (ESI) calculated for $C_{21}H_{27}N_2O_3$ $[M+H]^+$ 355.2022 found 355.2020 (58%); calculated for $C_{21}H_{26}DN_2O_3$ $[M+H]^+$ 356.2085 found 356.2074 (42%); D_{Total} = 0.42 calculated by HRMS.

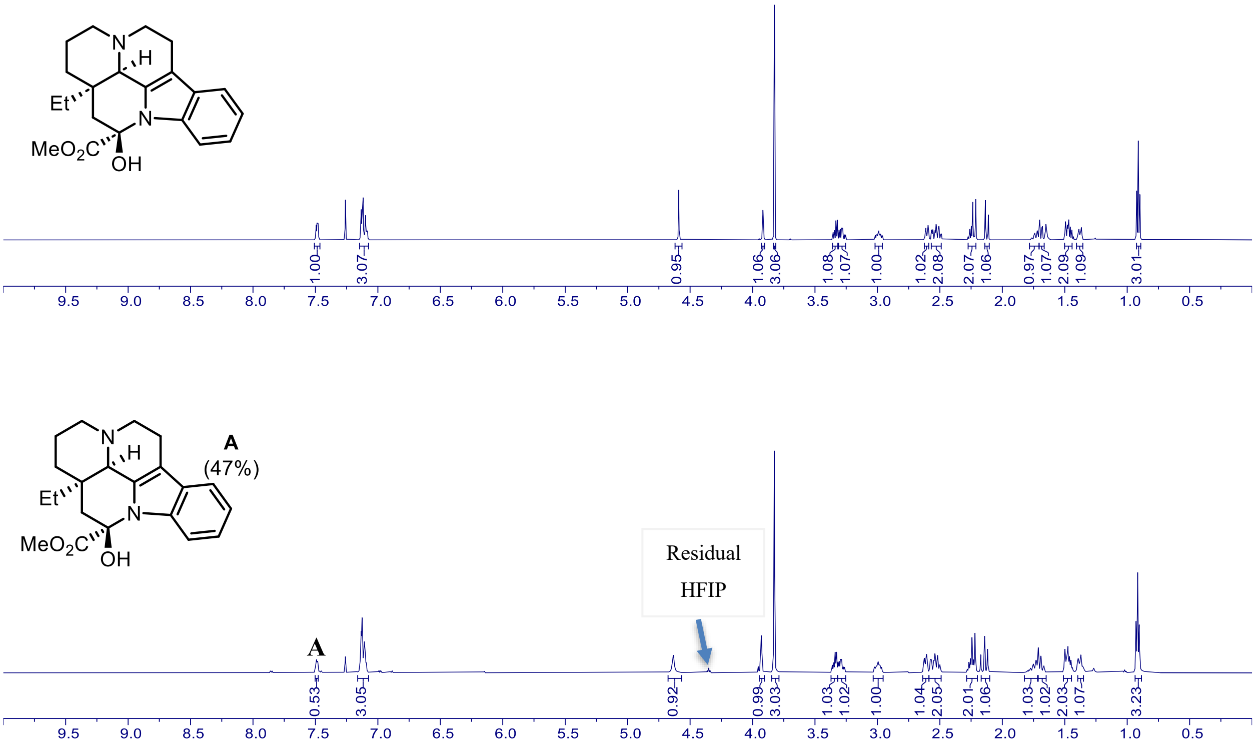

Figure S60. Stacked ^{1}H NMR (600 MHz, $CDCl_3$) spectra of **27**: natural abundance (top) and labelled (bottom).

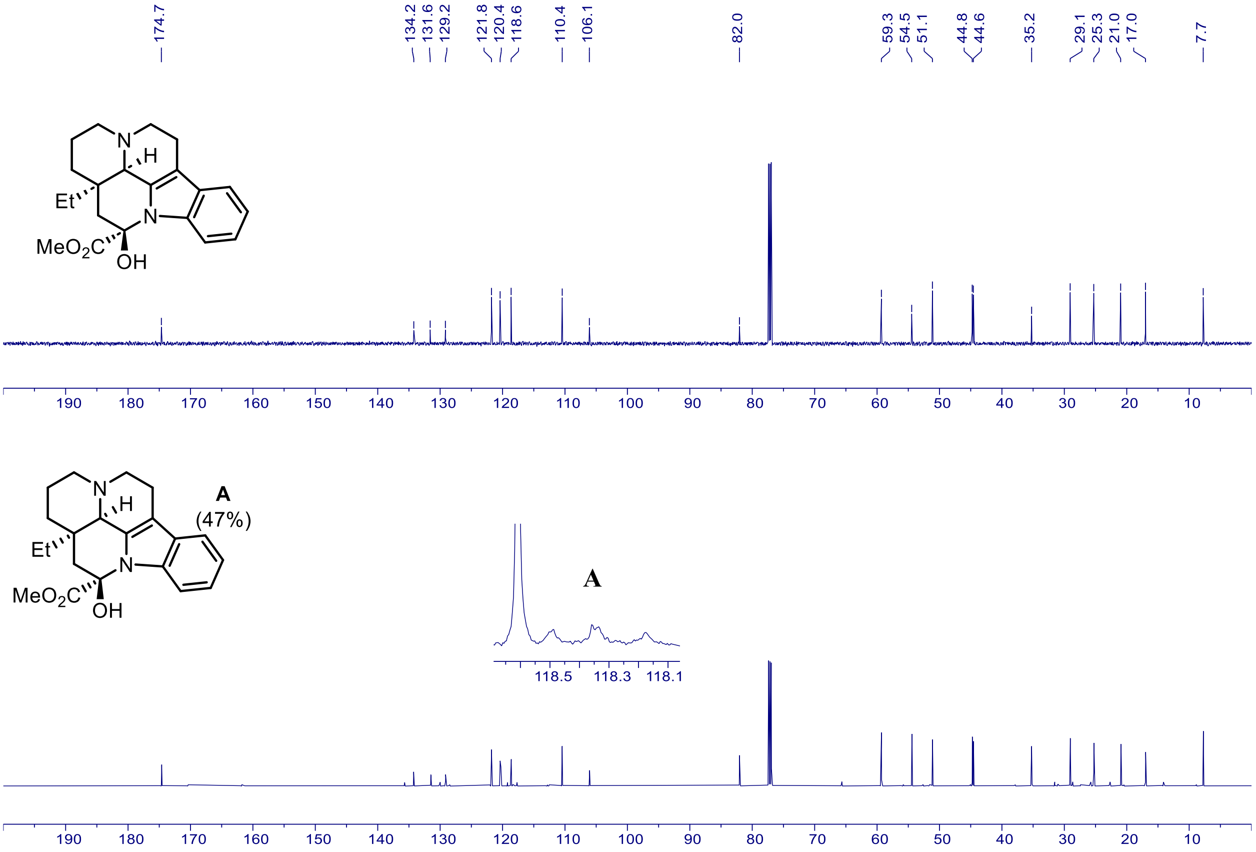

Figure S61. Stacked ^{13}C NMR (151 MHz, $CDCl_3$) spectra of **27**: natural abundance (top) and labelled (bottom).

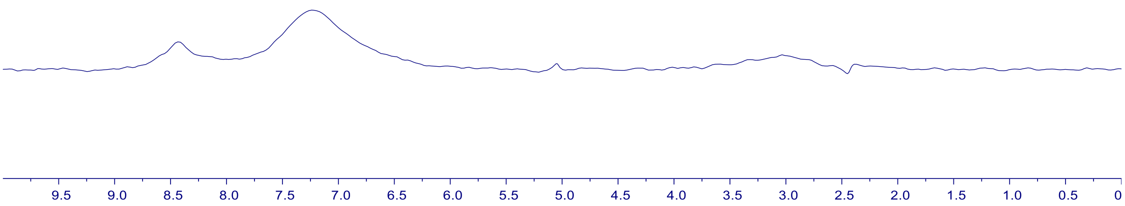

Figure S62. ^{2}H NMR (61 MHz, $CHCl_3$) spectra of **27**.

[^{2}H]Tadalafil (28)

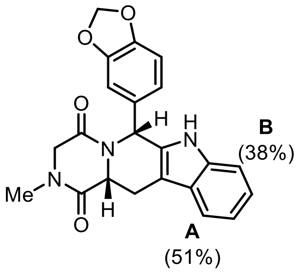

Obtained from tadalafil following procedure **GP1** [254 nm]. (62%, D_{Total} = 0.89); **^{1}H NMR (600 MHz, CD_2Cl_2)** δ 7.99 (1H, bs), 7.60 (0.59H, d, *J*=7.6 Hz, labeled, 51% D), 7.28 (0.62H, d, *J*=7.5 Hz, labeled, 38% D), 7.19–7.08 (2H, m), 6.87 (1H, dd, *J*=8.1, 1.9 Hz), 6.76 (1H, d, *J*=1.8 Hz), 6.70 (1H, d, *J*=8.0 Hz), 6.13 (1H, bs), 5.87 (2H, d, *J*=11.9 Hz), 4.31 (1H, dd, *J*=11.6, 4.5 Hz), 4.08 (1H, dd, *J*=17.4, 1.6 Hz), 3.91 (1H, d, *J*=17.4 Hz), 3.74 (1H, dd, *J*=16.0, 4.5 Hz), 3.17 (1H, dd, *J*=16.2, 11.7 Hz), 3.00 (3H, s); **^{13}C NMR (151 MHz, CD_2Cl_2)** δ 207.5, 167.2, 166.6, 148.3, 147.4, 137.0, 136.6, 133.5, 126.7, 122.7, 120.8, 120.3, 118.9 (labeled), 111.4 (labeled), 108.4, 107.5, 106.9, 101.7, 57.1, 56.5, 52.5, 33.7, 24.3; **HRMS** not found.

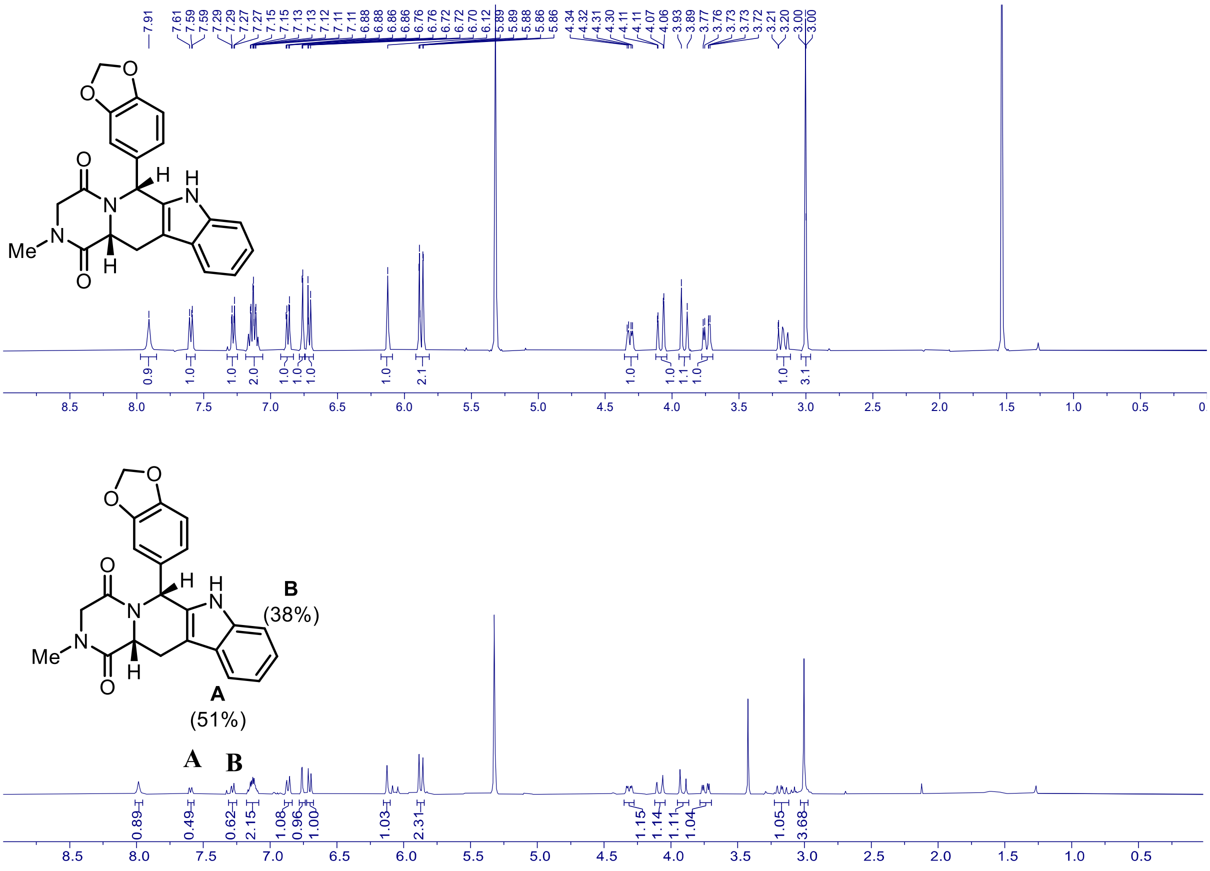

Figure S63. Stacked ^{1}H NMR (600 MHz, CD_2Cl_2) spectra of **28**: natural abundance (top) and labelled (bottom).

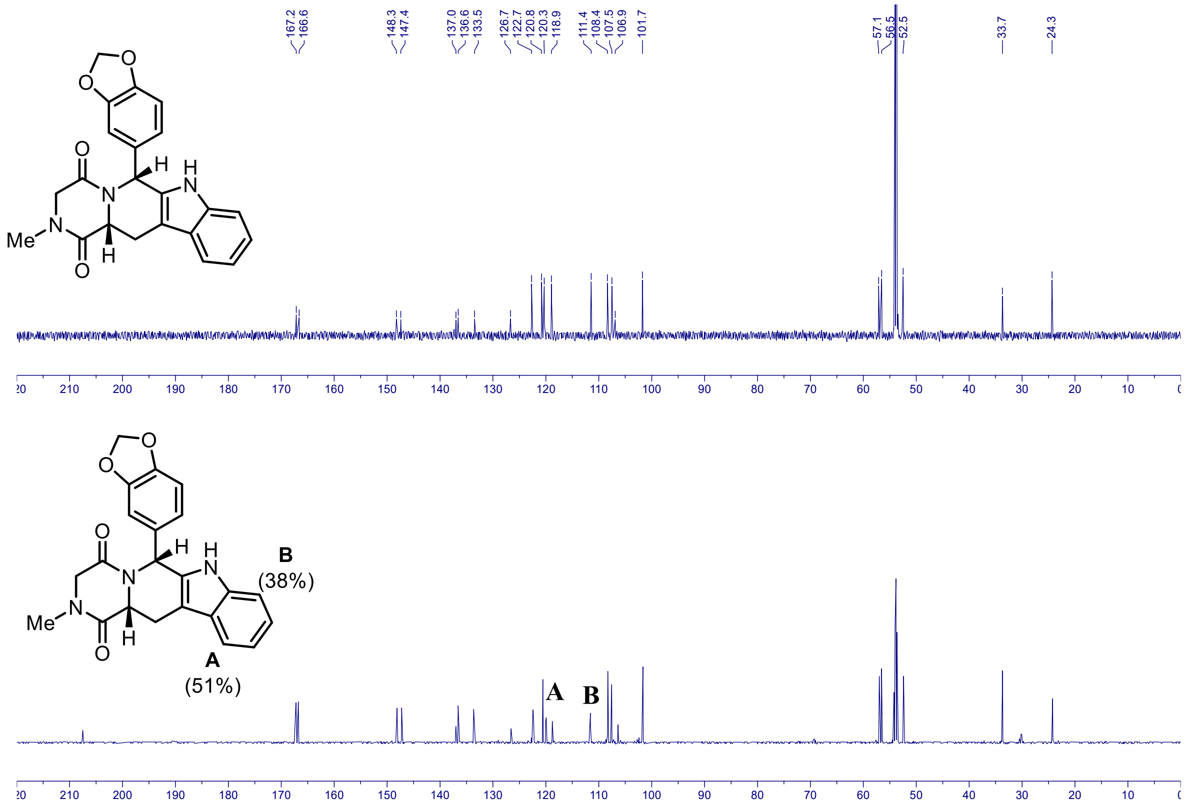

Figure S64. Stacked ^{13}C NMR (151 MHz, CD_2Cl_2) spectra of **28**: natural abundance (top) and labelled (bottom).

[^{2}H]Reserpine (29)

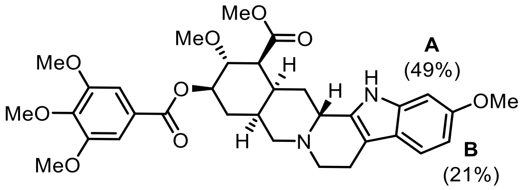

Obtained from reserpine following procedure GP1 [254 nm]. Purification by prep TLC. (80%, $D_{Total} = 0.7$); **^{1}H NMR (400 MHz, CD_2Cl_2)** δ 7.89 (1H, s), 7.34 (2H, s), 7.30 (1H, d, *J* = 8.5 Hz) 6.85 (0.51H, d, *J* = 2.0 Hz, labeled, 49% D), 6.77–6.68 (0.79H, m, labeled, 21% D), 5.07–4.97 (1H, m), 4.46 (1H, bs), 3.92–3.88 (7H, m), 3.86 (3H, s), 3.82 (3H, s), 3.81 (3H, s), 3.49 (3H, s), 3.20–3.08 (2H, m), 3.05–2.88 (2H, m), 2.69 (1H, dd, *J* = 11.2, 4.8 Hz), 2.52–2.40 (2H, m), 2.40–2.23 (2H, m), 2.03 (1H, dq, *J* = 13.2, 4.3 Hz), 1.99–1.76 (3H, m); **^{13}C NMR (101 MHz, CD_2Cl_2)** δ 172.6, 165.3, 156.1, 153.1, 142.2, 136.3, 130.8, 125.5, 122.2, 118.3, 108.8, 108.0, 106.7, 94.8–94.6 (m, labeled), 78.0, 77.8, 60.6, 60.5, 56.1, 55.6, 53.7, 51.9, 51.7, 51.2, 49.0, 34.2, 32.4, 29.6, 24.2, 16.8; **HRMS** (ESI) Calculated for $C_{33}H_{41}N_2O_9$ $[M+H]^+$ 609.2812 found 609.2805 (32%); Calculated for $C_{33}H_{40}DN_2O_9$ $[M+H]^+$ 610.2875 found 610.2861 (45%), Calculated for $C_{33}H_{39}D_2N_2O_9$ $[M+H]^+$ 611.2938 found 611.2913 (22%); ($C_{33}H_{38}D_3N_2O_9$ 1%); $D_{Total} = 0.92$ calculated by HRMS.

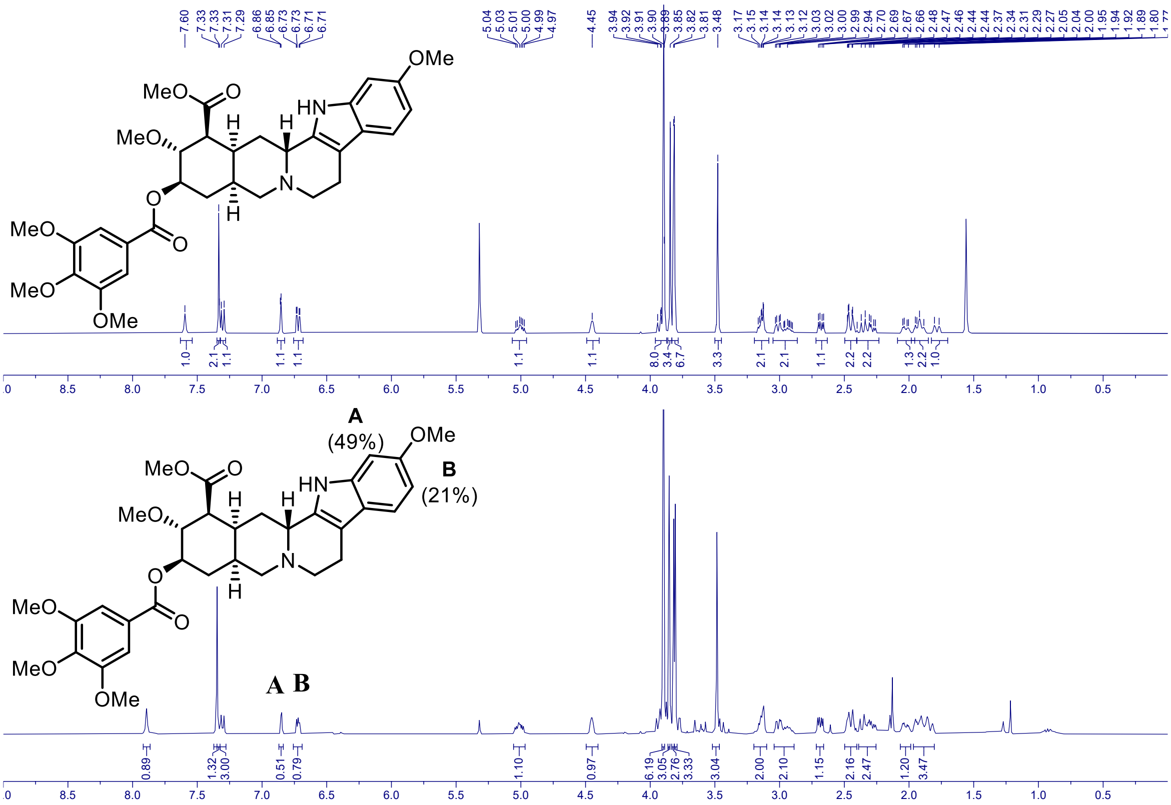

Figure S65. Stacked ^{1}H NMR (400 MHz, CD_2Cl_2) spectra of **29**: natural abundance (top) and labelled (bottom).

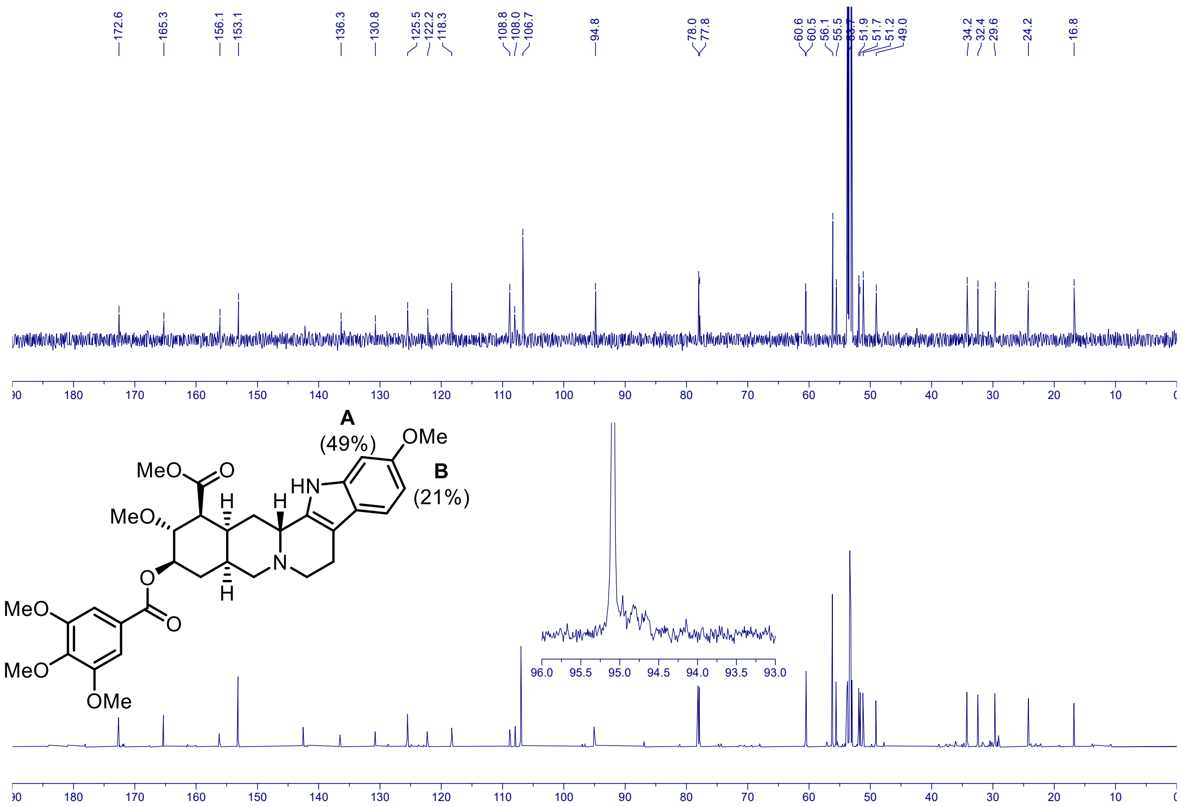

Figure S66. Stacked ^{13}C NMR (101 MHz, CD_2Cl_2) spectra of **29**: natural abundance (top) and labelled (bottom).

7. Gram-Scale Experiment

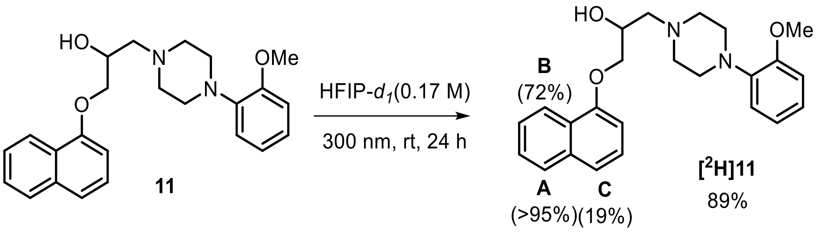

A round bottom flask equipped with a stirrer bar was charged with naftopidil (1.0 g, 2.55 mmol, 1.0 equiv.) and HFIP-d_1 (15 mL). The vial was placed in the photoreactor (300 nm) and irradiated for 24 h at room temperature with a fan. The tube was opened, and the solvent evaporated. The resulting crude was further purified by recrystallization using CH_2Cl_2/pentane yielding the titled compound as a white solid (890 mg, 89%). An aliquot of the solid was solubilised in $CDCl_3$ (0.6 mL) and analysed by ^1H NMR spectroscopy to obtain the ^2H incorporation. **Note: the %D was slightly different in comparison with the reaction run at 0.1 mmol due to the longer reaction time.**

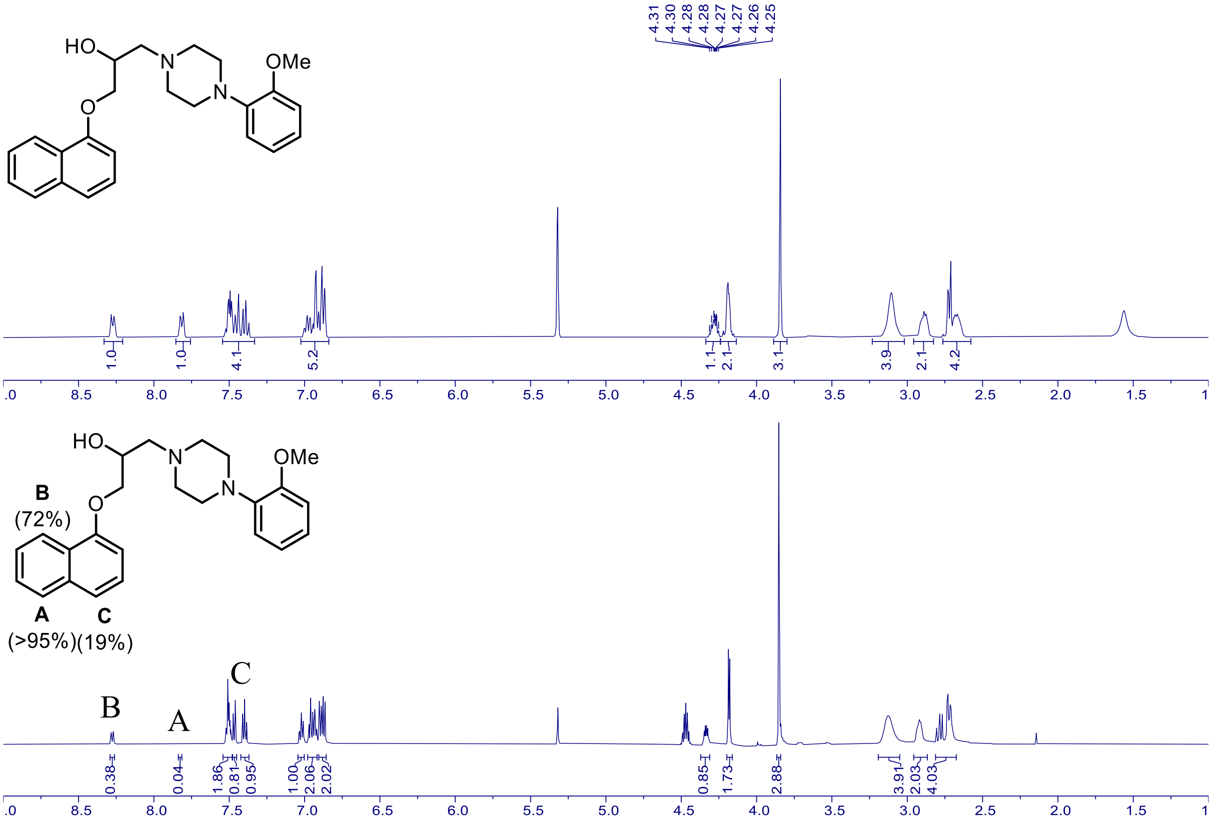

Figure S67. Stacked ^1H NMR (600 MHz, CD_2Cl_2) spectra of **11**: natural abundance (top) and labelled (bottom).

8. Control experiments

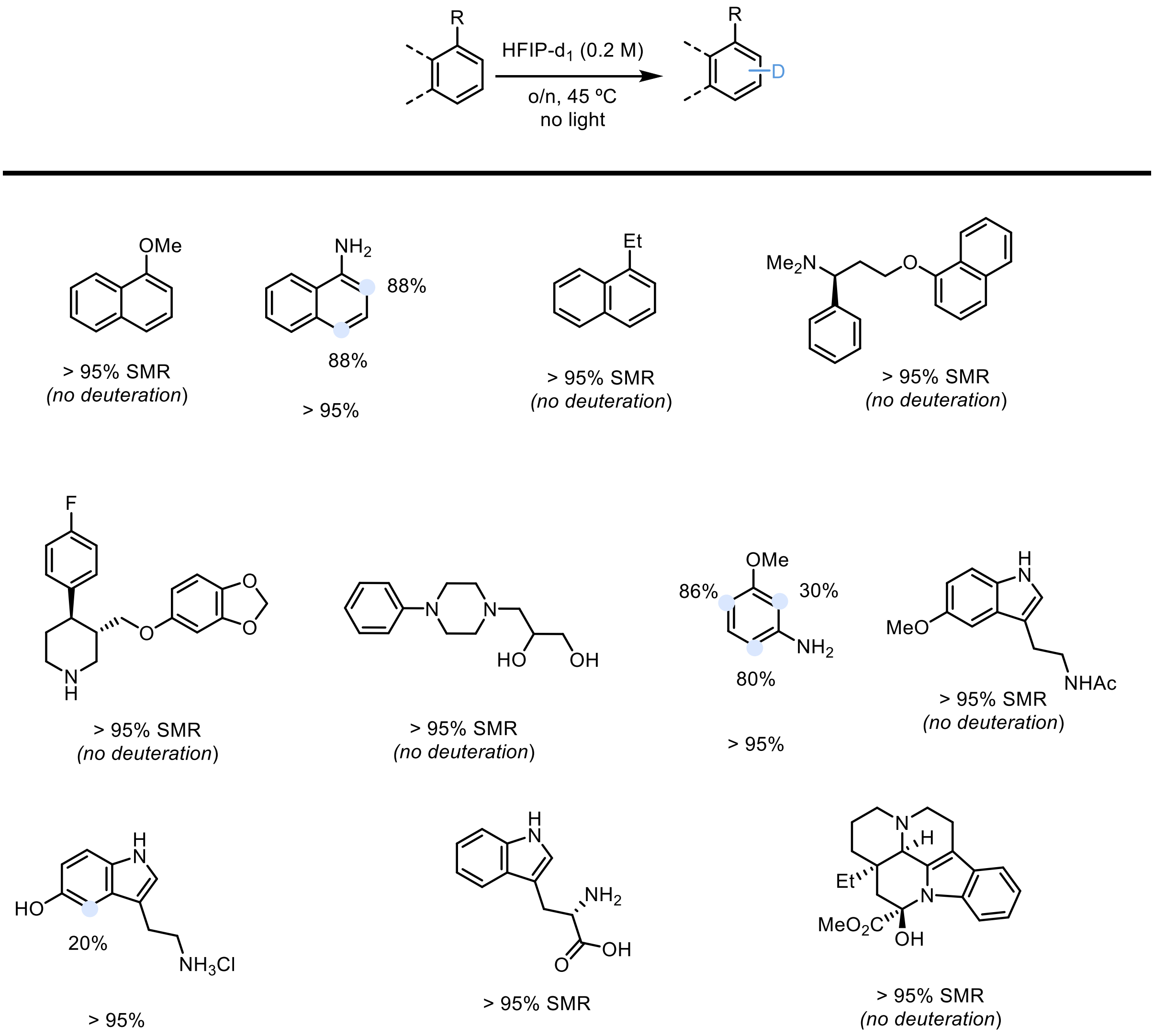

Figure S68. Control experiments.

An oven-dried 8 mL microwave was charged with the aromatic substrate (0.1 mmol, 1.0 equiv.) and HFIP-d$_1$ (0.5 mL). The vial was heated to 45 ºC and stirred for 16 h. The tube was opened, and the solvent evaporated. A solution of trichloroethylene (0.1 mL, 1.0 M in $CDCl_3$) was added, the crude was solubilised in $CDCl_3$ (0.6 mL) and the mixture analysed by ^{1}H NMR spectroscopy to obtain the ^{2}H incorporation and the NMR yield.

9. Photophysical studies

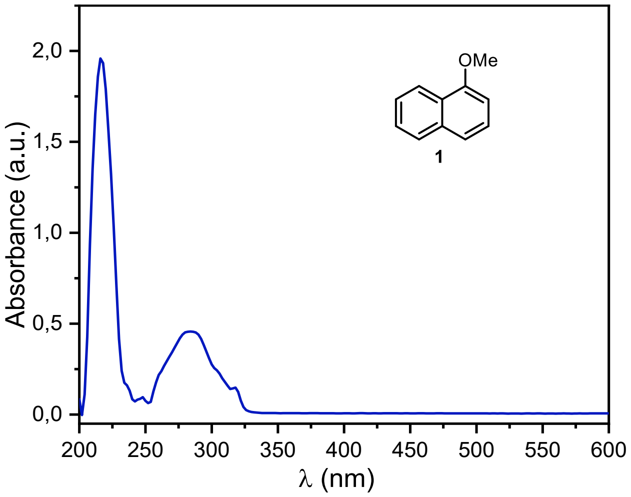

Figure S69. UV-vis spectra of **1** in HFIP (100 μM).

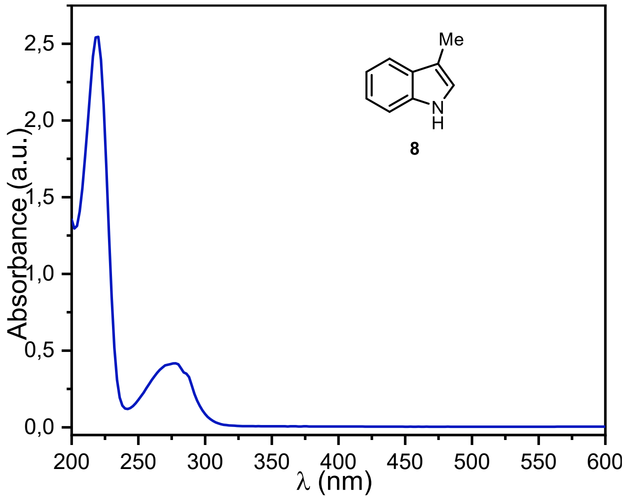

Figure S70. UV-vis spectra of **8** in HFIP (100 μM).
